# Metabolic Profiling of Circulating and Tumour‐Infiltrating T Cells Uncovers Bioenergetic Checkpoints Linked With Immune and Clinical Skewing in Melanoma Patients

**DOI:** 10.1111/imm.70185

**Published:** 2026-08-04

**Authors:** Hugo Brouque, Kareem Mahdy, Stephane Mouret, Sylvain Henry, Camille Niveau, Mélanie Cettour‐Cave, Martin Pelosse, Florence de Fraipont, Julie Charles, Andrei Turtoi, Philippe Saas, Caroline Aspord

**Affiliations:** ^1^ Institute for Advanced Biosciences, Team: Immunology & Immunotherapy of Chronic Diseases, Inserm U 1209, CNRS UMR 5309 Univ. Grenoble Alpes Grenoble France; ^2^ Etablissement Français du Sang Auvergne‐Rhône‐Alpes, R&D Laboratory Grenoble France; ^3^ Dermatology, Allergology & Photobiology Department, CHU Grenoble Alpes Univ. Grenoble Alpes Grenoble France; ^4^ Platform for Translational Oncometabolomics, Biocampus, CNRS, INSERM Université de Montpellier Montpellier France; ^5^ European Molecular Biology Laboratory Grenoble France; ^6^ Medical Unit of Molecular Genetic (Hereditary Diseases and Oncology) Grenoble University Hospital Grenoble France; ^7^ Tumor Microenvironment and Resistance to Treatment Lab, INSERM U1194 Montpellier France; ^8^ Division of Integrated Oncology Research Gunma University, Initiative for Advanced Research (GIAR) Maebashi Gunma Japan

**Keywords:** immunometabolism, melanoma, metabolomics, SCENITH, T cells

## Abstract

Metabolism is critical for immune cell functions. Tumours shape their microenvironment to render it metabolically hostile for infiltrating immune cells. While targeting immunometabolism emerges as a promising way to reinvigorate anticancer immunity, a deeper understanding of the metabolic disturbances of immune cells is needed. Here, we explored how the metabolic status of T cells governs immune skewing from circulating and tumour‐infiltrating CD4^+^ and CD8^+^ T cells of melanoma patients at a single‐cell level using the SCENITH method and targeted metabolomics. Circulating and tumour‐infiltrating T cells from patients displayed a decreased mitochondrial dependency associated with an enhanced glycolytic capacity and a skewed metabolic reprogramming upon stimulation. Such metabolic disturbances were linked to the activation status, immune checkpoint profile and functional orientation of T cells, underlining critical connections between T‐cell features and metabolic patterns. Targeted metabolomics within sorted CD4^+^/CD8^+^ T cells identified a decrease in citrulline, cysteine and threonine within all subsets in patients, together with a sharp rise in sterol cholesterol CE(20:2) and ceramide dhCer(d18:0/22:0) within tumour‐infiltrating CD4^+^ T cells, and in glycerolipid DG(16:0/16:0) within CD8^+^ T cells in blood and tumour. We further outlined a metabolic‐checkpoint‐based signature composed of six genes coding enzymes/transporters connected to the imbalanced metabolites found within tumour‐infiltrating T cells (*LIPA*, *DGKA*, *GLUL*, *SLC38A1*, *SLC7A7*, *GCH1*) that shape patients' clinical outcome. These findings outline the skewed bioenergetic profiles of T cells and depict metabolic checkpoints associated with immune subversion. Harnessing metabolic pathways is promising for developing innovative therapies to restore optimal anti‐tumour responses and improve clinical success.

## Graphical abstract

In this study, we revealed the disturbed metabolic status of circulating and tumour‐infiltrating CD4^+^ and CD8^+^ T cells in melanoma patients through targeted metabolomic and lipidomic (on sorted subsets) and at single cell level using the SCENITH method. We identified in patients a dampening in the amino acids citrulline and glutamine, together with a sharp rise in certain lipids such as sterol cholesterol CE(20:2) and the glycerolipid DG(16:0/16:0). Strikingly, we further outlined a metabolic checkpoint‐based signature composed of six genes coding enzymes and transporters closely connected to the imbalanced metabolites found within tumour‐infiltrating T cells (*LIPA*, *DGKA*, *GLUL*, *SLC38A1*, *SLC7A7*, *GCH1*) that critically shape patients' clinical outcome. Using SCENITH in an innovative set up, we associated T cell metabolism with phenotypic profile and functionality. Circulating and tumour‐infiltrating CD4^+^ and CD8^+^ T cells from patients displayed a marked decrease in mitochondrial dependency associated with an enhanced glycolytic capacity. Such metabolic disturbances affected patient clinical outcome. The activation status and immune checkpoint profile of T cells were linked with their metabolism, underlining critical connections between T‐cell features and metabolic patterns. This work outlines how the metabolic status of T cells governs immune skewing, and identifies a metabolic checkpoint‐based signature that critically shapes patients' clinical outcome. Such advance paves the way for appropriate tuning of metabolic pathways to rescue T cells from tumour hijacking, and thus overcome cancer‐associated immune subversion in order to restore potent antitumour responses and enhance the efficacy of current immunotherapies. Integration of metabolic regulation into the optimised development of cancer immunotherapies holds great promise to achieve better clinical success.

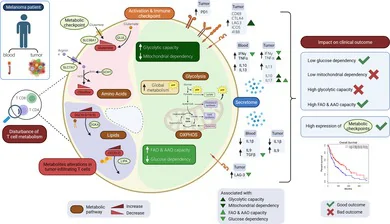

## 1 Introduction

Cancer immune escape is a major challenge for clinicians. Among therapeutic options, immunotherapies targeting immune checkpoints (ICP), known as ICP blockers (ICB), have revolutionised cancer treatment, achieving response rates of 45% in melanoma patients [1, 2]. The clinical success of ICB deployed in cancer strongly supports that the immune system can efficiently control tumour in the long term. Despite unprecedented successes, many patients experience side effects, relapse, or fail to respond to treatment [3]. This highlights the critical need to further explore the mechanisms leading to the failure of anti‐tumour responses to improve outcomes, enhance the efficacy of present immunotherapies, and develop more efficient therapeutic strategies [4].

Immune cell subsets, especially conventional T cells (Tconv) including CD4^+^ T helper (Th) cells and CD8^+^ cytotoxic T cells, are crucial for cancer immunosurveillance through their powerful cytotoxic capacity toward tumour cells and their secretion of effector cytokines that potentiate other immune cell subsets [5, 6]. Proportions of tumour‐infiltrating CD8^+^ T cells are associated with good prognosis and favourable outcomes in most cancers, including melanoma [7]. The immunoscore, based on the quantification of the density, location and functional orientation of CD3^+^ T cells within the tumour and its invasive margin, is highly pertinent to determine the risk of recurrence and predict response to treatment for patients with cancer [7]. This underlies the pivotal role of T cells in the control of tumour development. Such antitumour defence is impeded owing to the ability of cancers to evade immunosurveillance and adapt to immunological pressure [8, 9]. Tumours develop several mechanisms to escape T cell‐mediated immunity: camouflage through hiding from immune recognition or prevention of immune infiltration, coercion by suppressing the activity of effector T cells, as well as promoting immunosuppressive cells such as CD4^+^ regulatory T (Treg) cells through alteration of immunomodulatory ligands or release of immunomodulatory metabolites in the tumour microenvironment (TME) leading to T‐cell exhaustion and upregulation of inhibitor receptors (PD‐1, CTLA4, LAG3) [10]. Dismantling of antitumour defence can also occur through altered glycosylation motifs [11, 12, 13]. In melanoma patients, we previously demonstrated that effector T cells—including CD4^+^ and CD8^+^ T cells, γδ T cells, and invariant NKT cells—infiltrate tumours, but exhibited phenotypic modulations and functional alterations in both patients' tumours and blood [14, 15]. T cells displayed severe perturbations of ICP expression (deregulation of TIM3, LAG3, 41BB, PD1, ICOS), and are skewed toward Th2/Treg profiles [16, 17]. Moreover, we uncovered disturbed DC/effector crosstalk [15, 16], the crucial impact of effector T cell features on patients' clinical outcomes [14] and their ability to respond to immunotherapy [18]. Changes in TCR repertoire diversity in peripheral T cells were also a prognostic indicator of clinical outcome [19]. Thus, effector T cells harbour a crucial role in anti‐tumour responses and influence clinical outcomes. Further insights into the mechanism of T‐cell subversion and immunosuppression are thus essential to depict strategies of tumour immune evasion and better adapt the treatment according to the hijacking process within the TME [20].

There is increasing evidence for a role of metabolic reprogramming in T‐cell hijacking by tumours. Indeed, immunometabolism and energetic profile emerge as critical in the regulation of immune cell functions in cancer [21, 22, 23]. Immune cells rely primarily on two main pathways—oxidative phosphorylation (OXPHOS) and glycolysis—to produce ATP, mostly governed by AMPK and mTOR signalling pathways, respectively. Upon activation, immune cells undergo metabolic reprogramming to meet the bioenergetic demands required to trigger effector functions. Thus, metabolites and nutrients are crucial to sustain this metabolic switch from OXPHOS to glycolysis, which provides essential resources to support new protein synthesis for cell proliferation, activation and function. Different immune cell subsets have distinct metabolic requirements, and distinct T‐cell fates are imprinted by divergent metabolic programs [24] across time and space [25]. Naïve T cells have minimal metabolic activity, whereas newly activated effector T cells rapidly upregulate glucose uptake and glycolysis (i.e., glucose metabolism) as well as glutamine metabolism to produce intermediates for the tricarboxylic acid (TCA) cycle, generating energy and intermediates to fuel proliferation, trafficking and cytokine production. Mitochondrial biogenesis in long‐lived cells, such as memory T cells, allow increase in mitochondrial respiratory capacity and efficient usage of metabolic pathways under stress conditions such as nutrient deprivation, supporting long‐term persistence. Treg predominantly rely on fatty acid oxidation (FAO), which allows them to optimally function within the TME. Glutamine is a critical amino acid for cellular function, being converted into glutamate then α‐ketoglutarate which enters the TCA cycle, facilitating biosynthesis to fulfil requirements for cell proliferation and function. It is becoming clear that metabolism plays a significant role in driving distinct differentiation programs such as Th1, Th2, Th17 and Treg [24]. Thus, metabolism is critical in driving distinct differentiation programs, and metabolic reprogramming shapes immune cell differentiation and function.

The metabolic flexibility of immune cells can be tuned by their microenvironment to further guide the elicitation of inflammatory or tolerogenic fates, which are determinant to subsequently drive immunity or tolerance. The normal paths of immune cell differentiation and function, being highly dependent on energy fuel sources found in the microenvironment, are completely altered within tumours. Indeed, tumour cells undergo a metabolic switch from OXPHOS to glycolysis to support their active proliferation; this creates an environment metabolically hostile to infiltrating immune cells, with competition for limiting nutrients, acidity, hypoxia and accumulation of toxic metabolites in the TME that impair immune cell function. Metabolic adaptations of immune cells to survive compromise their functionality/effector function. Tumours create a suppressive metabolic microenvironment that supports the metabolism of tumour‐promoting cells (Treg, myeloid‐derived suppressive cells [MDSC], tolerogenic DC) and dampens immune cell functions, contributing to ineffective immune responses and cancer progression [23]. Overall, tumours, through a metabolically hostile microenvironment, manipulate the metabolism of immune cells to hijack their function and escape immune surveillance. The underlying mechanism of such hijacked T cell metabolism in patients remained to be elucidated.

Immune cell metabolic fitness is central to effective antitumour immunity, but is compromised by metabolic barriers imposed by the tumour. This dampens the capacity of effectors to mediate antitumour functions, resulting in failure of antitumour responses and response to immunotherapies. Metabolic pathways emerge as promising determinants of antitumour responses, yet they remain to be investigated. This prompted us to gain insights into how the metabolic landscape of immune cells governs immune skewing. Among strategies to depict cell metabolism, the flow cytometry‐based SCENITH (Single‐Cell ENergetIc metabolism by profiling Translation inHibition) technology offers a unique opportunity to assess the metabolism at a single‐cell level while combining with other cell phenotypes [26, 27]. SCENITH is based on measuring changes in protein synthesis (PS) under the action of metabolic inhibitors, directly within the cells by flow cytometry using puromycin (puro) incorporation. PS reflects the global metabolic activity of a cell, mainly upon glycolysis and OXPHOS processes. Inhibition of the glycolysis and OXPHOS metabolic pathways by specific inhibitors (2‐deoxy‐glucose [DG] and Oligomycin [O], respectively) leads to a drop in PS allowing a reliable determination of the metabolic pathways used. Here, we uniquely explore the metabolic features of circulating and tumour‐infiltrating CD4^+^ and CD8^+^ T cells in melanoma patients using the SCENITH method combined with phenotypic profile (activation status, ICP) and functionality, and explore extensive metabolite dynamics through targeted metabolomics and lipidomics (LC–MS) from sorted T cell subsets. We unveiled major metabolic disturbances within CD4^+^ and CD8^+^ T cells of patients, affecting various pathways (glycolysis, OXPHOS, amino acid, lipid metabolism) together with critical interplays between metabolic pathways and T cell features with clinical impacts. By determining the bioenergetic profiles of immune cell subsets, these findings depict metabolic checkpoints critical for tumour control or associated with immune subversion. Exploiting these metabolic pathways, endowed with a high functional flexibility, is promising for developing innovative therapies allowing to drive optimal anti‐tumour responses and improve clinical success.

## 2 Methods

### 2.1 Melanoma Patients and Healthy Donor (HD) Samples

All procedures were approved by Grenoble University Hospital (CHUGA), and accredited by the Ministry of Education and Research under the reference #AC‐2020–3959, #AC‐2014‐2094 and approval #2023‐A01722‐43. Mandatory written informed consent was obtained from all donors prior to their participation in this study and samples were analysed anonymously. Blood samples were obtained from healthy donors (HD, *n* = 28) and stage I–IV melanoma patients (*n* = 30). Lymph node or cutaneous metastatic tumours (*n* = 29) were obtained from melanoma patients. Clinical features of melanoma patients are stated in Table S1.

### 2.2 Peripheral Blood Mononuclear Cell and Tumour‐Infiltrating Cell Isolation

Peripheral blood mononuclear cells (PBMC) were obtained by Ficoll density gradient separation (Eurobio) from the blood of HD and melanoma patients. Cells were frozen and stored at −150°C until use. Tumour samples were cut into small fragments using a sterile scalpel and enzymatically digested with collagenase D (final concentration 2.5 mg/mL) and DNase I (final concentration 100 μg/mL) during 30 min at 37°C with 5% of CO_2_ followed by mechanical disruption. Cell suspensions were obtained by filtering the mixture through a 50 μm sieve with RPMI 10% fetal bovine serum (FBS), frozen and stored at −150°C before use. After thawing, tumour‐infiltrating cells were first cleared of tumour cells by an adhesion step in a 75 cm^2^ flask and further separated from residual tumour cells using a CD45^+^ positive selection isolation kit (StemCell Technologies) according to the manufacturer's instructions.

### 2.3 Cell Culture and Activation

PBMC and CD45^+^ tumour‐infiltrating cells were cultured in RPMI 1640 GLUTAMAX I supplemented with gentamicin (20 μg/mL), non‐essential amino acids (MEM 1X) (Invitrogen), sodium pyruvate (1 mM) (Sigma) (complete RPMI) and 10% heat‐inactivated FBS in a humidified incubator maintained at 37°C with 5% CO_2_ atmosphere. Cells were analysed at basal state or upon stimulation for 16 h with either phorbol 12‐myristate 13‐acetate (PMA, Sigma, 20 ng/mL) and ionomycin (iono, Sigma, 1 μg/mL) or anti‐CD3/CD28 antibodies. The phenotype, metabolism and functions of effector cells were then analysed as described below.

### 2.4 Metabolism Profiling Using the SCENITH Method

Up to 2.10^6^ PBMC or CD45^+^‐tumour infiltrating cells per well were seeded in 96‐well plates in RPMI 10% FBS for studying cell metabolism. At basal state or after activation, cells were treated during 30 min with DMSO (Control), 2‐Deoxy‐D‐Glucose (DG, 100 mM, Sigma), Oligomycin (Oligo, 1 mM, Ozyme), or a combination of these drugs at the final concentrations before mentioned. Puromycin (Puro, 10 μg/mL, Cayla) was added during 30 min after the metabolic inhibitor treatment. Cells were then washed in cold PBS, and stained with the fluorescent cell viability marker and primary conjugated antibodies against surface markers during 20 min at +4°C in PBS. After washing, cells were fixed and permeabilised using FOXP3 fixation and permeabilisation buffer (ThermoFisher) following manufacturer instructions. Intracellular staining of puro was performed by incubating cells during 30 min at 4°C in permeabilisation buffer with anti‐Puro‐AF488 monoclonal antibody (Merck).

### 2.5 Assessment of Activation Status and Immune Checkpoint Expression Profile of Effector Cells by Flow Cytometry

In parallel to puro labelling, cells were stained in order to define T cell subsets (including CD4^+^ and CD8^+^ T cells), their activation status and immune checkpoint expression. To depict T cell subsets, the following antibodies were used: BV570‐anti‐CD45 (BioLegend), APC‐H7 anti‐CD3, BV421 anti‐PanTCRγδ, PE or BV786 anti‐TCRδ2, PerCP‐Cy5.5 anti‐CD8 (BD Biosciences) together with Live and Dead‐PE‐Texas Red (ThermoFisher). Among alive CD45^+^ cells, Tconv were depicted as CD3^+^ PanTCRγδ^−^ TCRδ2^−^, and CD8^+^ and CD4^+^ T cell subsets were further depicted as CD3^+^CD8^+^ and CD3^+^CD8^−^, respectively. The activation status and immune checkpoint profile of T cells were analysed by surface staining using PE‐Cy5 anti‐CD69, PE anti‐CD27, BV650 anti‐PD1, PE‐Cy5 anti‐41BB, BV650 anti‐ICOS, FITC anti‐OX40 (BD Biosciences), APC anti‐CD25, PE‐Cy7 anti‐GITR, AF700 anti‐TIGIT, PE anti‐TIM3, PE‐Cy7 anti‐CTLA4, APC anti‐LAG3 (Thermofisher). After washing, cells were finally resuspended in BD Biosciences FACS lysing solution before analysis by flow cytometry. Data were acquired with LSRII cytometer and analysed with DIVA 9 software (BD Biosciences).

### 2.6 Analysis of the Functional Orientation of T Cell Subsets

#### 2.6.1 Intracellular Cytokine Labelling Combined to SCENITH Procedure

Following cell incubation with immune cell activators for 17 h, Brefeldin A (1 μg/mL) was added. After 2 h, cells were submitted to SCENITH procedure through treatment for 30 min with DMSO (Control), 2‐Deoxy‐D‐Glucose (DG, 100 mM, Sigma), Oligomycin (Oligo, 1 mM, Ozyme), or a combination of these two drugs. Puromycin (Puro, 10 μg/mL, Cayla) was then added during 30 min. Cells were then washed in cold PBS and stained with the fluorescent cell viability marker and primary conjugated antibodies against surface markers during 20 min at 4°C in PBS using BV570‐anti‐CD45 (BioLegend), APC‐H7 anti‐CD3, BV421 anti‐PanTCRγδ, PE or BV786 anti‐TCRδ2, PerCP‐Cy5.5 anti‐CD8 (BD Biosciences). Cells were further fixed and permeabilised using FOXP3 fixation and permeabilisation buffer (ThermoFisher) following the manufacturer's instructions. Dead cells were excluded with Live&Dead staining. Intracellular staining of puro and cytokines was finally performed using anti‐human fluorophore‐labelled antibodies AF488 anti‐Puro (Merck), PE anti‐IL‐13, PerCP‐Cy5.5 anti‐IL‐17, APC anti‐IFN‐γ, AF700 anti‐TNF‐α and BV650 anti‐IL‐10 (all from BD Biosciences) for 30 min at 4°C. After washing and fixation, stained cells were analysed using LSRII Flow Cytometer and FACSDiva software 9.

#### 2.6.2 Quantification of Secreted Cytokines/Chemokines by Luminex

Upon culture of cells for 20 h as previously mentioned, supernatants were collected and stored at −20°C until use. Cytokine/chemokine dosage was performed with the Human ProcartaPlex Mix&Match kit (ThermoFisher) following manufacturer's instructions and measured using the MagPix200 instrument (BioRad) with xPONENT software. To evaluate effector cell secretome, we used the ProcartaPlex Human Th1/Th2/Th9/Th17 Cytokine Panel 18plex to assess the concentrations of GM‐CSF, IFN‐gamma, IL‐1 beta, IL‐2, IL‐4, IL‐5, IL‐6, IL‐12p70, IL‐13, IL‐18, TNF‐α, IL‐9, IL‐10, IL‐17A (CTLA‐8), IL‐21, IL‐22, IL‐23, IL‐27. Data analysis was done with the ThermoFisher ProcartaPlex Application.

### 2.7 Metabolomic and Lipidomic Analysis Using Liquid Chromatography—Mass Spectrometry (LC–MS)

#### 2.7.1 Cell Sorting With MACSQuant TYTO

Cell sorting was performed using the MACSQuant Tyto Cell Sorter (Miltenyi Biotec) (Cell Imaging platform, ISBG). This microchip‐based technology utilises a micro‐electro‐mechanical system valve located within a closed and sterile cartridge (MACSQuant Tyto Cartridge) to sort cells at low pressure, ensuring gentle sorting conditions, preserving cell viability and minimising metabolic stress [28]. CD4^+^ and CD8^+^ T cell populations were gated based on fluorescent antibody staining and collected in the positive collection chamber of the cartridge. To depict T cell subsets, the following antibodies were used: Vio Bright 515 anti‐CD45, PE Vio 770 anti‐CD3, APC anti‐CD56, PE anti‐TCRγδ, Vio Blue anti‐CD4, APC Vio 770 anti‐CD8 together with 405/520 Viobility dye (Miltenyi Biotec). The sorted cells (mean purity: 98.1% for CD4^+^ T cells, 97.8% for CD8^+^ T cells) were incubated 45 min at 37°C with 5% CO_2_ in 10% complete RPMI medium. The cells were then washed in cold PBS (spin 5 min 500 g at 4°C), immediately resuspended in lysis solution (1000 μL methanol, 485 μL H_2_0, 15 μL formic acid) and stored at −80°C before subsequent lipidomic and metabolomic analysis.

#### 2.7.2 LC–MS Analysis

Sample were subjected to targeted metabolomics/lipidomics, as previously described [29, 30]. Data analysis and visualisation were performed using R software (version 4.4.1). Prior to statistical testing, peak areas were normalised by Total Ion Current (TIC) to correct for technical variations. Missing or zero values were imputed with a minimal positive constant before applying a log2 transformation to stabilise the variance and induce normality. Supervised Partial Least Squares Discriminant Analysis (PLS‐DA) was conducted using the *mixOmics* package. Log2‐transformed data were scaled to unit variance (*Z*‐score normalisation) to ensure equal weight for all features regardless of their absolute abundance. Differential abundance analysis was performed using the *limma* package. Linear models were fitted to the log2‐transformed data (without unit variance scaling) to preserve the biological variance structure, followed by empirical Bayes moderation of the standard errors. *p* values were adjusted for multiple comparisons using the Benjamini–Hochberg (BH) false discovery rate (FDR) method. The top 12 adjusted *p* values of this analysis were used to generate BarPlot using *ggplot2* package. Enrichment analysis was performed on the top 12 differences for each comparison and the identified pathway with *p* value < 0.05 were mapped. Both enrichment analysis and mapping were done on MetaboAnalyst 6.0 (https://www.metaboanalyst.ca/). Lipid classes and subclasses were identified on LIPID MAPS. The mean of log2 fold change (log2FC) of each class present in the top 12 was calculated to assess which class was impacted for each comparison.

### 2.8 Gene Expression Profiling Interactive Analysis (GEPIA)

We investigated the clinical relevance of pertinent enzymes and nutrient transporters using RNA sequencing data from the Cancer Genome Atlas (TCGA) dataset in the Skin Cutaneous Melanoma (SKCM) cohort (*n* = 461 patients) [31] using the web‐based GEPIA2 server (http://gepia2.cancer‐pku.cn/). We performed correlation analysis and patient survival analysis based on specific gene expression levels.

### 2.9 Statistical Analysis

Statistical analyses were performed with the GraphPad Prism Software (version 9.3.1), by using the non‐parametric one way ANOVA unmatched Kruskal–Wallis test followed by Dunn's multiple comparison post‐test, the non‐parametric two‐way ANOVA or the mixed‐effect model analysis test followed by Tuckey's multiple comparison post‐test, the non‐parametric two‐way repeated measures (RM) ANOVA test or the mixed‐effect model followed by Sidak's multiple comparison post‐test, and the non‐parametric Wilcoxon test. The significance threshold was placed at *p* < 0.05. Survival analyses (Kaplan–Meier), correlations and heat maps were performed using the survival (3.3.1), survminer (0.4.9), corrplot (0.95), gplots (3.1.3), ggbiplot (0.6.2), and RColorBrewer (1.1.3) packages using RStudio software R version 4.2.1.

## 3 Results

### 3.1 Circulating and Tumour‐Infiltrating T Cell Subsets From Melanoma Patients Harbour an Altered Metabolism When Compared With Circulating T Cell Subsets From Healthy Donors

To decipher the energetic metabolism of T cell subsets in melanoma, we explored the metabolic profiles of circulating and tumour‐infiltrating T cell subsets in patients compared to healthy donors (HDs) using the SCENITH method (Figure 1A). T cell subsets, that is, CD4^+^ and CD8^+^ T cells were first discriminated using a multiparametric flow cytometry approach (Figure S1A), and their incorporation of puro was measured in the presence of the inhibitors (2‐Deoxy‐Glucose [DG] and Oligomycin [O]) that abrogate the two major metabolic pathways for energy production, glycolysis and OXPHOS, respectively (Figure S1B). These measurements allowed assessing the global energetic status (in the absence of inhibitors, that is, the direct MFI of puro) and calculating the contribution of each pathway to the global metabolism, called dependencies and capacities (as described in [27]). Dependencies represent the direct contribution of each pathway to the general metabolism, while capacities depict the ability of cells to use a pathway when the other one is inhibited. We first explored T‐cell metabolic profiles at basal state. We found noteworthy differences in the metabolic patterns of T cells from melanoma patient in comparison with HDs (Figure 1B,D). Circulating CD4^+^ and CD8^+^ T cells, as well as tumour‐infiltrating CD8^+^ T cells from melanoma patients exhibited significantly higher levels of global metabolism compared with T cells from HDs (Figure 1C). Such changes were accompanied by metabolic switches of T cell subsets. Indeed, in patients compared to HDs, circulating and tumour‐infiltrating CD8^+^ T cells from patients displayed a decreased mitochondrial dependency associated with an enhanced glycolytic capacity, whereas CD4^+^ T cells exhibited a dampening in both glucose and mitochondrial dependencies concomitant to an enhanced glycolytic and fatty acid and/or amino acid oxidation (FAO/AAO) capacities in both blood and tumour (Figure 1E). Overall, these results reveal significant alterations of metabolic profiles of circulating and tumour‐infiltrating T cells in melanoma patients with a loss of dependencies and a switch toward glycolytic capacity, suggesting a forced metabolic flexibility and a potential role for immunometabolism in driving subversion of T cells by melanoma.

**FIGURE 1:**
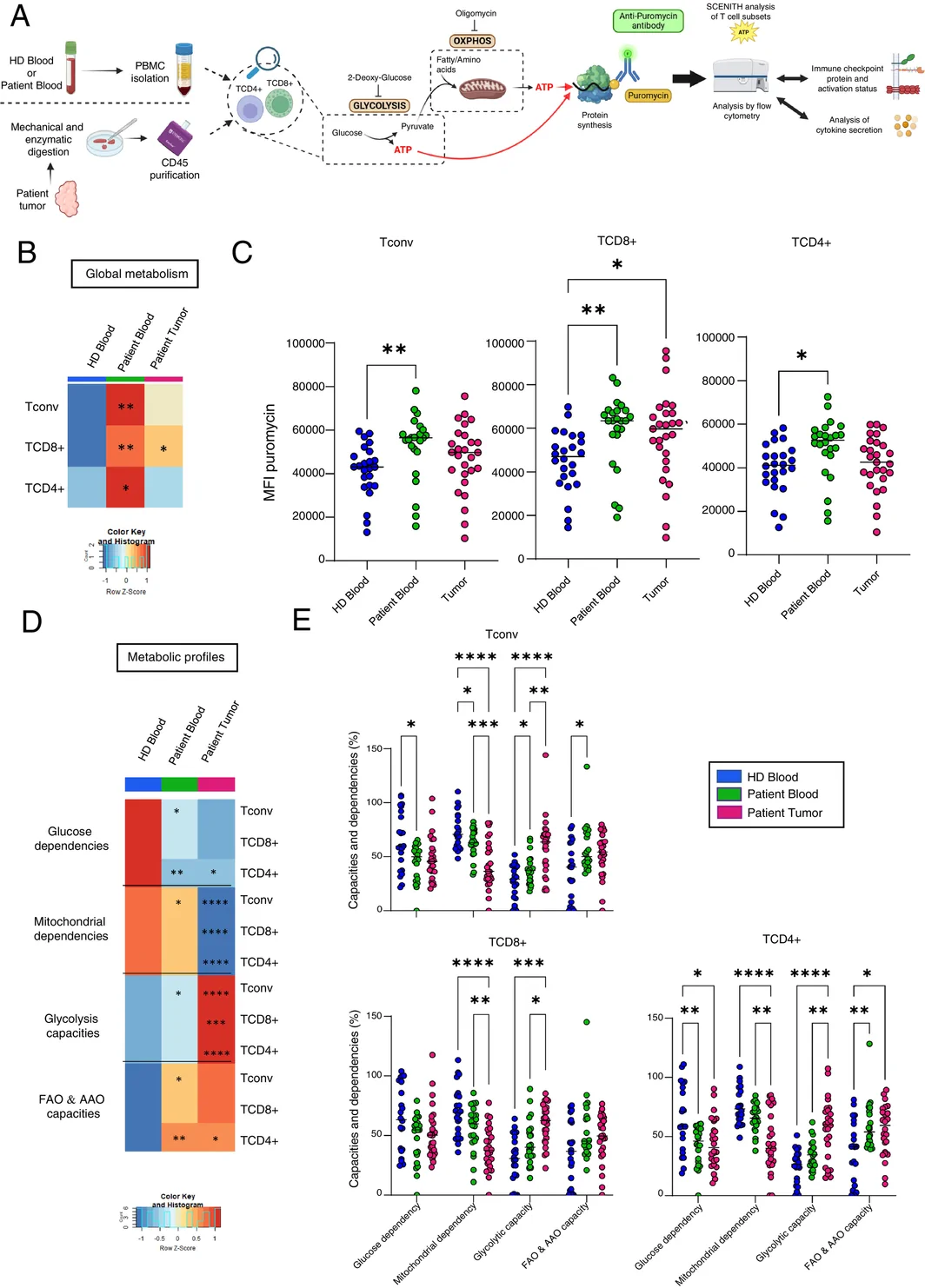
Circulating and tumour‐infiltrating T cell subsets from melanoma patients harbour a perturbed energetic metabolism in comparison with T cell subsets from healthy donors at steady state. PBMC from HDs and melanoma patients as well as CD45^+^ tumour‐infiltrating cells from patients were isolated and the metabolism of conventional T cells (Tconv) as well as CD8^+^ and CD4^+^ T cell subsets was determined by flow cytometry using the SCENITH method. (A) Experimental design. Created in BioRender. (B, D) Heat maps displaying the global metabolism (B) and metabolic profiles (D) of T cell subsets from HD blood, patient blood and patient tumour at basal state. Each square represents the median of each subgroup. (C, E) Comparative levels of global metabolism (mean fluorescence intensity (MFI) of puromycin) (C) and glycolytic or mitochondrial dependencies and capacities (E) of T cell subsets from HD blood (blue circles, *n* = 22–25), patient blood (green circles, *n* = 22–24) or tumour (pink circles, *n* = 26–27). Bars indicate median. *p* values were calculated using a Kruskal–Wallis test followed by Dunn's multiple comparison post‐tests (C), or non‐parametric two‐way ANOVA or a mixed effect model followed by Tukey's multiple comparison post‐tests (E). Only significant statistics are displayed on graphs. *p ≤ 0.05, **p ≤ 0.01, ***p ≤ 0.001, ****p ≤ 0.0001.

### 3.2 The Metabolism of T Cell Subsets Affects Patient Clinical Outcome

To further decipher whether the disturbed T cell metabolism affects patient clinical outcome, we compared overall survival (OS) and progression free survival (PFS) of patients according to the metabolic profiles of their circulating and tumour‐infiltrating T cell subsets (Figure 2, Figure S2 and Table S2). Interestingly, the metabolism of circulating CD4^+^ T cells revealed to be pivotal for clinical outcome in melanoma. A high glucose dependency or high glycolytic capacity of CD4 T cells were associated with shorter PFS and/or OS, whereas a high mitochondrial dependency or FAO/AAO capacity were linked with a better prognosis, when considering evolution from sampling time (Figure 2A) or diagnosis time (Figure 2A). When comparing early (stage I/II) and late (stage III/IV) clinical stages, we observed no difference in the global metabolism of circulating T cell subsets (Figure 2B). However, we noticed that T cells, especially CD8^+^ T cells, tended to display a dampened glucose dependency and an enhanced FAO/AAO capacity in late stage compared to early‐stage patients (Figure 2C). This suggests that the metabolic disturbances of circulating T cells could be worsening with the evolution of the disease. When separated according to previous treatment before tumour sampling, untreated patients and patients who received immunotherapy or targeted therapy (treated) displayed similar levels of global metabolism (Figure S2B) and similar metabolic dependencies/capacities (Figure S2C). Thus, prior treatment does not impact T cell metabolism. Interestingly, upon separation according to circulating LDH levels, CD4^+^ T cells from the ‘high LDH’ subgroup (i.e., associated with a poor prognosis) tended to display a lower global metabolism (Figure 2D), associated with dampened glucose/mitochondrial dependencies and enhanced glycolytic and FAO/AAO capacities compared to the ‘low LDH’ subgroup (Figure 2E). Altogether, these data suggest that the metabolism of T cell subsets in blood may influence the clinical outcome of melanoma patients, and the metabolic disturbances of circulating and tumour‐infiltrating T cells observed in patients worsened with the evolution of the disease.

**FIGURE 2:**
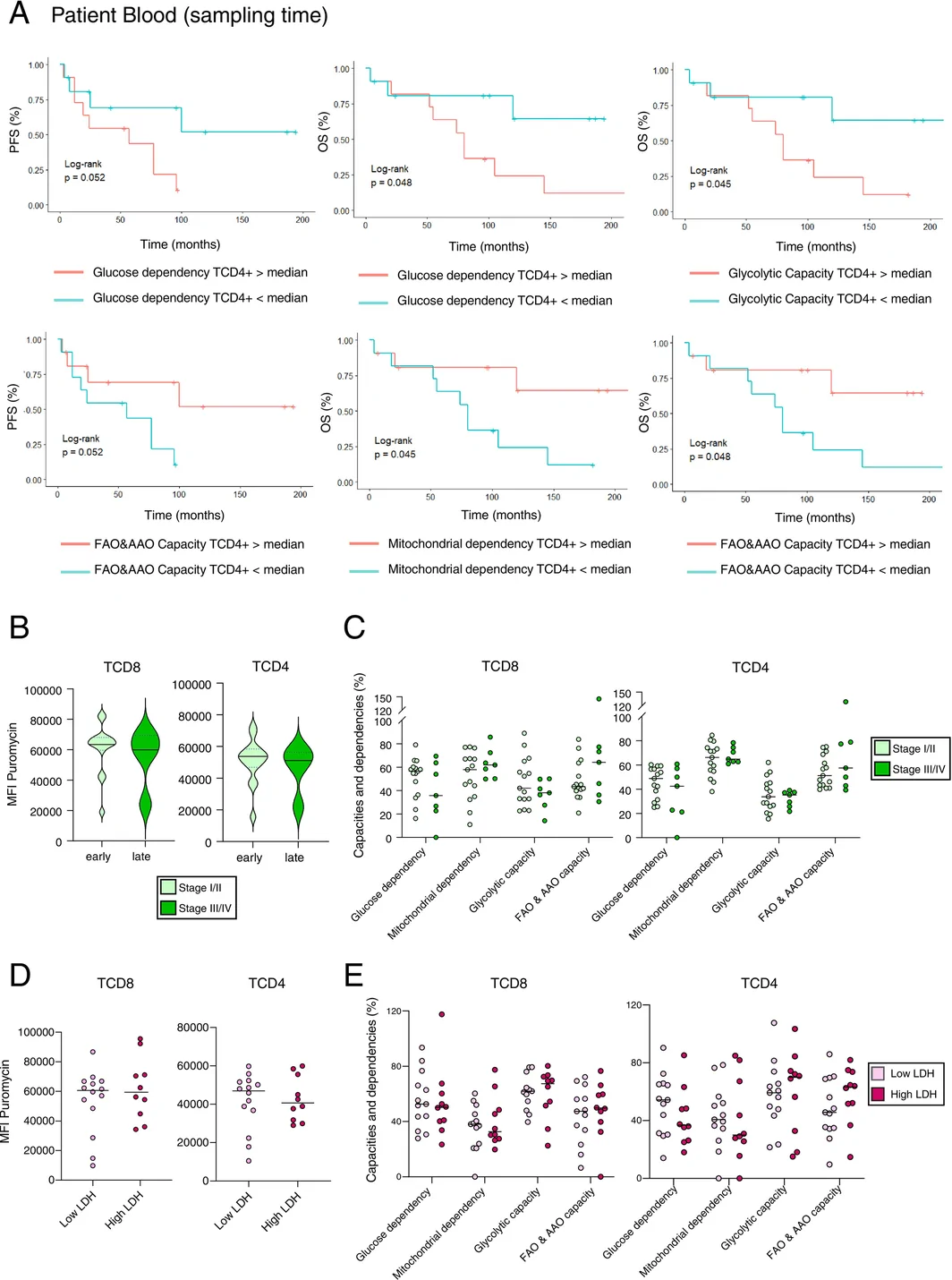
T cell subsets metabolism influences the clinical outcome of patients. PBMC from HDs and melanoma patients as well as CD45^+^ tumour‐infiltrating cells from patients were isolated and the metabolism of T cell subsets was determined by flow cytometry using the SCENITH method. (A) Comparative PFS and OS (from sampling time) of patients displaying circulating CD4^+^ T cells with a high or low glucose or mitochondrial dependency, or a high or low glycolytic or FAO/AAO capacity (5–6 patients per group). Groups were separated using the median of the corresponding parameter for circulating CD4^+^ T cells (glucose dependency [46.09%], mitochondrial dependency [65.5%], glycolytic capacity [34.5%], FAO/AAO capacity [53.91%]). Comparisons performed using Log‐Rank test. (B, C) Comparative levels of global metabolism (Mean Fluorescence Intensity (MFI) of puromycin) (B) and glycolytic or mitochondrial capacities/dependencies (C) of circulating CD8^+^ (left panel) and CD4^+^ (right panel) T cells for patients according to early (I/II) or late (III/IV) stage of disease evolution. (D, E) Comparative levels of global metabolism (Mean Fluorescence Intensity (MFI) of puromycin) (D) and glycolytic or mitochondrial capacities/dependencies (E) of tumour‐infiltrating CD8^+^ and CD4^+^ T cells according to circulating lactate dehydrogenase (LDH) level (low: ≤ standard; high: > standard).

### 3.3 The Activation Status of T Cell Subsets and Their Profile of Immune Checkpoints Are Linked With Their Metabolism

To explore the influence of metabolism on T cell subset features, we investigated simultaneously the metabolic profile of T cells and their expression of activation markers (CD69 and CD25), as well as ICP profile (CTLA4, PD1, LAG3, TIM3, ICOS, TIGIT, OX40, 41BB, CD27 and GITR) at basal state by flow cytometry (Figure S3A). Comparative expression of activation markers or ICPs by T cell subsets from HD blood, melanoma patient blood and tumour revealed a strong modulation in tumour‐infiltrating T cells (Figure S3B) as well as CD8^+^ and CD4^+^ T cells (Figure 3A). Tumour‐infiltrating CD8^+^ and CD4^+^ T cells showed enhanced proportions of CD69‐, PD1‐, LAG3‐ and ICOS‐expressing cells compared to the same cells present in HD and patient blood (Figure 3A). Additionally, tumour‐infiltrating CD4^+^ T cells exhibited increased proportions of CTLA4‐, and 41BB‐expressing cells in comparison to HD and patient blood (Figure 3A). Only ICPs expressed by more than 5% of T cells whatever the sample group were then considered; this excluded from analysis TIM3, TIGIT, OX40 and GITR. Correlations between the proportions of marker‐expressing cells in blood and tumour (Figure S3C) indicated that some modulations were linked together. For CD8^+^ T cells, strong positive correlations occurred between ICOS/41BB for circulating cells in patients, and CD69/41BB for tumour‐infiltrating T cells (Figure S3C). For CD4^+^ T cells, we observed a negative correlation between LAG3/CD27 in circulating T cells as well as strong positive correlations between CD69/ICOS and CD25/41BB for tumour‐infiltrating T cells (Figure S3C). Yet, most of the modulations were not linked together, underlining the heterogeneity of T‐cell phenotypic profiles in patients.

**FIGURE 3:**
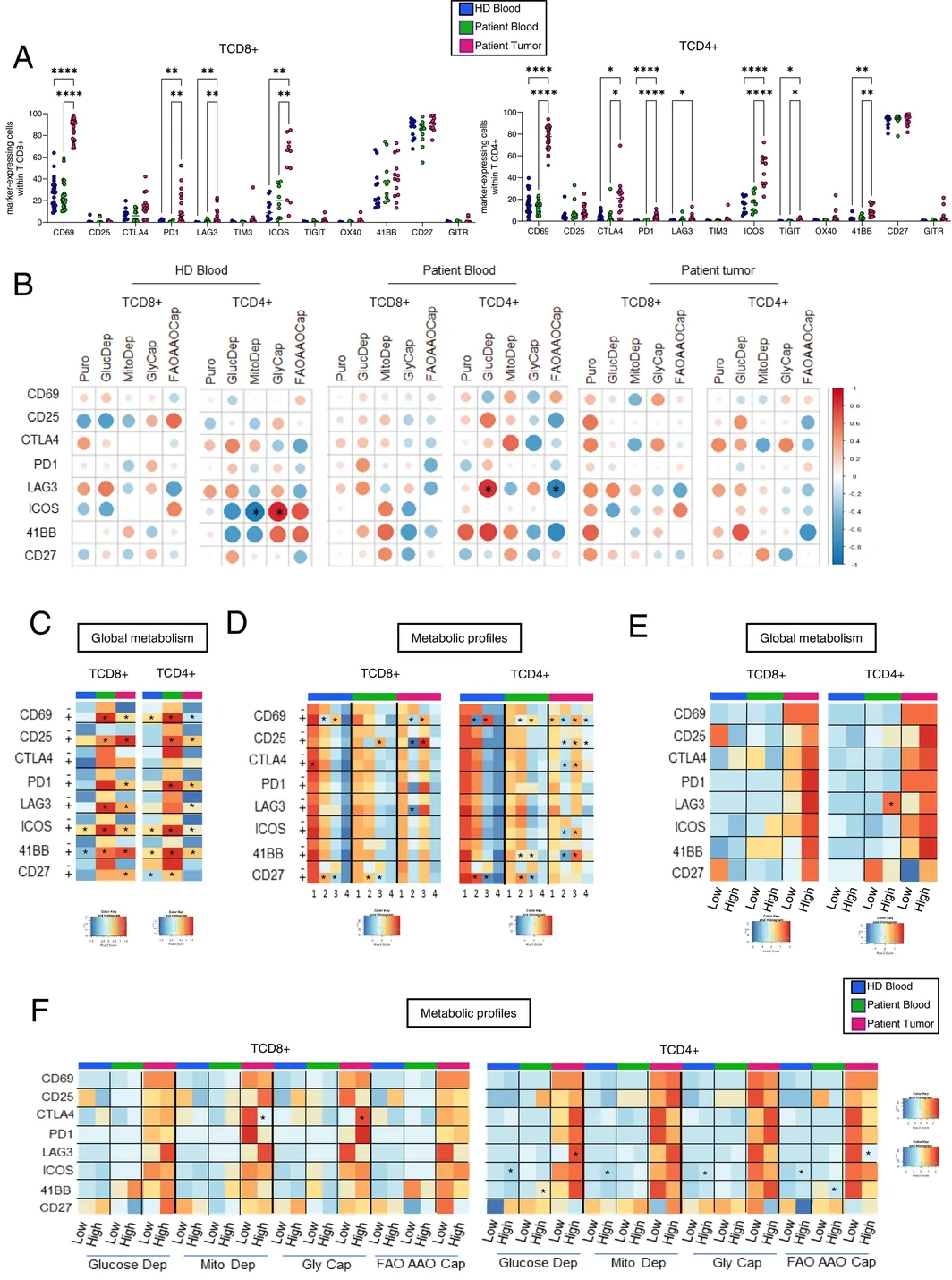
T cell subsets display distinct metabolic profiles depending on their activation status and their immune checkpoint profile. PBMC from HDs and melanoma patients as well as CD45^+^ tumour‐infiltrating cells from patients were isolated, and the metabolism of T cell subsets was determined using the SCENITH method together with their phenotypic features by flow cytometry. (A) Profile of expression of activation markers and immune checkpoints in CD8^+^ and CD4^+^ T cells from HD blood (blue, *n* = 11–25), patient blood (green, *n* = 10–24) and tumour (pink, *n* = 11–25). (B) Correlation matrices between the metabolic parameters of T cell subsets (global metabolism ‘puro’ as well as glycolytic or mitochondrial dependencies [GlucDep, MitoDep] and capacities [GlyCap, FAOAAOCap]) and their expression of activation markers and ICPs determined in HD blood (*n* = 10–25), patient blood (*n* = 9–24) or tumour (*n* = 11–25), respectively. *p* values were calculated using a Spearman test followed by Holm correction. Only significant statistical results are displayed on graphs. *p ≤ 0.01. (C, D) Heat maps displaying the (C) global metabolism and (D) glycolytic or mitochondrial dependencies/capacities of T cell subsets, stratified according to their expression (+) or not (−) of activation markers and ICPs for HD blood (blue, *n* = 10–11), patient blood (green, *n* = 9–10) or tumour (pink, *n* = 11–13). (D) Metabolic profiles were enumerated as follows: (1) glucose dependency, (2) mitochondrial dependency, (3) glycolytic capacity, and (4) FAO/AAO capacity. All significant statistics are displayed on corresponding graphs in Figures S6 and S7. (E, F) Heat maps displaying the expression levels of activation markers and ICPs on T cell subsets stratified according to their low or high (E) global metabolism and (F) glycolytic or mitochondrial dependencies (Glucose Dep, Mito Dep)/capacities (Gly Cap, FAO AAO Cap), for HD blood (blue, *n* = 10–11), patient blood (green, *n* = 9–10) or tumour (pink, *n* = 12–13). Groups were separated using the median of the metabolic parameter in the corresponding T cell subset. *P ≤ 0.05, **P ≤ 0.01, ****P ≤ 0.0001.

To assess the potential link between phenotype and metabolism, we performed correlation matrices between the proportions of activation marker‐/ICP‐expressing cells and metabolic activity and profiles of total T cells (Figure S4A) as well as CD8^+^ and CD4^+^ T cells (Figure 3B). Some ICPs were associated preferentially with glycolysis—such as LAG3 in CD4^+^ T cells from patient blood (Figure 3B)—or OXPHOS—such as 41BB in T cells from patient blood (Figure S4A)—revealing a metabolic pathway‐specific expression. Besides, the positive correlation between the proportion of ICOS‐expressing cells and glycolytic capacity in CD4^+^ T cells from HD blood (Figure 3B) was lost in patients. No correlation occurred in patient tumour, revealing more flexibility regarding the metabolic pathways sustaining ICP expression.

To get another view, we compared the metabolism of marker‐expressing (+) and marker‐negative (−) T cells. Heat maps based on the global metabolism level or percentages of dependencies/capacities overviewed the comparisons for T cells (Figure S4B,C) as well as CD8^+^ and CD4^+^ T cell subsets (Figure 3C,D). Individual graphs of comparison for each marker provided information on the statistical significance for CD8^+^ T cells (Figure S5) and CD4^+^ T cells (Figure S6). For most activation markers and ICP (except CD27), we observed a higher global level of metabolism in marker‐expressing T cells versus marker‐negative T cells from blood and tumour of patients, as illustrated by significant differences for CD69, CD25, PD1, ICOS, LAG3 and/or 41BB in CD4 and CD8 T cells. In contrast, such observation was depicted only for ICOS in HD blood (Figure 3C and Figure S4B). This shows that the expression of activation markers and ICP (except for CD27) in T cell subsets in the melanoma microenvironment is linked to a higher level of global metabolism.

Concerning the metabolic profiles associated with expression of activation markers or ICP, a similar profile was observed for CD69^+^ cells whatever the T cell subsets and the sample group with a decreased mitochondrial dependency associated with a higher glycolysis capacity, while an inverse profile (i.e., a higher mitochondrial dependency associated with a decreased glycolysis capacity) was observed for CD27 for CD4^+^ and CD8^+^ T cells from HD and patient blood (Figure 3D; Figures S4C, S5 and S6). Change in metabolic profiles according to expression of activation markers or ICP was mainly found for tumour‐infiltrating CD4^+^ T cells (Figure 3D and Figure S4C); we observed a decreased mitochondrial dependency associated with a higher glycolysis capacity in CD25‐, CTLA4‐, ICOS‐ or 41BB‐expressing CD4^+^ T cells and in CD25‐ or LAG3‐expressing CD8^+^ T cells versus marker‐negative CD4^+^ or CD8^+^ T cells in the tumour. Globally, perturbed metabolic profiles were preferentially detected in activation marker‐ or ICP‐expressing tumour‐infiltrating T cells.

Conversely, we analysed the phenotypic features of T cells according to low or high global metabolism (Figure 3E and Figure S4D), as well as low or high metabolic capacities/dependencies (Figure 3F and Figure S4E). In HD blood, few variations were observed according to the global metabolism of T cell subsets (Figure 3E), except for CD69 and CD25 expressed by total T cells (Figure S4D). In contrast, in patient blood, metabolically active T cells exhibited higher levels of LAG3 for CD4^+^ T cells (Figure 3E) and CD25 for total T cells (Figure S4D). In patient tumour, T cells with a higher metabolism displayed higher expression of CTLA4 (Figure S4D). Comparison of phenotypic features according to metabolic profiles revealed differences especially for tumour‐infiltrating CD8^+^ T cells and for circulating and tumour‐infiltrating CD4^+^ T cells (Figure 3F). In patient tumour, CD8^+^ T cells with a lower mitochondrial dependency and a higher glycolysis capacity displayed higher levels of CTLA4, while CD4^+^ T cells with a high glucose dependency and low FAO/AAO capacity displayed higher levels of LAG3 (Figure 3F). In patient blood, CD4^+^ T cells with a high glucose dependency and low FAO/AAO capacity displayed higher levels of 41BB (Figure 3F). These observations highlighted that T cell subsets display distinct metabolic profiles depending on their activation status and ICP profile, underlining critical connections between T‐cell features and metabolic patterns.

### 3.4 The Cytokine Secretion Profile of T Cells Is Linked With Their Metabolism

To further investigate whether the metabolism can influence the functional status of T cells, we explored the secretome of T cells at basal state together with their metabolic features. Among the large panel of cytokines examined, TGF‐β was the most abundant factor produced at steady state, exhibiting an increase with circulating T cells of patients, as well as a decrease with tumour‐infiltrating T cells compared to HDs (Figure S7A). Even though not significant, we also observed an increase of IL‐1β and IL‐10 production by circulating T cells of patients. To assess a potential link between functional profile and metabolism, we performed correlations between cytokine secretion levels and metabolic features of T cells (Figure S7B). In patient blood, we observed a strong positive correlation between IL‐1β or IL‐10 and global metabolism for CD8^+^ T cells, which were not present in HD blood and patient tumour. Interestingly, we also noticed in tumour a tendency toward a positive link between cytokine production and glycolytic capacity of the cells, while a negative association with mitochondrial dependency of the cells. These observations suggest a link between the metabolic status of T cell subsets and the composition of their secretome in accordance with their microenvironment.

### 3.5 Metabolic Reprogramming Induced by Stimulation of T Cells Strongly Differs in Melanoma Patients Compared to Healthy Donors

To assess the metabolic changes in response to stimulation, we stimulated T cells with anti‐CD3/CD28 antibodies or PMA/iono for 20 h, and further evaluated their metabolism using the SCENITH method (Figure 4A). We observed striking differences in the metabolic patterns of T cells from melanoma patient blood and tumour in comparison with HD blood (Figure 4B–E). Stimulation triggered an enhancement of the global metabolic activity of T cell subsets in all groups (Figure 4C). Yet, circulating CD4^+^ and CD8^+^ T cells from patients exhibited a much higher rise in global metabolism, while such enhancement was only slightly higher for tumour‐infiltrating T cells compared to HD (Figure 4C). Regarding metabolic profiles, stimulation induced drastically different metabolic switches in T cells from HD and patients (Figure 4D,E). Indeed, CD8^+^ and CD4^+^ T cells from patients strongly dampened their glycolytic dependency and increased their FAO/AAO capacity compared to HD upon PMA/iono and/or anti‐CD3/CD28 stimulation for circulating and tumour‐infiltrating T cells (Figure 4D,E). Overall, these observations support a deregulation in metabolic pathway usage upon stimulation by circulating and tumour‐infiltrating CD8^+^ and CD4^+^ T cells of melanoma patients.

**FIGURE 4:**
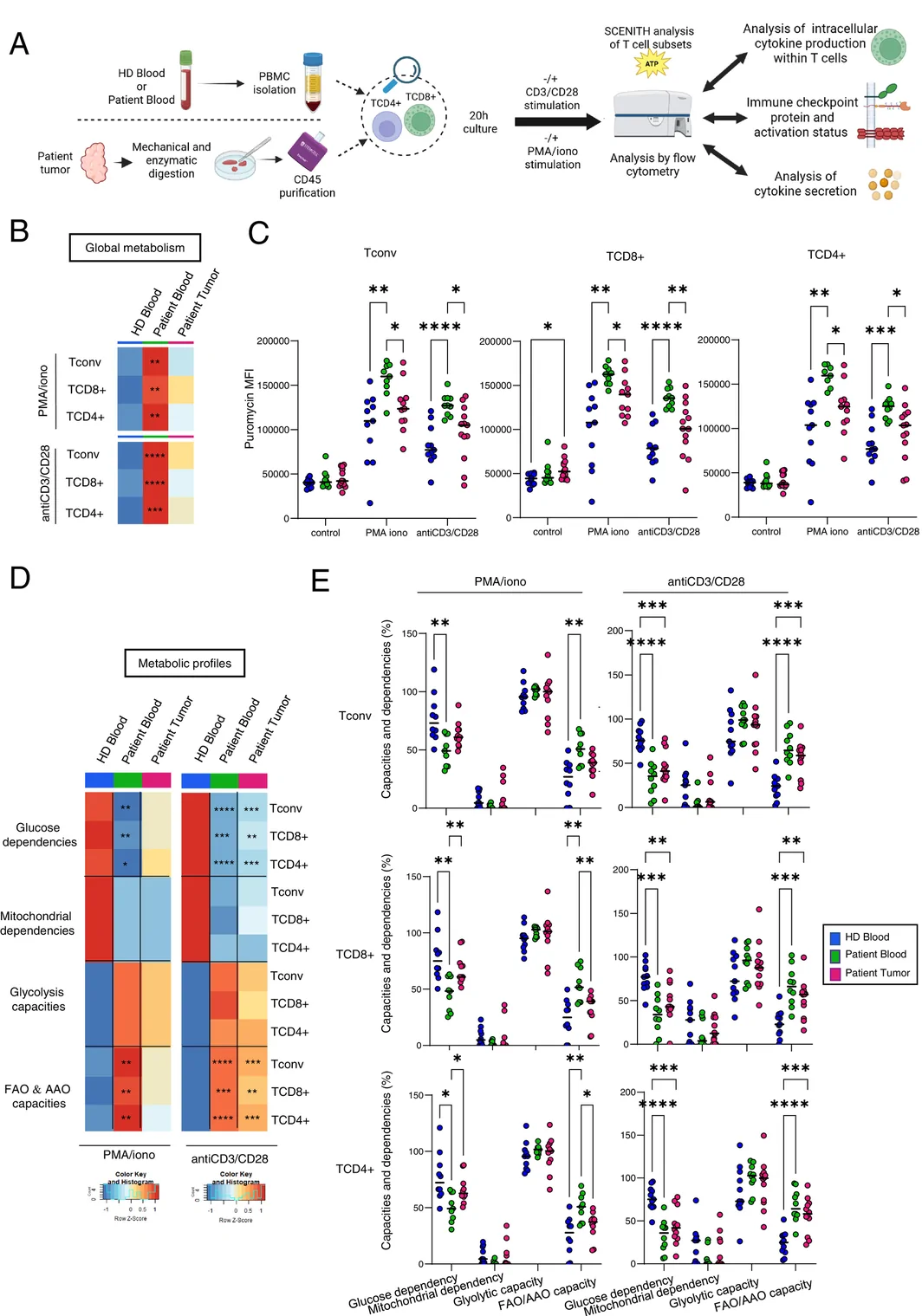
Circulating and tumour‐infiltrating T cell subsets from melanoma patients exhibit a perturbed metabolic reprogramming in response to stimulation in comparison with T cell subsets from healthy donors. PBMC from HDs and melanoma patients as well as CD45^+^ tumour‐infiltrating cells from patients were stimulated or not with either PMA/iono or anti‐CD3/CD28 antibodies for 20 h, and the metabolism of total T cells (Tconv), and CD4^+^ and CD8^+^ T cells was determined by flow cytometry using the SCENITH method. (A) Experimental design. Created in BioRender. (B, D) Heat maps displaying (B) the global metabolism (Mean Fluorescence Intensity (MFI) of puromycin) and (D) the glycolytic or mitochondrial dependencies and capacities of T cell subsets from HD blood, patient blood and patient tumour upon PMA/iono (left) or anti‐CD3/CD28 (right) stimulation. Each square represents the median of each subgroup. (C) Comparative levels of global metabolism (mean fluorescence intensity (MFI) of puromycin) and (E) glycolytic or mitochondrial dependencies/capacities of T cell subsets upon stimulation from HD blood (blue, *n* = 10–11), patient blood (green, *n* = 9–10) or tumour (pink, *n* = 11–12). Bars indicate median. *p* values were calculated using (C) a Kruskal–Wallis test followed by Dunn's multiple comparison post‐tests, or (E) a non‐parametric two‐way ANOVA or a mixed effect model followed by Tukey's multiple comparison post‐tests. Only significant statistic results are displayed on graphs. *p ≤ 0.05, **p ≤ 0.01, ***p ≤ 0.001, ****p ≤ 0.0001.

### 3.6 The Distinct Remodelling of Activation Markers and Immune Checkpoints Expressed by T Cell Subsets Upon Stimulation Is Linked With Differential Metabolic Reprogramming in Melanoma

To explore how disturbed metabolic switches of T cell subsets observed in patients impact their phenotypic features, we investigated simultaneously metabolic profiles of T cells and expression of activation markers, as well as after stimulation (Figure 5 and Figure S8). We observed major changes in expression profiles on circulating and tumour‐infiltrating whole T cells (Figure 5A), as well as CD8^+^ and CD4^+^ T cells (Figure S8) from patients. Circulating CD8^+^ and CD4^+^ T cells from patients contained enhanced proportions of CD25‐, TIM3‐, and 41BB‐expressing cells compared to HD blood whatever the stimulation. Additionally, CD8^+^ T cells from patient blood exhibited increased proportions of CD69‐, LAG3‐, ICOS‐, and OX40‐expressing cells compared to HD blood, according to the type of stimulation (Figure 5A and Figure S8). Tumour‐infiltrating, CD8^+^ and CD4^+^ T cells contained enhanced proportions of PD1‐, 41BB‐, and GITR‐expressing cells compared to HD blood whatever the stimulation used, together with increased proportions of TIM‐3‐expressing cells after anti‐CD3/‐CD28 stimulation (Figure 5A and Figure S8). Additionally, in tumour, CD8^+^ T cells included increased proportions of ICOS‐expressing cells, while CD4^+^ T cells comprised decreased proportions of CD27‐expressing cells compared to HDs (Figure S8).

**FIGURE 5:**
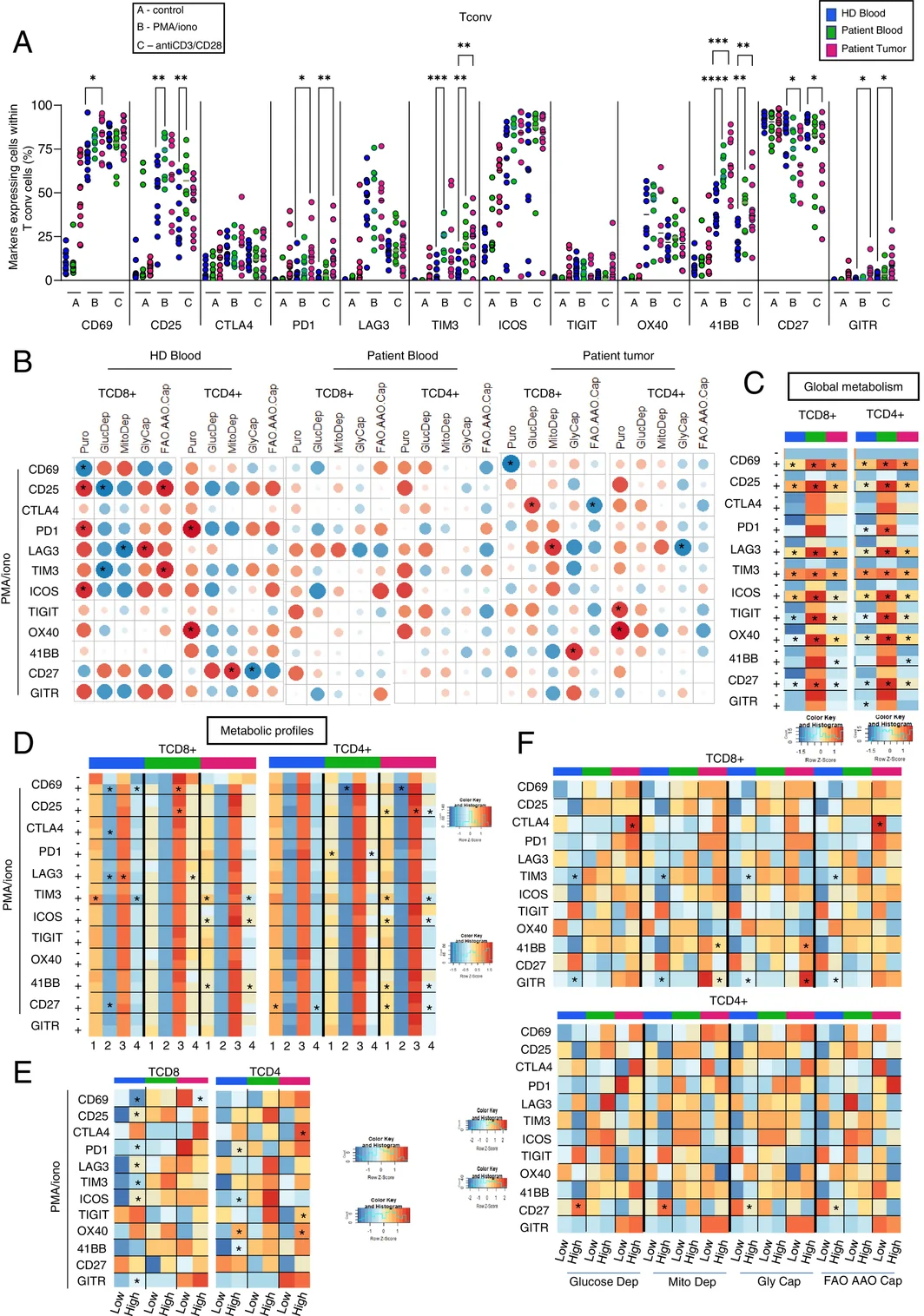
Distinct remodelling of expression profiles of activation markers and immune checkpoints by T cell subsets upon stimulation is linked with differential metabolic reprogramming in melanoma. PBMC from HDs and melanoma patients as well as CD45^+^ tumour‐infiltrating cells from patients were stimulated or not with either PMA/iono or anti‐CD3/CD28 antibodies, and the metabolism of T cell subsets was depicted using the SCENITH method along with their phenotypic features by flow cytometry. (A) Profile of expression of activation markers and immune checkpoints in total T (Tconv) cells in the different conditions of stimulation for HD blood (blue, *n* = 8–11), patient blood (green, *n* = 8–10) and tumour (pink, *n* = 8–13). *p* values were calculated using non‐parametric two‐way ANOVA or a mixed effect model followed by Tukey's multiple comparison post‐tests. Only significant statistics are displayed on graphs. *p ≤ 0.05, **p ≤ 0.01, ***p ≤ 0.001, ****p ≤ 0.0001. (B) Correlation matrix between the metabolic parameters of T cell subsets (global metabolism ‘puro’ as well as glycolytic/mitochondrial dependencies and capacities [GlucDep, MitoDep, GlyCap and FAO AAO Cap, respectively]) and their expression of activation markers and ICPs under PMA/iono stimulation for HD blood (blue, *n* = 10–11), patient blood (green, *n* = 9–10) or tumour (pink, *n* = 11–13). *p* values were calculated using a Spearman test followed by Holm correction. Only significant statistics are displayed on graphs. *p ≤ 0.01. (C, D) Heat maps displaying the (C) global metabolism and (D) glycolytic or mitochondrial dependencies/capacities of T cell subsets, stratified according to their expression (+) or not (−) of activation markers and ICP upon PMA/iono stimulation for HD blood (blue, *n* = 9–10), patient blood (green, *n* = 8–9) or tumour (pink, *n* = 8–12). (D) Metabolic profiles were enumerated as follows: (1) glucose dependency, (2) mitochondrial dependency, (3) glycolytic capacity, and (4) FAO/AAO capacity. All significant statistics are displayed on corresponding graphs in Figures S10–S13. (E, F) Heat maps displaying the expression levels of activation markers and ICPs on T cell subsets stratified according to their low or high (E) global metabolism and (F) glycolytic or mitochondrial dependencies/capacities upon PMA/iono stimulation, for HD blood (blue, *n* = 10–11), patient blood (green, *n* = 8–9) or tumour (pink, *n* = 11). Groups were separated using the median of the metabolic parameter in the corresponding T cell subset.

To assess a potential link between phenotype and metabolism, we performed correlation matrices between proportions of activation markers/ICP‐expressing cells and metabolic activity and profiles of T cell subsets upon PMA/iono (Figure 5B) or anti‐CD3/CD28 (Figure S9A) stimulation. In HDs, some markers were positively linked with high metabolism (e.g., ‘puro’ MFI) for CD8^+^ T cells (CD25, PD1, ICOS) and CD4^+^ T cells (PD1, OX40), whereas no clear link between phenotype and global metabolism was noticed for circulating T cells of patients, and other ICP were linked with high metabolism in tumour‐infiltrating CD4^+^ T cells (TIGIT, OX40) (Figure 5B). Strikingly, when investigating the metabolic pathways sustaining the phenotypic features of T cells upon stimulation, we observed in HD blood a metabolic pathway‐dependent orientation for the expression of some ICPs, which was not the case for circulating and tumour‐infiltrating T cells of patients, especially upon PMA/iono stimulation (Figure 5B). Indeed, in HD blood, CD25 and TIM3 expression by CD8^+^ T cells were positively associated with FAO/AAO capacity, whereas LAG3 was positively associated with glycolytic capacity, and CD27 expression by CD4^+^ T cells positively associated with mitochondrial dependency (Figure 5B); this underlines metabolic pathway‐specific‐triggering of ICP expression. This suggests that in HDs, ICP profile is globally dependent on both glycolytic and mitochondrial capacities, but such link was strongly perturbed or dampened in patients, suggesting usage of other metabolic pathways to sustain phenotypic modulations upon stimulation. This was illustrated in tumour‐infiltrating CD8^+^ T cells by the positive association between CTLA4 expression and glucose dependency, the positive correlation between LAG3 expression and mitochondrial dependency, and the positive link between 41BB expression and glycolytic capacity (Figure 5B). Interestingly, key ICPs such as LAG3 or ICOS were differentially metabolically‐driven in patients and HDs, underlining that the skewed metabolic reprogramming of T cells in patients could trigger perturbated ICP profile.

To get another view, we compared the metabolism of marker‐expressing (+) and marker‐negative (−) T cells upon PMA/iono (Figure 5C,D; Figures S10 and S11) or anti‐CD3/CD28 stimulation (Figures S11B,C, S12, S13). Heat maps based on the global metabolism level or percentages of dependencies/capacities overviewed the comparisons for CD8^+^ and CD4^+^ subsets (Figure 5C and Figure S11B,C). Individual graphs of comparison for each marker provided information on statistical significance for CD8^+^ T cells upon PMA/iono or anti‐CD3/CD28 (Figures S10 and S12) stimulation, and for CD4^+^ T cells upon PMA/iono or anti‐CD3/CD28 (Figures S11 and S13) stimulation. For most activation markers and ICPs, we observed a significantly higher global level of metabolism in marker‐expressing versus marker‐negative CD4^+^ and CD8^+^ T cells in all groups (Figure 5C and Figure S9B). Yet, such a difference was not observed in tumour‐infiltrating T cells for PD1 on CD4^+^ T cells (for both stimulations), TIGIT on CD4^+^ T cells and CTLA4 on CD8^+^ T cells (upon anti‐CD3/CD28 stimulation). This suggests that phenotypic changes in T cells occurring after stimulation are sustained by an enhanced global metabolism, but some ICPs (especially PD1, CTLA4 and TIGIT) do not rely on metabolism for their expression in the melanoma microenvironment.

Regarding metabolic profiles, T cells from HDs (both subsets) globally preferentially relied on glycolysis over OXPHOS, whereas circulating and tumour‐infiltrating T cells from patients exhibited a dampened glycolysis dependency and favoured both glycolytic and FAO/AAO capacities upon PMA/iono (Figure 5D) and anti‐CD3/CD28 (Figure S9C) stimulation, with an enhanced glycolysis capacity for tumour‐infiltrating T cells. Specifically, in HD blood, significant differences of metabolic profiles according to phenotypic features were observed for CD69‐, CTLA4‐, LAG3‐ and TIM3‐expressing CD8^+^ T cells upon PMA/iono stimulation (Figure 5D) and for CD69‐, CD25‐, PD1‐, LAG3‐, TIM3‐, TIGIT‐, OX40‐ and CD27‐expressing CD8^+^ T cells upon anti‐CD3/CD28 stimulation (Figure S9C). Such differences were observed for CD4^+^ T cells for most studied markers upon anti‐CD3/CD28 stimulation (Figure S9C). Globally, ICP‐positive cells exhibited a dampened mitochondrial dependency concomitant to an enhanced glycolytic capacity, or a higher glucose dependency concurrently to a decreased FAO/AAO capacity compared to negative cells. Such differences in metabolic profiles according to ICP expression were abrogated in circulating CD8^+^ and CD4^+^ T cells from patients, except for the activation markers CD69‐ and CD25‐, and for PD1‐expressing CD4^+^ T cells upon PMA/iono stimulation (Figure 5D). For patient tumour, significant differences of metabolic profiles according to phenotypic features were observed for TIM3 and ICOS on both CD8^+^ and CD4^+^ T cells upon PMA/iono stimulation, CD69 and CD25 on CD4^+^ T cells upon both stimulations, as well as LAG3, TIM3 and OX40 on both T cell subsets upon anti‐CD3/CD28 stimulation (Figure 5D and Figure S9C). These observations suggest that the phenotypic features of T cells after stimulation could be supported by specific metabolic profiles, which may differ between HDs and melanoma patients.

Conversely, we analysed the phenotypic features of T cells according to low or high global metabolism (Figure 5E and Figure S9D), as well as low or high metabolic capacities/dependencies (Figure 5F and Figure S9E). In HD blood, T cells displaying a high metabolic activity upon PMA/iono stimulation exhibited an enhanced expression of LAG3, ICOS, CD25, and GITR for CD8^+^ T cells, and PD1 for CD4^+^ T cells (Figure 5E). Upon anti‐CD3/CD28 stimulation, CD69 and TIM3 were preferentially found on metabolically active CD4^+^ T cells (Figure S9D). In patient blood, upon PMA/iono stimulation, metabolically active CD4 T cells exhibited higher levels of ICOS, while upon anti‐CD3/CD28 stimulation, metabolically active CD8 T cells exhibited lower levels of OX40. In patient tumour, T cells with a higher metabolism displayed higher expression of CD25 upon anti‐CD3/CD28 stimulation, in addition to enhanced expression of CTLA4, TIGIT, and OX40 for CD4 T cells upon PMA/iono stimulation (Figure 5E and Figure S9D). The relationship between high global metabolism and activation marker or ICP expression was different according to the stimulus (PMA/iono versus anti‐CD3/CD28), T cell subsets and the analysed sample group.

Interestingly, comparison of phenotypic features according to metabolic profiles revealed key differences especially for tumour‐infiltrating T cells (Figure 5F and Figure S9E). In patient tumour, CD8^+^ T cells with a high glucose dependency and low FAO/AAO capacity displayed higher levels of CTLA4 and TIGIT upon PMA/iono (Figure 5F). Tumour‐infiltrating CD8^+^ T cells with a low mitochondrial dependency and high glycolytic capacity exhibited higher levels of GITR and 4‐1BB than cells with an opposite metabolic profile upon PMA/iono stimulation (Figure 5F). Moreover, TIM3 and LAG3 expression by intratumoural CD8 T cells was linked to a high mitochondrial dependency. These observations highlight a link between metabolic reprogramming and ICP profiles of T cells upon stimulation. The distinct remodelling of expression profiles of activation markers and ICP by T cell subsets upon stimulation is driven by differential metabolic reprogramming.

### 3.7 Metabolic Profiles of T Cells Are Linked With Their Functional Orientation Upon Stimulation

To explore whether the metabolism can dictate functional orientation and investigate the potential correlations between T cell metabolic patterns and functional features, we assessed simultaneously T cell metabolism using the SCENITH method and cytokine production within each subset through intracellular cytokine staining upon stimulation (Figure 6 and Figures S14–S17). In patient blood and tumour, we observed increased proportions of IFN‐γ and TNF‐α secreting cells compared to HDs, but in association with IL13‐ (for both circulating and tumour‐infiltrating CD4^+^ and CD8^+^ T cell subsets) and IL10‐ (for circulating CD4^+^ T cells) producing cells (Figure 6A,B and Figure S14A). Even though not significant, we also observed an increase in tumour‐infiltrating IL17‐producing T cells. These results suggest a functional skewing of circulating and tumour‐infiltrating CD4^+^ and CD8^+^ T cells toward Th2/Treg profiles in melanoma patients.

**FIGURE 6:**
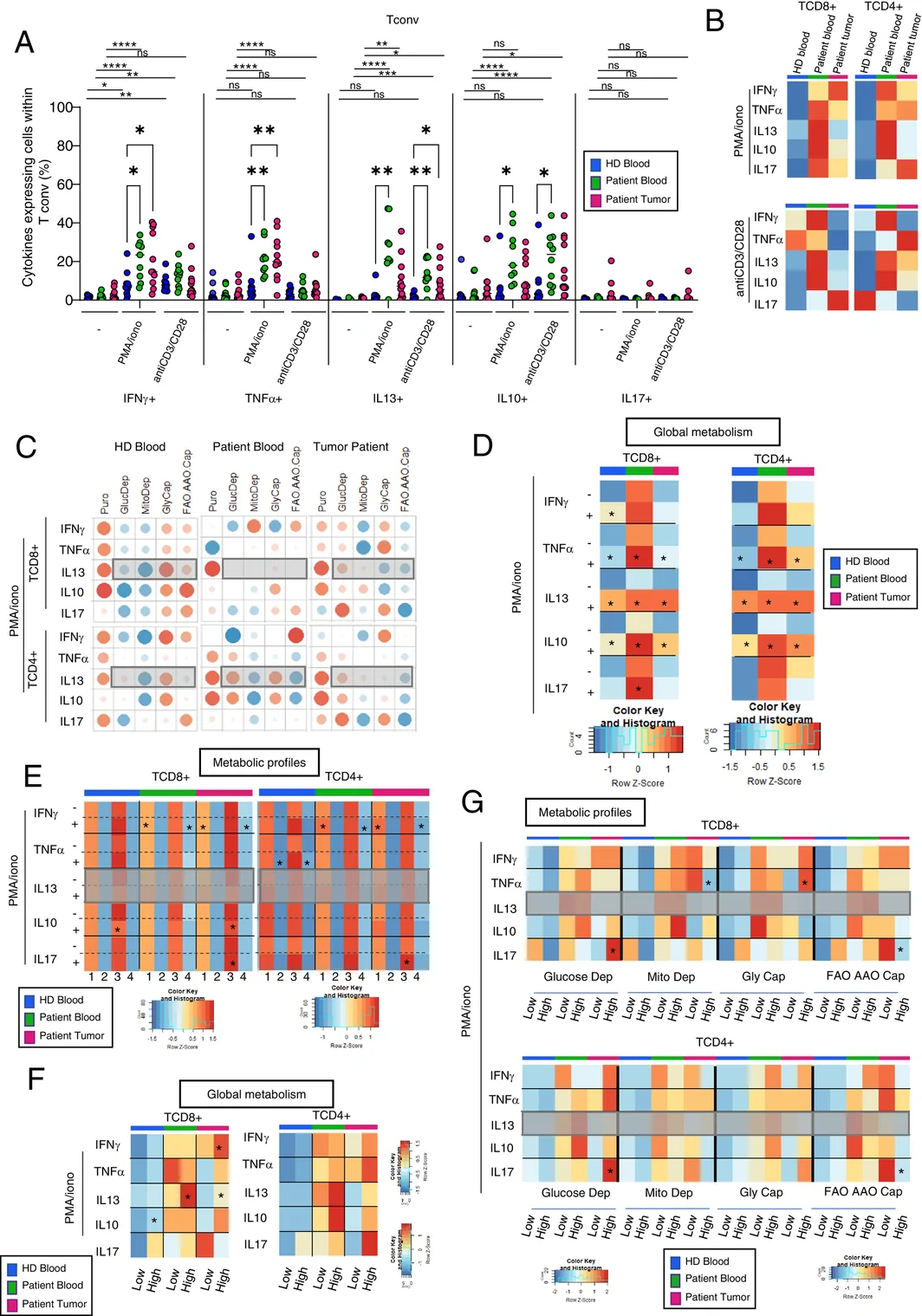
Metabolic profiles of T cell subsets are linked with their functional orientation upon stimulation. PBMC from HDs and melanoma patients as well as CD45^+^ tumour‐infiltrating cells from patients were stimulated or not with either PMA/iono or anti‐CD3/CD28 antibodies, and their metabolism was determined using the SCENITH method simultaneously to intracellular cytokine measurements by flow cytometry. (A) Profile of cytokine expression by total T cells (Tconv) in the different conditions of stimulation for HD blood (blue, *n* = 8–10), patient blood (green, *n* = 8–10) and tumour (pink, *n* = 11–13). (B) Heat map displaying proportions of cytokine‐producing CD8^+^ and CD4^+^ T cells from HD blood, patient blood and patient tumour upon PMA/iono or anti‐CD3/CD28 stimulation. Each square represents the median of each subgroup. (C) Correlation matrix between the metabolic parameters of T cell subsets (global metabolism ‘puro’ as well as glycolytic/mitochondrial dependencies and capacities [GlucDep, MitoDep, GlyCap and FAO AAO Cap, respectively]) and their cytokine expression upon PMA/iono stimulation for HD blood (blue, *n* = 10–11), patient blood (green, *n* = 9–10) or tumour (pink, *n* = 11–12). *p* values were calculated using a Spearman test followed by Holm correction. Only significant statistics are displayed on graphs. (D, E) Heat maps displaying (D) the global metabolism and (E) the glycolytic or mitochondrial dependencies/capacities of T cells, stratified according to their expression (+) or not (−) of cytokines upon PMA/iono stimulation for HD blood (blue, *n* = 10–11), patient blood (green, *n* = 8–10) or tumour (pink, *n* = 12–13). Metabolic profiles were enumerated as follows: (1) glucose dependency, (2) mitochondrial dependency, (3) glycolytic capacity, and (4) FAO/AAO capacity All significant statistics are displayed on corresponding graphs in Figures S19 and S20. (F, G) Heat maps displaying the proportions of cytokine‐expressing T cell subsets upon PMA/iono stimulation stratified according to their low or high (F) global metabolism and (G) glycolytic or mitochondrial dependencies/capacities (GlucDep, MitoDep, GlyCap and FAO AAO Cap) for HD blood (blue, *n* = 10–11), patient blood (green, *n* = 8–10) or tumour (pink, *n* = 12–13). Groups were separated using the median metabolic parameter in the corresponding T cell subset. *P ≤ 0.05, **P ≤ 0.01.

We further assessed the multifunctionality of the cells through correlation analyses between these functional profiles (Figure S14B). Correlations between proportions of (relevant) cytokine‐expressing cells within each group indicated that some modulations were linked together. In HDs, we observed a positive correlation between IFN‐γ and TNF‐α secreting cells, especially for CD8^+^ T cells whatever the stimulus. This correlation, compatible with a Th1 profile, is abrogated in patient blood and remains only for tumour‐infiltrating CD8^+^ T cells upon PMA/iono stimulation (Figure S14B). In patient blood and tumour, a strong positive link occurred between IL‐13 and IL‐10 secreting CD4^+^ T cells upon PMA/iono stimulation (Figure S14B), suggesting a Treg/Th2 profile. In patients, the absence of link between IFN‐γ producing cells and other cytokine‐producing cells suggested the presence of distinct functional subsets (Figure S14B).

To depict the impact of metabolism on T‐cell functional orientation, we performed correlation matrices between proportions of (relevant) cytokine‐secreting cells and metabolic activity and profiles within the corresponding cell population upon both PMA/iono (Figure 6C) and anti‐CD3/CD28 (Figure S15A) stimulation. No statistically significant correlations were found for CD4^+^ and CD8^+^ T cells upon PMA/iono stimulation whatever the considered cytokine or sample group (Figure 6C). In HDs, proportions of IFN‐γ‐secreting T cells were positively associated with high levels of global metabolism (‘puro’) upon anti‐CD3/CD28 stimulation (Figure S15A). The same observation was done for IL‐13‐secreting CD4^+^ and CD8^+^ T cells and IL‐10‐secreting CD8^+^ T cells (Figure S15A). These data were not observed in T cell subsets from patient blood or tumour, except for the proportions of circulating CD4^+^ T cells secreting IFN‐γ, which were positively associated with high levels of global metabolism (‘puro’) upon anti‐CD3/CD28 stimulation (Figure S15A). This suggested an altered response to anti‐CD3/CD28 stimulation in patients that is more pronounced in tumour‐infiltrating T cell subsets. Regarding metabolic profiles, no significant correlations were found for T cell subsets from HD blood whatever the stimulus (Figure 6C and Figure S15A). Data were not interpretable for IL‐13, since the addition of 2‐DG altered IL‐13 staining. Overall, the absence of clear correlation between cytokine‐producing cells and metabolic profiles suggested that cytokine expression is flexible according to the pathway and may depend on other metabolic pathways found in TME for tumour‐infiltrating T cell subsets.

To get another view, we compared the metabolism of cytokine‐secreting (+) and non‐secreting (−) T cells upon PMA/iono (Figure 6D,E) or anti‐CD3/CD28 (Figure S15B,C) stimulation. Individual graphs of comparison for each cytokine provided information on statistical significance for CD8^+^ T cells (Figure S16) and CD4^+^ T cells (Figure S17) upon PMA/iono or anti‐CD3/CD28 stimulation. For most cytokines, we observed a higher global level of metabolism in cytokine‐expressing versus cytokine‐negative CD4^+^ and CD8^+^ T cells in all groups (Figure 6D and Figure S15B). Yet, such difference was not observed upon PMA/iono stimulation for IFN‐γ secreting CD4^+^ T cells whatever the sample group and for IFN‐γ secreting CD8^+^ T cells in blood and tumour of patients (Figure 6D). For IL‐17 secretion, a lower global level of metabolism was observed for tumour‐infiltrating IL17‐secreting versus IL‐17‐negative CD8^+^ T cells upon CD3/CD28 stimulation (Figure S15B). Overall, this suggest that functional changes in T cells occurring upon CD3/CD28 stimulation are most of the time (except for IL‐17) sustained by an enhanced global metabolism. However, when the TCR triggering was bypassed (i.e., with PMA/iono stimulation), IFN‐γ by T cell subsets may not require an increased metabolism for their expression in the melanoma TME.

Regarding metabolic profiles, T cells (both subsets) globally preferentially relied on glycolysis over OXPHOS for cytokine secretion upon both stimulation (Figure 6E and Figure S15B). IFN‐γ secreting and non‐secreting CD8^+^ and CD4^+^ T cells upon PMA/iono stimulation had a similar metabolic balance in HDs, whereas these cells exhibited an enhanced glycolytic dependency associated with a decreased FAO/AAO capacity compared to non‐expressing cells in blood and tumour of patients (Figure 6E). In patient tumour, the glycolytic capacity dampened in IL10‐secreting CD8^+^ T cells, while this parameter increased in IL17‐secreting CD8^+^ and CD4^+^ T cells compared to non‐expressing cells (Figure 6E). These observations suggested that the functional features of T cells could be differentially supported by metabolic activity in melanoma context, and underlined the complexity of the metabolic regulation of T‐cell functional orientation.

Conversely, we analysed the functional features of T cells according to their low or high global metabolism (Figure 6F and Figure S15D), as well as their low or high metabolic capacities/dependencies (Figure 6G and Figure S15E). In patient blood, metabolically active CD8^+^ T cells exhibited higher levels of IL‐13 upon PMA/iono stimulation (Figure 6F), while metabolically active CD4^+^ T cells expressed higher levels of IFN‐γ upon anti‐CD3/CD28 stimulation, as observed in HD blood (Figure S15D). In patient tumour, CD8^+^ T cells with a high metabolic activity upon PMA/iono stimulation displayed enhanced proportions of IFN‐γ and IL‐13 producing cells (Figure 6F), whereas IL‐17‐producing cells are more abundant within high metabolically active CD4^+^ T cells following anti‐CD3/CD28 stimulation (Figure S15D).

Interestingly, comparison of functional features according to metabolic profiles revealed associations between specific metabolic pathways and functional orientation of T cells (Figure 6G and Figure S15E). While in HD blood, IFN‐γ and TNF‐α productions upon PMA/iono stimulation were not clearly associated with a metabolic profile, IFN‐γ and TNF‐α productions by CD4^+^ T cells upon CD3/CD28 stimulation were associated with a low glycolysis dependency concomitant to a high FAO/AAO capacity (Figure S15E). In contrast, all these observations were not found for T cell subsets of patient blood or tumour. In patient blood, IL‐17‐producing CD8^+^ T cells upon CD3/CD28 stimulation were associated with low mitochondrial dependency and high glycolysis capacity (Figure S15E). In tumour upon PMA/iono stimulation, IL‐17 producing CD8^+^ T cells and CD4^+^ T cells were associated with a high glucose dependency and a low FAO/AAO capacity (Figure 6G).

To further investigate whether the metabolism can influence the functional status of T cells, we explored simultaneously, the metabolic profile of T cell subsets and their pattern of secretion of a large panel of cytokines involved in immunity, tolerance or inflammation (Figure 7 and Figure S18). In the absence of stimulation, we noticed a higher level of TGF‐β in culture from patient blood compared to HD blood (Figure 7A,B). Stimulation triggered secretion of a large panel of cytokines, with quantitative variations between the groups. In patient blood, we observed significant higher levels of IL‐2, IL‐9 and GM‐CSF under PMA/iono stimulation, and of IFN‐γ, IL‐9, IL‐21, TGF‐β and GM‐CSF upon anti‐CD3/CD28 stimulation compared to HD blood, while tumour‐infiltrating T cells displayed higher level of IL‐21 upon anti‐CD3/CD28 stimulation compared to HD blood (Figure 7A,B and Figure S18A). Yet, both patient blood and tumour exhibited dampened level of IL‐1β upon stimulation compared to HD blood (Figure 7A,B). In order to explore the link between metabolic parameters and the secretome of T cells, we then performed correlations between these variables (Figure 7C and Figure S18B,C). In HDs, secretion levels of several cytokines (such as IL‐5, IL‐13, GM‐CSF) upon PMA/iono stimulation positively correlated with the global metabolism (‘puro’) of CD8^+^ and CD4^+^ T cells, whereas this pattern was totally abrogated for circulating and tumour‐infiltrating T cells of patients (Figure 7C and Figure S18B). For metabolic profiles, most of correlations did not reached significance, but interesting patterns could be noticed. In HDs upon PMA/iono stimulation, we observed that some mediators were exclusively dependent on glycolytic capacity of T cells (such as IL‐1β) while other cytokines relied greater on FAO/AAO capacity of T cells (such as TGF‐β, IL‐23) and others tended to be linked to both capacities (such as IL‐13) (Figure 7C, HD blood). In patient blood, most of cytokine secretions did not rely on glycolytic or mitochondrial pathways whatever the stimulation used, except for IL‐10 and TGF‐β who tended to be associated with mitochondrial dependency of CD8^+^ and CD4^+^ T cells respectively upon PMA/iono stimulation. Strikingly, upon anti‐CD3/CD28 stimulation, a strong correlation was observed between the production of TGF‐β and the glucose dependency of CD8^+^ T cells (Figure S18C). Regarding tumour‐infiltrating T cells, IFN‐γ levels tended to be associated with the glycolytic capacity of T cells upon PMA/iono stimulation (Figure 7C), whereas IL‐10 secretion tended to be linked to the glucose and mitochondrial dependencies of T cells upon anti‐CD3/CD28 stimulation (Figure S18C). Altogether, these observations suggest a link between the metabolic status of T cell subsets and the composition of their secretome in accordance with their microenvironment, but revealed also that other metabolic pathways than glycolysis and OXPHOS could be involved in the regulation of T cell fate, especially in melanoma patients.

**FIGURE 7:**
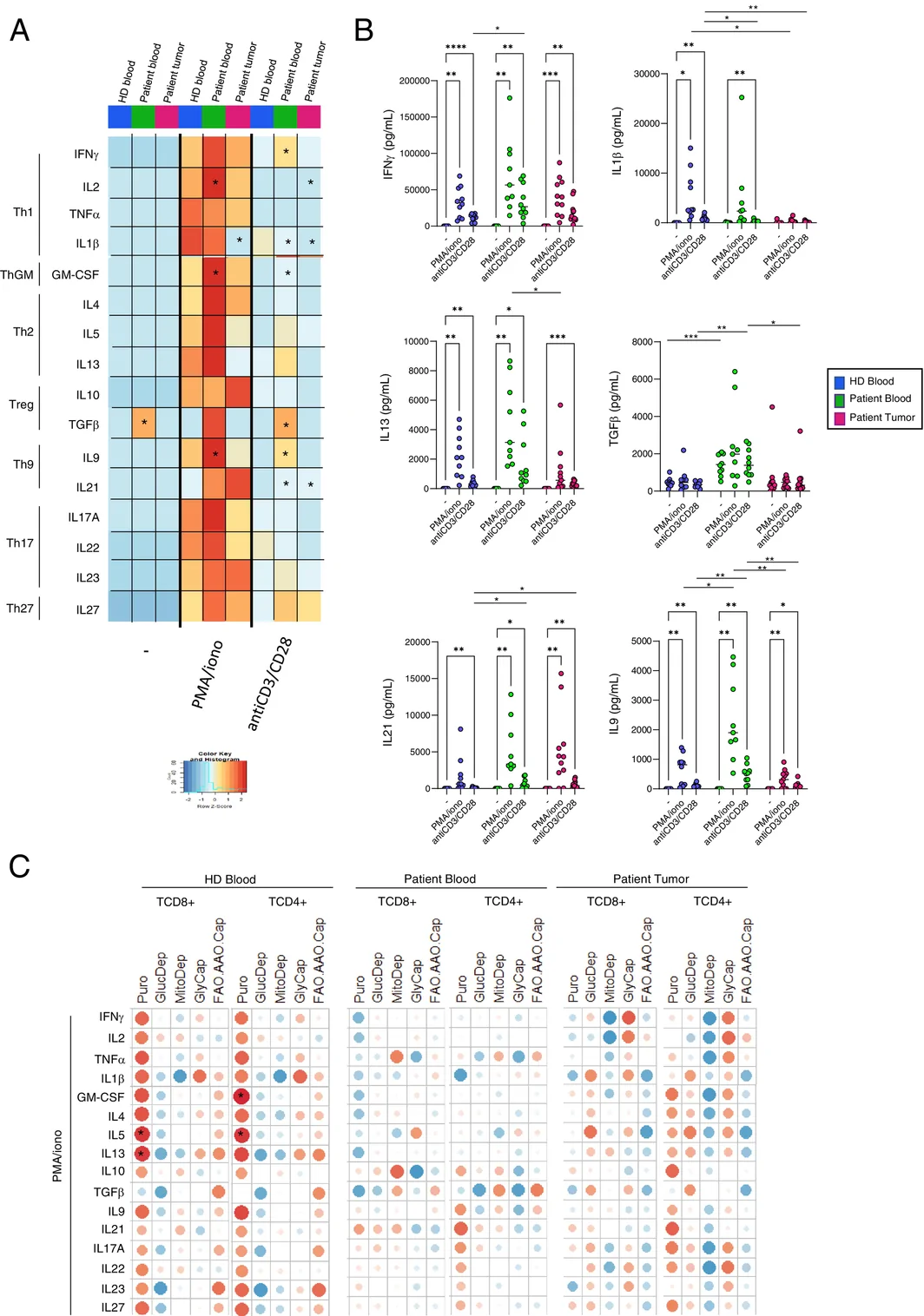
Link between the metabolic status of T cell subsets and the composition of their secretome under stimulation. PBMC from HDs and melanoma patients as well as CD45^+^ tumour‐infiltrating cells from patients were stimulated or not with either PMA/iono or anti‐CD3/CD28 antibodies, and their metabolism was depicted using the SCENITH method simultaneously to measurement of their secretome by Luminex. (A) Heat map displaying cytokine secretion levels from HD blood, patient blood and patient tumour in control conditions or upon PMA/iono or anti‐CD3/CD28 stimulation. Each square represents the median of each subgroup. (B) Comparative levels of secreted cytokines from HD blood (blue, *n* = 10), patient blood (green, *n* = 10) or tumour (pink, *n* = 11–13) upon stimulation (PMA/iono or anti‐CD3/CD28) or not. Bars indicate median. *p* values were calculated using non‐parametric two‐way ANOVA or a mixed effect model followed by Tukey's multiple comparison post‐tests. Only significant statistics are displayed on graphs. *p ≤ 0.05, **p ≤ 0.01, ***p ≤ 0.001, ****p ≤ 0.0001. (C) Correlation matrix between the metabolic parameters of T cell subsets (global metabolism (MFI of puromycin ‘puro’) and glycolytic or mitochondrial dependencies/capacities [GlucDep, MitoDep, GlyCap and FAO AAO Cap, respectively]) and cytokine secretion levels from HD blood (blue, *n* = 10), patient blood (green, *n* = 10) or tumour (pink, *n* = 12–13) upon PMA/iono stimulation. *p* values were calculated using a Spearman test followed by Holm correction. Only significant statistics are displayed on graphs. *p ≤ 0.01.

### 3.8 Exploration of T Cell Metabolism by LC–MS

To get an overview of other metabolic pathways potentially involved in the T‐cell subset metabolic deregulation, we performed LC–MS analysis on purified CD4^+^ and CD8^+^ T cells from HD, patient blood and tumour to widely assess general metabolites (*n* = 100) and lipids (*n* = 700) [29, 30] (Figure S19). Cell subsets were sorted using the Tyto sorter to preserve their metabolic features [28]. For CD4^+^ T cells, PLS‐DA analyses based on quantification of general metabolites or lipids revealed different patterns between the groups, allowing to distinguish patients from HDs (Figure 8A). We then performed *limma* analysis to determine the top 12 significant differences selected based on *p* value rankings (Tables S3 and S4). Overall, we highlighted major changes in amounts of amino acids and specific subclasses of lipids in patient's tumour (Figure 8B), as well as patient's blood (Figure S20A) compared to HDs. While adenine and 5‐Thymidylic acid were upregulated in circulating CD4^+^ T cells and kynurenine elevated in tumour‐infiltrating CD4^+^ T cells, many amino acids were dampened in both blood and tumour of patients such as histidine, L‐threonine, glutamine and malic acid (Figure 8B and Figure S20A). Regarding lipids, while sterols especially CE(16:2) and ceramides were downregulated in circulating CD4^+^ T cells from patients (Figure S20A), many lipids strongly raised in CD4^+^ T cells from blood (especially glycerophosphoethanolamine and glycerophosphates) and tumour of patients, where the sterol cholesterol (CE(20:2)) and the ceramide dhCer(d18:0/22:0) exhibited log2 fold changes greater than 10. Using MetaboAnalyst, we depicted the metabolic pathways affected in tumour (Figure 8C) and blood (Figure S20B), and then mapped the top 12 shifts in general metabolites within tumour‐infiltrating (Figure 8D) and circulating (Figure S20C) CD4^+^ T cells. Notably, carbohydrate metabolism (blue pathway), amino acid metabolism (yellow pathway) as well as nucleotide metabolism (pink pathway) were deregulated in CD4^+^ T cells from both blood and tumour of melanoma patients compared to HDs, impacting pyruvate, malate and key amino acids such as glutamine, cysteine, threonine and alanine. In addition, major lipid classes such as glycerophospholipids, sphingolipids and sterols were impacted in both tumour‐infiltrating (Figure 8E) and circulating (Figure S20D) CD4^+^ T cells from patients. Regarding CD8^+^ T cells, PLS‐DA analyses based on quantification of general metabolites or lipids also revealed different patterns between the groups, allowing to distinguish patients from HDs (Figure 9A). Within the top 12 significant differences in general metabolites between patients and HDs, nucleotides (such as guanosine) were elevated in CD8^+^ T cells from both blood and tumour whereas amino acid abundance (especially taurine, glutamic acid, L‐cysteine and above all citrulline) consistently decreased across both tumour (Figure 9B) and blood CD8^+^ T cells (Figure S21A). Regarding lipids, the glycerolipid DG(16:0_16:0) strongly raised in CD8^+^ T cells from patients, with a log2 fold change greater than 10 for both tumour and blood (Figure 9B and Figure S21A). This was accompanied by increased abundance of glycerophosphocholines, diradylglycerols and glycerophosphoglycerols as well as dampened amounts of glycerophosphoethanolamines, ceramides and glycosphingolipids. MetaboAnalyst allowed to depict the metabolic pathways that contain the top 12 altered general metabolites in tumour (Figure 9C,D) and blood CD8^+^ T cells (Figure S21B,C). Amino acid as well as nucleotide metabolism were the most deregulated in CD8^+^ T cells from both blood and tumour of melanoma patients compared to HDs, with impact on key amino acid such as glutamate, cysteine and threonine. Furthermore, major lipid classes such as glycerolipids, glycerophospholipids, sphingolipids and sterols were impacted in both tumour‐infiltrating (Figure 9E) and circulating (Figure S21D) CD8^+^ T cells from patients.

**FIGURE 8:**
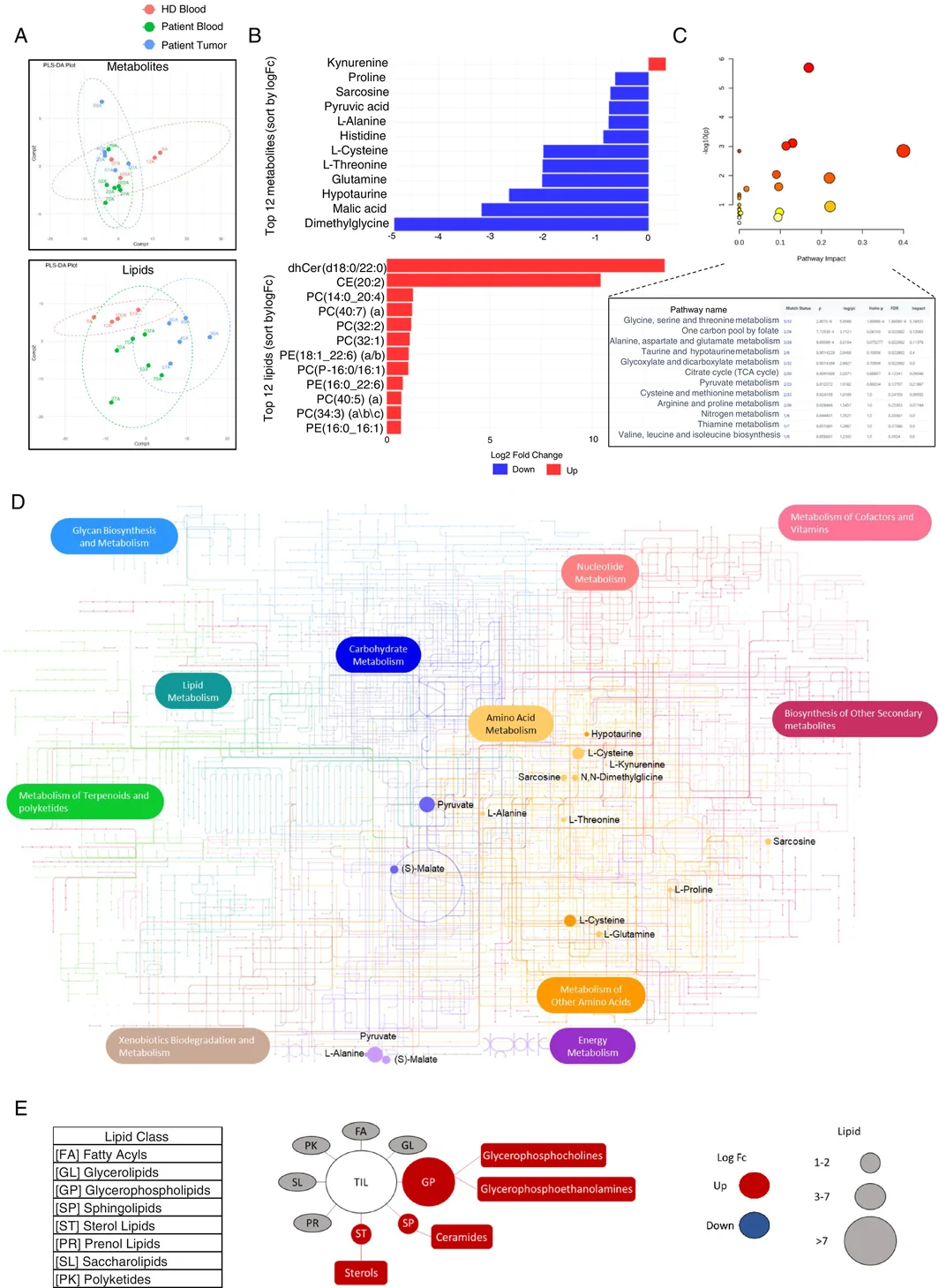
Metabolomic and lipidomic deregulations within tumour‐infiltrating CD4^+^ T cells. CD4^+^ T cells were isolated from PBMC of HDs and melanoma patients as well as tumour‐infiltrating cells from patients using Tyto sorter and conserved in methanol, H_2_O and formic acid solution at −80°C. Cell subset metabolomic and lipidomic was performed using LC–MS method. (A) PLS‐DA analysis for general metabolites (upper panel) and lipids (bottom panel) among HD blood (red, *n* = 4), patient blood (green, *n* = 6) and patient tumour (blue, *n* = 5). (B) Bar plots representing the top 12 metabolites and lipid changes sorted by log of Fold Change between HD blood (*n* = 4) and patient tumour (*n* = 5) after *limma* analysis (Table S3). Blue: Downregulation, red: Upregulation of the metabolites in patient's tumour compared to HD blood. (C, D) Enrichment's pathway and mapping of the top 12 changes in metabolites between HD blood (*n* = 4) and patient tumour (*n* = 5) using MetaboAnalyst software. (E) Representation of the top 12 changes in lipids among lipid classes and subclasses for differences between HD blood (*n* = 4) and patient tumour (*n* = 5).

**FIGURE 9:**
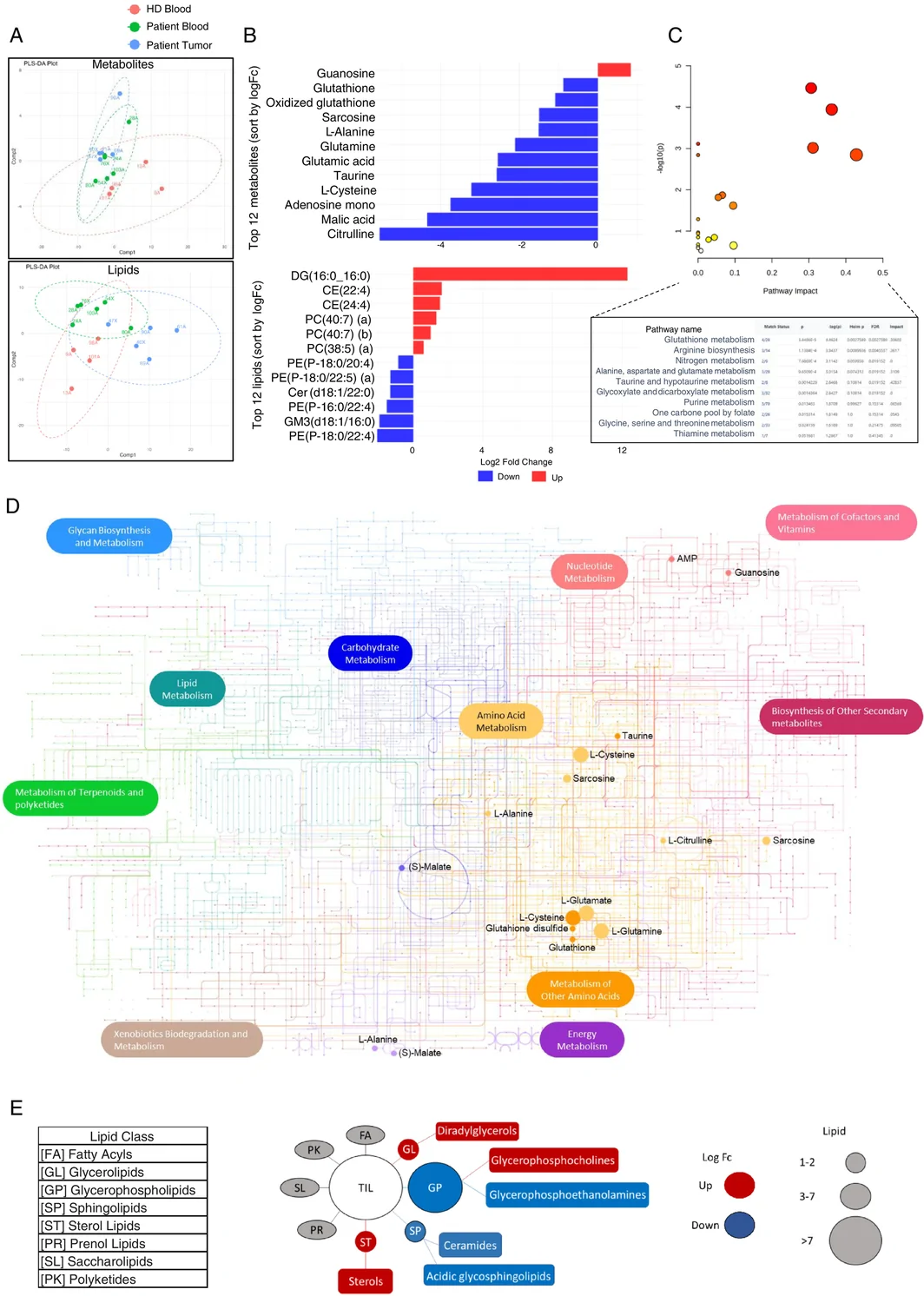
Metabolomic and lipidomic deregulations within tumour‐infiltrating CD8^+^T cells. CD8^+^ T cells were isolated from PBMC of HDs and melanoma patients as well as tumour‐infiltrating immune cells from patients using Tyto sorter and conserved in methanol, H_2_O and formic acid solution at −80°C. Cell subset metabolomic and lipidomic was performed using LC–MS method. (A) PLS‐DA analysis for general metabolites and lipid among HD blood (red, *n* = 4), patient blood (green, *n* = 6) and patient tumour (blue, *n* = 5). (B) Bar plots representing the top 12 metabolites and lipid changes sorted by log of Fold Change between HD Blood (*n* = 4) and patient tumour (*n* = 5) after *limma* analysis (Table S3). Blue: downregulation, red: upregulation of the metabolites in patient's tumour compared to HD blood. (C, D) Enrichment's pathway and mapping of the top 12 changes in metabolites between HD blood (*n* = 4) and patient tumour (*n* = 5) using MetaboAnalyst software. (E) Representation of the top 12 changes in lipids among lipids classes and subclasses for differences between HD blood (*n* = 4) and patient tumour (*n* = 5).

### 3.9 Clinical Relevance of Enzymes and Transporters Relative to the Perturbed Metabolites Observed in Tumour‐Infiltrating T Cells From Melanoma Patients

We further investigated the clinical relevance of pertinent enzymes and nutrient transporters involved in the synthesis, metabolisation or transport of the metabolites—whose level was previously highlighted to be either increased or dampened within tumour‐infiltrating T cells of melanoma patients—using RNA sequencing data from the Cancer Genome Atlas (TCGA) dataset (Skin Cutaneous Melanoma (SKCM) cohort, *n* = 461 patients) using the GEPIA2 server. We examined upstream and downstream enzymes as well as nutrient transporters relative to dhCer(d18:0/22:0), CE(20:2), DG(16:0/16:0), glutamine, malic acid and citrulline, whose levels were the most perturbated within tumour‐infiltrating CD4^+^ and/or CD8^+^ T cells of melanoma patients, and we combined them with the genes relative to metabolism and present within the top 100 genes that influence the most melanoma patient survival (Table S5). We first performed correlation analyses between the candidate genes and *Cd3e/Cd4/Cd8a* genes as well as the melanoma specific *MLANA/MITF* genes, and further assessed whether their expression level impacted patient OS. Within all the metabolism‐related candidate genes, we unravelled four enzymes and two transporters that displayed high correlation with *Cd3e/Cd4* (Figure 10A) or *Cd3e/Cd8a* (Figure 10B) expression, but were not linked with *MLANA/MITF*‐expressing tumour cells (Figure S22), and were associated with a favourable prognosis (Figure 10A,B and Table S6). *LIPA* and *DGKA* encoding enzymes metabolise CE(20:2) and DG(16:0/16:0) respectively, reducing the intracellular levels of these lipids. The other four genes encode two amino acid transporters (SLC38A1 and SLC7A7) and two enzymes (glutamine synthetase and GTP cyclohydrolase 1 [encoded by *GLUL* and *GCH1*, respectively]) involved in amino acid metabolism. The transporter SNAT1/SLC38A1, which imports glutamine and the glutamine synthetase, which catalyses glutamine synthesis, contributes to rise cellular glutamine levels. The amino acid transporter SLC7A7 imports arginine, which is further metabolised into citrulline thanks to GTP cyclohydrolase 1. The combination of this six‐metabolic gene‐based signature strongly dictates patient OS (Figure 10C). Overall, high expression of these key enzymes and transporters will reestablish accurate levels of metabolites that were found to be imbalanced in tumour‐infiltrating T cells from patients (Figure 10D). Altogether, we outlined a critical metabolic checkpoint‐based signature within tumour‐infiltrating T cells that shape T‐cell fitness and clinical outcome.

**FIGURE 10:**
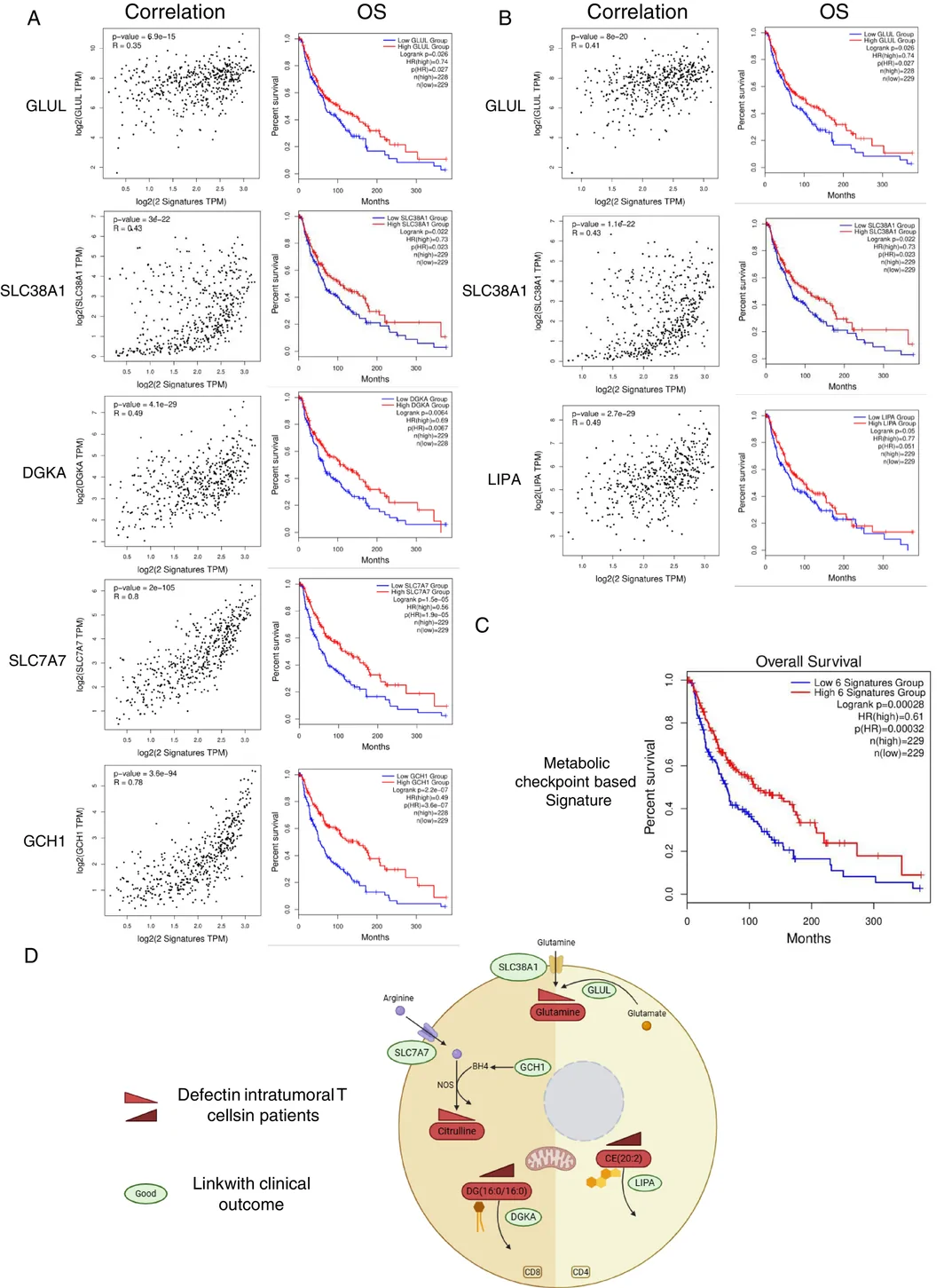
Clinical relevance of enzymes and transporters relative to the perturbed metabolites observed in tumour‐infiltrating T cells from melanoma patients. The clinical relevance of pertinent enzymes and nutrient transporters involved in the synthesis, metabolisation or transport of the metabolites previously highlighted to be either increased or dampened within tumour‐infiltrating T cells of melanoma patients was assessed using RNA sequencing data from the TCGA dataset (SKCM cohort, *n* = 461 patients) using the GEPIA2 server by performing correlation and survival analyses. (A, B) Correlation of the candidate gene expression with *Cd3e/Cd4* levels (A, left panels) or *Cd3e/Cd8a* levels (B, left panels), and overall survival (OS) of patients according to the expression level of the candidate gene (right panels) relative to the metabolites perturbated within CD4^+^ (A) or CD8^+^ (B) T cells. (C) Overall survival of melanoma patients according to the combined expression level of the six metabolic gene‐signature previously outlined (*LIPA*, *DGKA*, *GLUL*, *SLC38A1*, *SLC7A7*, *GCH1*). (D) Schematic overview positioning the key metabolite perturbations observed in tumour‐infiltrating T cells of melanoma patients along with the connected transporters and enzymes that impacted the clinical outcome of patients.

## 4 Discussion

Here, we unveiled major metabolic disturbances of T cell subsets in melanoma patients together with critical interplays between metabolic pathways and T‐cell features impacting clinical outcome. Melanoma evades the immune system by reprogramming T‐cell energetic metabolism. Our findings bring insights into the link between the two emerging hallmarks of cancer—metabolic reprogramming and immune evasion [32]. It further paves the way for appropriate tuning of metabolic pathways to rescue T cells from tumour hijacking, in order to restore antitumour responses and enhance the efficacy of current immunotherapies to achieve better clinical success.

Major disturbances in the metabolism of CD4^+^ and CD8^+^ T cells in melanoma were identified both at steady state and after activation. At steady state, an enhanced protein synthesis—reflecting a higher global metabolism—is measured in circulating CD8^+^ and CD4^+^ T cells and in tumour‐infiltrating CD8^+^ T cells of melanoma patients in comparison to circulating T cells of HDs. This increased global metabolism is associated with a metabolic reprogramming. In both blood and tumour of patients, CD8^+^ T cells exhibited a decreased mitochondrial dependency associated with an enhanced glycolytic capacity, while CD4^+^ T cells displayed a dampening in both dependencies accompanied by greater glycolytic and mitochondrial capacities. This alteration of T‐cell metabolic profiles by melanoma is stronger in the tumour compared to the blood and tends to be more important in advanced compared to early stage of the disease. Moreover, the expected enhanced global protein synthesis occurring upon activation was stronger in melanoma patients compared to HDs for both CD8^+^ and CD4^+^ T cells, especially for circulating T cells, and was associated with deregulation in metabolic pathway usage with a shift from high glycolysis dependency to FAO/AAO capacity. Deficient glycolytic metabolism may impair the anti‐tumour effector functions and favour Treg cells, since these cells mainly rely on FAO/AAO rather than glycolysis to survive under metabolically unfavourable conditions and exert their immunosuppressive effect [33, 34]. Strikingly, we also uncovered through LC–MS analysis that circulating and tumour‐infiltrating CD4^+^ and CD8^+^ T cells suffer from a dampening of amino acid pathways, a modulation of nucleotide metabolism as well as an increase in several lipid classes especially glycerophospholipids, sphingolipids, and sterols. As expected, we observed an increased kynurenine level within tumour‐infiltrating CD4^+^ T cells, which is consistent with the regulatory T cell profile previously shown in melanoma [16], validating our experimental approach and fitting with the Th2/Treg profile identified in tumour‐infiltrating CD4^+^ T cells. Taken together, these findings reveal that the melanoma microenvironment might evade immune surveillance by shaping the reprogramming of T cell energetic metabolism, thereby modulating their effector functions to support immunosuppression. Interestingly, we recently revealed major metabolic disturbances within circulating and tumour‐infiltrating DC subsets in melanoma patients, with functional and clinical impacts [27].

Our analyses at steady state show that both circulating CD4^+^ and CD8^+^ T cells from HD and patient blood used preferentially mitochondrial pathway over glycolysis. In tumour, CD4^+^ T cells used both pathways while CD8^+^ T cells rely preferentially on glycolysis. This underlines a potential in situ activation of T cells, confirmed by the high level of CD69. This is consistent with the knowledge that, at steady state, T cells depend on OXPHOS for energy production, and following activation, they often switch to glycolysis to match their energy demands for rapid proliferation and cytokine production [35]. Upon stimulation, the reduced glucose dependency and increased FAO/AAO capacity observed in patient CD4^+^ and CD8^+^ T cells suggest an abnormal switch to satisfy their metabolic needs by utilising alternative energy sources in the glucose‐deprived TME [36].

We demonstrated crucial links between T cell metabolism and patient clinical outcome. We identified bioenergetic profiles of T cells associated with early relapse or favourable outcome, depicting metabolic checkpoints crucial for favourable clinical outcome. Indeed, patients whose T cells exhibited a high glucose dependency or high glycolytic capacity underwent early relapse/short survival, whereas a high mitochondrial dependency or FAO/AAO capacity was associated with better prognosis. We also outlined a metabolic checkpoints‐based signature that critically shapes patient clinical outcome; this signature is composed of six genes encoding enzymes and metabolite transporters (*LIPA*, *DGKA*, *GLUL*, *SLC38A1*, *SLC7A7*, *GCH1*) closely connected to the imbalanced metabolites found within tumour‐infiltrating T cells. Tumour microenvironment is characterised by reduced glucose and amino acid levels [36]. Failure of T cells to adapt their metabolism in this metabolically challenging TME—by rewiring their metabolism from glycolysis to OXPHOS—may alter their functional capacity and lead to inefficient antitumour responses, and in consequence, to a bad prognosis. It is known that OXPHOS in tumour‐infiltrating CD3^+^ T cells frequently exhibits adaptation to tumour‐induced glucose deprivation in the TME, but it also induces cell dysfunction as glycolysis is essential for cell proliferation and cytokine production [21]. This is consistent with the finding that immunotherapy resistance in melanoma patients is predicted by a high OXPHOS profile in circulating and tumour‐infiltrating T cells [37]. Actually, besides just being associated with clinical outcome, metabolism reveals to be crucial for response to therapies. Many studies sustain a link between immune metabolism and response/resistance to ICB. In mice, PD1 blockade rescues exhausted T cells that display a defective metabolic profile by reversing glucose restriction, enhancing CD8^+^ T cell glucose influx and glycolysis, leading to restored effector function [38]. Bulk RNA sequencing analysis of sorted tumour‐infiltrating mouse T cells upon anti‐PD1 and/or ‐CTLA4 revealed differential gene enrichment, the combination of both revealing synergism of metabolic and effector genes [39]. In melanoma patients, a high OXPHOS CD8^+^ T cell subset has been associated with resistance to immunotherapy [37]. The glutamine transporter SLC1A5 has been identified as pivotal to distinguish responders and non‐responders to anti‐PD1 therapy in patients with HCC or melanoma [40]. Patients with low expression of SLC1A5 displayed a better survival, reduced glutamine metabolism, lower immunosuppressive cells, and better response to anti‐PD1. Altogether, metabolic checkpoints are crucial to dictate clinical outcome and response to therapies.

As the SCENITH method is a single‐cell flow cytometry‐based approach, we exploited the opportunity to combine metabolism assessment with phenotypic and functional measurements, including activation status, ICP profile and cytokine production. This represents a potent way to investigate the functional metabolic heterogeneity at a single‐cell level and to explore whether the observed dysfunctional cellular metabolism as well as the metabolic reprogramming of T cells in melanoma are linked with perturbated phenotypic profiles and skewed functional orientation. We showed here that it is feasible to combine the SCENITH method with phenotypic profiling and intracellular cytokine staining, except for IL‐13, that remains inexplicable yet. We uniquely unveiled critical interplays between metabolic pathways and T cell features and functions.

Our findings sustained a link between metabolic profiles and ICP expression within T cells. Indeed, T cells display distinct metabolic profiles depending on their activation status and ICP profile. This supports critical connections between T‐cell features and metabolic pattern. Distinct metabolic profiles were exhibited by ICP‐expressing and ICP‐non‐expressing T cells (e.g., CTLA4 or ICOS expression in tumour‐infiltrating CD4^+^ T cells associated with decreased OXPHOS and higher glycolysis capacity), and specific ICP profiles were connected to particular metabolic patterns (e.g., LAG3 and glucose dependency in circulating CD4^+^ T cells in melanoma patients). Some ICPs were linked with both glycolysis and OXPHOS, whereas others were preferentially associated with one of the pathways (e.g., 41BB in total T cells from patient blood and OXPHOS). This suggests that ICP expression could be flexible according to the pathway or supported by specific pathways. Identification of these ICPs supported by specific pathways could lead to new therapeutic interventions, combining ICB and metabolic intervention. In addition, upon stimulation, distinct remodelling of ICP expression profiles by T cells was associated with differential metabolic reprogramming in patients compared to HDs. For instance, upon PMA/iono stimulation, ICP profile of T cells from HDs is globally dependent on both glycolytic and mitochondrial capacities. In contrast, this link is strongly reduced in patient T cells, suggesting the involvement of other pathways that we further identified using LC–MS analysis (e.g., amino acid or lipid pathways).

Metabolic reprogramming and ICPs could influence each other in a bidirectional way. In the TME, nutrient deprivation, hypoxia, and accumulation of suppressive metabolites drive T cells into a dysfunctional state, which is associated with upregulation of inhibitory receptors. It is known that glucose deprivation, limited availability of certain amino acids (arginine, glutamine), and lipid accumulation cause impaired glycolysis and metabolic stress, enhancing T cell exhaustion markers [41]. Lipid accumulation within tumour‐infiltrating T cells is associated with increased inhibitory receptor expression (including PD‐1, TIM‐3, and LAG‐3) and metabolic exhaustion, contributing to reduced effector functions [42, 43, 44]. Conversely, ICP signalling following contact with their cognate ligand can also reshape T‐cell metabolic pathways [45]. Dysregulation of T‐cell metabolic pathways in melanoma could be a consequence of the upregulation of ICP on patient T cells, as we observed for PD1 and LAG3 expressed by circulating and tumour‐infiltrating CD8^+^ T cells. Indeed, signalling through ICPs affects functional T‐cell programs by impacting key metabolism regulators and promoting metabolic reprogramming [45, 46, 47, 48]. Activation of inhibitory ICPs—such as PD‐1 and CTLA‐4—directly suppresses key metabolic pathways in effector T cells, including glycolysis and the PI3K/Akt/mTOR pathway, reinforcing an immunosuppressive phenotype and dampening effector function in the TME [47]. T cells receiving signals through PD1 are unable to engage glycolysis, glutaminolysis, or amino acid metabolism, while increasing FAO. This is due to inhibition of glucose/glutamine uptake and hexokinase II (HK2, the rate‐limiting first step of glycolysis), as well as induction of lipolysis concomitantly to a decrease in lipid biosynthesis resulting from the inhibition of fatty acid synthase. Such metabolic reprogramming drives T‐cell to metabolic quiescence and exhaustion with limited biosynthetic activity and impaired function; this explains the high longevity of PD1^+^ T cells [49]. CTLA4 inhibits glycolysis without augmenting FAO, maintaining immune quiescence while favouring a switch of the T cell functional fate from effector T to Treg cell differentiation [45]. Stimulatory ICPs such as CD28, GITR, or 41BB that could be targeted by agonist drugs also regulate metabolic signatures by promoting metabolic reprogramming that enhances glycolysis, mitochondrial metabolism, and lipid utilisation. Activation of CD28 promotes the PI3K/Akt/mTORC1 pathway, increasing glycolysis and mitochondrial metabolism [47]. We found that all circulating and tumour‐infiltrating T cell subsets from patients strongly dampened their glycolytic dependency in response to anti‐CD3/CD28 stimulation in comparison to the same HD blood T cell subsets. This suggests reduced CD28 signalling pathway in melanoma patients. Nevertheless, both the negative or positive impact of inhibitory or stimulatory ICP triggering on antitumour responses is mediated through metabolic reprogramming of immune cells.

The metabolism of T cell subsets was also connected with their functional orientation, as illustrated by interconnections between specific cytokine secretion profiles and metabolic parameters (e.g., IFN‐γ expression after PMA/iono stimulation associated with glucose dependency and FAO/AAO capacity in patient blood CD4^+^ and CD8^+^ T cells). Metabolic pathways actively shape immune functions. Different metabolic pathways are associated with distinct cytokine profiles. Glycolysis is often linked to pro‐inflammatory cytokine production, whereas OXPHOS and FAO are generally associated with anti‐inflammatory or regulatory phenotypes [34]. Effector T cells rely heavily on aerobic glycolysis, Treg mainly on OXPHOS. Metabolic intermediates (such as succinate or citrate) can act as signalling molecules that directly regulate cytokine gene expression by regulating transcription factors [50]. Conversely, cytokines can reprogram cellular metabolism. Pro‐inflammatory cytokines (IFN‐γ, TNF‐α, IL‐1β) promote glycolysis and suppress mitochondrial respiration, while anti‐inflammatory cytokines (IL‐10, TGF‐β) favour mitochondrial metabolism and lipid oxidation, supporting immunoregulatory phenotypes [51, 52]. Thus, there is a bidirectional relationship between metabolism and cytokines. Metabolic changes in immune cells and altered cytokine secretion profile may be both a cause or consequence of the other one. Overall, crucial connections exist between metabolic disturbances of T cells and their phenotypic alteration as well as their functional polarisation. This highlights that metabolic reprogramming of circulating and tumour‐infiltrating effectors in melanoma patients could participate in immune hijacking and tumour escape. Yet, our data also underline the complexity of the metabolic regulation of T cell phenotypic and functional features, which may vary according to the cell subset, the nature of activation and the phenotypic/functional parameter.

Among metabolic perturbations of tumour‐infiltrating T cells, we found dampened amino acid levels and accumulation of specific lipids, pathways being key regulators of antitumour T‐cell immunity and may contribute to impaired T‐cell function and tumour evasion. Lipid metabolism shapes T‐cell activation, exhaustion, and antitumour immunity in the TME [44]. TCR signalling and T‐cell functionality are extremely sensitive to membrane lipid organisation and lipid metabolism. Alterations in lipid species such as cholesterol esters, dihydroceramides, and diacylglycerols are likely to have negative functional consequences on T‐cell activation [41, 42]. Accumulation of the cholesterol ester CE(20:2)—which sequestrates cholesterol—may limit TCR signalling by reducing membrane cholesterol; plasma membrane cholesterol content is essential to the formation of lipid rafts and TCR signalling, leading to T cell hypo‐activation and dysfunction [53]. Interestingly, inhibiting cholesterol esterification in T cells enhances plasma membrane cholesterol content, which boosts TCR clustering and finally CD8^+^ T cell effector function in melanoma models [54]. In the same way, the lysosomal acid lipase encoded by the *LIPA* gene—which level was related to a favourable clinical outcome—triggers the releases of free fatty acids and cholesterol from cholesteryl esters and triglycerides. Accumulation of ceramides in T cells, as illustrated here by dhCer(d18:0/22:0), is known to be associated with anergy, increased susceptibility to apoptosis, and unfavourable effector function, therefore impaired T cell fitness [55]. It has been shown that lipid accumulation in T‐cell infiltrating melanoma is associated with oxidative stress and dysfunction of cytotoxic T cells [56]. In addition, availability of diacylglycerol DG(16:0/16:0) modulates proximal TCR signalling, influencing T‐cell activation, proliferation, and production of cytokines [57]. Diacylglycerol kinase alpha, encoded by the *DGKA* gene, metabolises diacylglycerol (DAG) to phosphatidic acid, which attenuates TCR signalling by reducing DAG availability. In contrast to the link between *DGKA* level and better outcome that we observed, DGKA activity has been shown to contribute to T cell dysfunction, reduced cytokine production, impaired proliferation, and poor tumour infiltration [57]. DGKA inhibition has been shown to restore antitumour T cell responses and synergise with ICP blockade in melanoma [58]. Such observation emphasises the importance of examining enzyme activity and not only gene expression levels.

Within tumour‐infiltrating T cells, we also highlighted dampened amino acid levels and crucial connections between related transporter gene levels and clinical outcomes. Glutamine and citrulline, critical nutrients for activated T cells, supporting T‐cell proliferation, effector cytokine production and metabolic fitness, were found to be highly dampened within tumour‐infiltrating T cells. SLC38A1 transports neutral amino acids (including glutamine) into cells, which fuels metabolic programs in T cells enabling T‐cell activation, proliferation, and cytokine production. Interestingly, metabolic flux studies in melanoma link glutamine transporter expression to immune infiltration and anti‐tumour immunity; this supports the idea that glutamine transport (via SLC38A1) *correlates with lymphocyte infiltration and function* in melanoma [59]. SLC7A7 mediates cationic amino acid (especially arginine) transport, and participates in citrulline synthesis together with tetrahydrobiopterin (BH4) which synthesis depends on the GTP cyclohydrolase 1 (GCH1). In line with our findings, high SLC7A7 expression has been associated with immune infiltration signatures including CD4^+^ and CD8^+^ T cells in tumours [60], GCH1 has been shown to influence T‐cell activation threshold and antitumour activity [61].

Together, metabolic dysregulation observed in patient T cells likely contributes to T‐cell exhaustion and immune escape. We identified within tumour‐infiltrating T cells key metabolic processes that are critical for sustaining effective antitumour T‐cell responses. Targeting such metabolic checkpoints represents a promising strategy to enhance T cell fitness, control antitumour responses and improve responses to ICB in melanoma. Checkpoint inhibitors/agonists, by reversing metabolic reprogramming triggered by ICPs, restore T‐cell metabolic status and functional program that rescue exhausted tumour‐infiltrating lymphocytes [38, 62]. Direct metabolic interventions are currently developed to enhance antitumour immunity [63, 64], such as targeting nutrient availability, transporters (SLC families), enzymes to rewire glycolytic, mitochondrial, or lipid metabolism, with promising results in clinical trials [63, 65]. Metabolic interventions display strong potential in enhancing ICB efficacy. Several preclinical and clinical studies have identified metabolic targeting drugs with the potential to improve the efficacy of ICB therapy [65, 66, 67]. In accordance with our present findings, intratumoural methionine, glutamine or arginine supplementation synergises with ICB for improved antitumour immunity [67]. Glutamine supplementation via the SLC38A2 transporter increases conventional type 1 dendritic cell (cDC1)‐mediated CD8^+^ T‐cell immunity, inhibits tumour growth and overcomes therapeutic resistance to ICB [68]. Understanding the precise metabolism dynamics within immune cells opens the way to metabolic interventions to restore antitumoural immunity and response to ICB.

Although SCENITH method covers limited metabolic pathways and does not directly measure metabolic fluxes, this powerful technique remains a potent way to explore the functional metabolic heterogeneity at single cell level by uniquely allowing combining metabolism analysis with large cytometry panels including immune checkpoints, activation markers or intracellular cytokines. By complementing the SCENITH method with metabolomics and lipidomics, we could depict pertinent metabolic disturbances in circulating and tumour‐infiltrating T cell subsets and connect them with cell features while capturing the direct metabolic signature of immune cells.

Our work notably reveals metabolic disturbances of circulating and tumour‐infiltrating T cells in melanoma. It brings insights into the link between immune‐metabolic reprogramming and T‐cell dysfunctions. Such advance paves the way for appropriate tuning of metabolic pathways to rescue T cells from tumour hijacking, in order to restore potent antitumour responses and enhance the efficacy of current immunotherapies. This opens opportunities to design novel therapeutic strategies exploiting metabolic pathways to reverse immuno‐metabolic reprogramming and to overcome cancer‐associated immune subversion. Further insights into the metabolic dynamics and requirements for successful antitumour responses are promising to design pertinent combination strategies to achieve better clinical efficacy. Integration of metabolic regulation into the optimised development of cancer immunotherapies holds great promise to achieve better clinical success.

## Author Contributions

C.A. conceived the project and supervised the study. C.A., H.B. and A.T. designed the experiments. H.B., K.M., S.H., C.N., M.C.‐C. and C.A. performed the experiments and analysed the data. S.M., F.F. and J.C. provided patient material and clinical data, and expertise with clinical interpretation of the data. M.P. provided expertise with cell sorting. P.S. and A.T. provided research input and contributed to data interpretation. C.A. and H.B. wrote the manuscript. P.S., A.T., S.M. and C.N. contributed to manuscript editing.

## Funding

This work was supported by the This work was supported by the Etablissement Français du Sang (EFS) AuRA, Ligue Contre le Cancer, the Societe Française de Dermatologie et de Pathologie Sexuellement Transmissible, GEFLUC, Fondation Bristol Myers Squibb (BMS), Université Grenoble Alpes (UGA) and the Agence Nationale de la Recherche (ANR‐11‐LABX‐0021).

## Conflicts of Interest

The authors declare no conflicts of interest.

## Supporting information


**Figure S1:** Measurement of the metabolic parameters of T cell subsets using SCENITH method. PBMC from HDs and melanoma patients as well as CD45^+^ tumour‐infiltrating cells from patients were submitted to the SCENITH method to depict the metabolism of T cell subsets by flow cytometry. (A) Gating strategy of T cell subsets. Cell debris and cell doublets were first excluded using the FSC and SSC parameters. Viable (Live&Dead^−^) CD45^+^ cells were gated prior to CD3^+^ gating. From total CD3^+^ T cells, conventional T cells (Tconv) were defined as CD45^+^ CD3^+^ PanTCRγδ^−^ TCRδ2^−^. CD8^+^ and CD4^+^ T cell subsets were further depicted as CD3^+^CD8^+^ and CD3^+^CD8^−^ respectively. (B) Flow cytometry histograms depicting the incorporation of puromycin by T cell subsets in the presence of the inhibitors of glycolysis (2‐deoxy‐glucose, DG) or OXPHOS (Oligomycin, O) metabolic pathways or both inhibitors, required for SCENITH analysis.
**Figure S2:** Metabolic features of patients' T cell subsets according to clinical outcome, disease stage and previous treatment. PBMC from HDs and melanoma patients as well as CD45^+^ tumour‐infiltrating cells from patients were isolated and the metabolism of T cell subsets was depicted by flow cytometry using the SCENITH method. (A) Comparative PFS and OS (from diagnosis time) of patients displaying a high or low mitochondrial dependency, or a high or low glycolytic capacity of circulating TCD4^+^ cells (5–6 patients per group). Groups were separated using the median of the corresponding parameter for circulating T CD4^+^ cells (glucose dependency [46.1%], mitochondrial dependency [65.5%], glycolytic capacity [34.5%], FAO/AAO capacity [53.9%]). Comparisons performed using Log‐Rank test. (B, C) Comparative levels of (B) global metabolism (Mean Fluorescence Intensity (MFI) of puromycin) and (C) glycolytic or mitochondrial dependencies/capacities of T cell subsets from patient tumour according to prior treatment (immunotherapy or targeted therapy) or not. The central line indicates median. *p* values were calculated using non‐parametric two‐tailed Mann–Whitney tests (for global protein synthesis) and two‐way ANOVA with the Geisser–Greenhouse correction (for metabolic profiles).
**Figure S3:** Phenotypic profiling of T cell subsets. PBMC from HDs and melanoma patients as well as CD45^+^ tumour‐infiltrating cells from patients were isolated, and the phenotypic features of T cell subsets were depicted by flow cytometry. (A) Representative dot plots displaying expression of activation markers (CD69 and CD25) and ICPs (CTLA4, PD1, LAG3, TIM3, ICOS, TIGIT, OX40, 41BB, CD27 and GITR) on T cells from HD blood, patient blood and patient tumour (pre‐gated on alived CD45^+^ CD3^+^ cells). (B) Profile of expression of activation markers and immune checkpoints in Tconv cells from HD blood (blue, *n* = 11–25), patient blood (green, *n* = 10–25) and tumour (pink, *n* = 11–25). (C, D) Correlation matrix between the phenotypic parameters of T cell subsets (activation markers and ICPs) for (C) patient blood and (D) patient tumour. *p* values were calculated using a Spearman test followed by Holm correction. Only significant statistics are displayed on graphs. *p ≤ 0.01.
**Figure S4:** Link between the metabolic and phenotypic profiles of Tconv cells. PBMC from HDs and melanoma patients as well as CD45^+^ tumour‐infiltrating cells from patients were isolated, and the metabolism of T cell subsets was depicted using the SCENITH method together with their phenotypic features by flow cytometry. (A) Correlation matrix between the metabolic parameters of Tconv cells (global metabolism ‘puro’ as well as glycolytic/mitochondrial dependencies and capacities) and their expression of activation markers and ICPs for HD blood (*n* = 11–25), patient blood (*n* = 9–24) or tumour (*n* = 11–25). *p* values were calculated using a Spearman test followed by Holm correction. Only significant statistics are displayed on graphs. *p ≤ 0.01. (B, C) Heat map displaying the (B) global metabolism and (C) glycolytic or mitochondrial dependencies/capacities of Tconv cells, stratified according to their expression (+) or not (−) of activation markers and ICPs for HD blood (blue, *n* = 10–11), patient blood (green, *n* = 9–10) or tumour (pink, *n* = 11–13). (D) Metabolic profiles were enumerated as follows: (1) glucose dependency, (2) mitochondrial dependency, (3) glycolytic capacity, and (4) FAO/AAO capacity. (D, E) Heat map displaying the expression levels of activation markers and ICPs on Tconv cells stratified according to their low or high (D) global metabolism and (E) glycolytic or mitochondrial dependencies/capacities, for HD blood (blue, *n* = 10–11), patient blood (green, *n* = 9–10) or tumour (pink, *n* = 11–13). Groups were separated using the median of the metabolic parameter Tconv cells.
**Figure S5:** Comparative metabolism of CD8^+^ T cells according to their expression or not of activation markers and ICPs. PBMC from HDs and melanoma patients as well as CD45^+^ tumour‐infiltrating cells from patients were isolated, and the metabolism of CD8^+^ T cells was depicted using the SCENITH method together with their phenotypic features by flow cytometry. (A, B, C, D) Comparative levels of global metabolism (left panel) and glycolytic or mitochondrial dependencies/capacities (right panels) of CD8^+^ T cells according to their expression (+) or not (−) of activation markers (A) and ICPs (B, C, D) for HD blood (blue, *n* = 10–11), patient blood (green, *n* = 9–10) or tumour (pink, *n* = 11–13). *p* values were calculated using RM two‐way ANOVA with Sidak's multiple comparison post‐test (for intra‐group comparisons) or non‐parametric two‐way ANOVA or a mixed effect model followed by Tukey's multiple comparison post‐tests (for inter‐groups comparisons).
**Figure 6:** Comparative metabolism of CD4^+^ T cells according to their expression or not of activation markers and ICPs. PBMC from HDs and melanoma patients as well as CD45^+^ tumour‐infiltrating cells from patients were isolated, and the metabolism of CD4^+^ T cells was depicted using the SCENITH method together with their phenotypic features by flow cytometry. (A, B, C, D) Comparative levels of global metabolism (left panel) and glycolytic or mitochondrial dependencies/capacities (right panels) of CD4^+^ T cells according to their expression (+) or not (−) of activation markers (A) and ICPs (B, C, D) for HD blood (blue, *n* = 10–11), patient blood (green, *n* = 9–10) or tumour (pink, *n* = 11–13). *p* values were calculated using RM two‐way ANOVA with Sidak's multiple comparison post‐test (for intra‐group comparisons) or non‐parametric two‐way ANOVA or a mixed effect model followed by Tukey's multiple comparison post‐tests (for inter‐groups comparisons).
**Figure S7:** Link between the metabolic status of T cell subsets and the composition of their secretome at steady state. PBMC from HDs and melanoma patients as well as CD45^+^ tumour‐infiltrating cells from patients were cultured for 20 h, and their metabolism was depicted using the SCENITH method simultaneously to measurement of their secretome by Luminex. (A) Comparative levels of secreted cytokines from HD blood (blue, *n* = 10), patient blood (green, *n* = 10) or tumour (pink, *n* = 12). Bars indicate median. *p* values were calculated using non‐parametric two‐way ANOVA or a mixed effect model followed by Tukey's multiple comparison post‐tests. Only significant statistics are displayed on graphs. **p ≤ 0.01, ****p ≤ 0.0001. (B) Correlation matrix between the metabolic parameters of T cell subsets (global metabolism (MFI of puromycin) and glycolytic or mitochondrial dependencies/capacities) and cytokine secretion levels from HD blood (blue, *n* = 9), patient blood (green, *n* = 9) or tumour (pink, *n* = 9). *p* values were calculated using a Spearman test followed by Holm correction. Only significant statistics are displayed on graphs. *p ≤ 0.01.
**Figure S8:** Remodelling of the expression profiles of activation markers and immune checkpoints by T cell subsets upon stimulation. PBMC from HDs and melanoma patients as well as CD45^+^ tumour‐infiltrating cells from patients were stimulated or not with either PMA/iono or anti‐CD3/CD28 antibodies, and their phenotypic features were depicted by flow cytometry. Profile of expression of activation markers and immune checkpoints in CD8^+^ and CD4^+^ T cells in the different conditions of stimulation for HD blood (blue, *n* = 10–11), patient blood (green, *n* = 8–10) and tumour (pink, *n* = 8–13). *p* values were calculated using non‐parametric two‐way ANOVA or a mixed effect model followed by Tukey's multiple comparison post‐tests. Only significant statistics are displayed on graphs. *p ≤ 0.05, **p ≤ 0.01, ***p ≤ 0.001, ****p ≤ 0.0001.
**Figure S9:** The remodelling of the expression profiles of activation markers and immune checkpoints by T cell subsets upon stimulation is linked with metabolic reprogramming in melanoma. PBMC from HDs and melanoma patients as well as CD45^+^ tumour‐infiltrating cells from patients were stimulated or not with either PMA/iono or anti‐CD3/CD28 antibodies, and the metabolism of T cell subsets was depicted using the SCENITH method along with their phenotypic features by flow cytometry. (A) Correlation matrix between the metabolic parameters of T cell subsets (global metabolism ‘puro’ as well as glycolytic/mitochondrial dependencies and capacities) and their expression of activation markers and ICPs upon anti‐CD3/CD28 stimulation for HD blood, patient blood or tumour. *p* values were calculated using a Spearman test followed by Holm correction. Only significant statistics are displayed on graphs. *p ≤ 0.01. (B, C) Heat map displaying the (B) global metabolism and (C) glycolytic or mitochondrial dependencies/capacities of T cell subsets, stratified according to their expression (+) or not (−) of activation markers and ICP upon anti‐CD3/CD28 stimulation for HD blood (blue, *n* = 9–10), patient blood (green, *n* = 8–9) or tumour (pink, *n* = 8–12). (C) Metabolic profiles were enumerated as follows: (1) glucose dependency, (2) mitochondrial dependency, (3) glycolytic capacity, and (4) FAO/AAO capacity. (D, E) Heat map displaying the expression levels of activation markers and ICPs on T cell subsets stratified according to their low or high (D) global metabolism and (E) glycolytic or mitochondrial dependencies/capacities upon anti‐CD3/CD28 stimulation for HD blood (blue, *n* = 10–11), patient blood (green, *n* = 8–9) or tumour (pink, *n* = 8–12). Groups were separated using the median of the metabolic parameter in the corresponding T cell subsets.
**Figure S10:** Comparative metabolism of CD8^+^ T cells according to their expression or not of activation markers and ICPs upon PMA/iono stimulation. PBMC from HDs and melanoma patients as well as CD45^+^ tumour‐infiltrating cells from patients were stimulated with PMA/iono, and the metabolism of CD8^+^ T cells was depicted using the SCENITH method together with their phenotypic features by flow cytometry. (A, B, C, D) Comparative levels of global metabolism (left panel) and glycolytic or mitochondrial dependencies/capacities (right panels) of CD8^+^ T cells according to their expression (+) or not (−) of activation markers (A) and ICPs (B, C, D) upon PMA/iono stimulation for HD blood (blue, *n* = 9–10), patient blood (green, *n* = 8–9) or tumour (pink, *n* = 8–12). *p* values were calculated using RM two‐way ANOVA with Sidak's multiple comparison post‐test (for intra‐group comparisons) or non‐parametric two‐way ANOVA or a mixed effect model followed by Tukey's multiple comparison post‐tests (for inter‐groups comparisons).
**Figure S11:** Comparative metabolism of CD4^+^ T cells according to their expression or not of activation markers and ICPs upon PMA/iono stimulation. PBMC from HDs and melanoma patients as well as CD45^+^ tumour‐infiltrating cells from patients were stimulated with PMA/iono, and the metabolism of CD4^+^ T cells was depicted using the SCENITH method together with their phenotypic features by flow cytometry. (A, B, C, D) Comparative levels of global metabolism (left panel) and glycolytic or mitochondrial dependencies/capacities (right panels) of CD4^+^ T cells according to their expression (+) or not (−) of activation markers (A) and ICPs (B, C, D) upon PMA/iono stimulation for HD blood (blue, *n* = 9–10), patient blood (green, *n* = 8–9) or tumour (pink, *n* = 8–12). *p* values were calculated using RM two‐way ANOVA with Sidak's multiple comparison post‐test (for intra‐group comparisons) or non‐parametric two‐way ANOVA or a mixed effect model followed by Tukey's multiple comparison post‐tests (for inter‐groups comparisons).
**Figure S12:** Comparative metabolism of CD8^+^ T cells according to their expression or not of activation markers and ICPs upon anti‐CD3/CD28 stimulation. PBMC from HDs and melanoma patients as well as CD45^+^ tumour‐infiltrating cells from patients were stimulated with anti‐CD3/CD28, and the metabolism of CD8^+^ T cells was depicted using the SCENITH method together with their phenotypic features by flow cytometry. (A, B, C, D) Comparative levels of global metabolism (left panel) and glycolytic or mitochondrial dependencies/capacities (right panels) of CD8^+^ T cells according to their expression (+) or not (−) of activation markers (A) and ICPs (B, C, D) upon anti‐CD3/CD28 stimulation for HD blood (blue, *n* = 9–10), patient blood (green, *n* = 8–9) or tumour (pink, *n* = 8–12). *p* values were calculated using RM two‐way ANOVA with Sidak's multiple comparison post‐test (for intra‐group comparisons) or non‐parametric two‐way ANOVA or a mixed effect model followed by Tukey's multiple comparison post‐tests (for inter‐groups comparisons).
**Figure S13:** Comparative metabolism of CD4^+^ T cells according to their expression or not of activation markers and ICPs upon anti‐CD3/CD28 stimulation. PBMC from HDs and melanoma patients as well as CD45^+^ tumour‐infiltrating cells from patients were stimulated with anti‐CD3/CD28, and the metabolism of CD8^+^ T cells was depicted using the SCENITH method together with their phenotypic features by flow cytometry. (A, B, C, D) Comparative levels of global metabolism (left panel) and glycolytic or mitochondrial dependencies/capacities (right panels) of CD4^+^ T cells according to their expression (+) or not (−) of activation markers (A) and ICPs (B, C, D) upon anti‐CD3/CD28 stimulation for HD blood (blue, *n* = 9–10), patient blood (green, *n* = 8–9) or tumour (pink, *n* = 8–12). *p* values were calculated using RM two‐way ANOVA with Sidak's multiple comparison post‐test (for intra‐group comparisons) or non‐parametric two‐way ANOVA or a mixed effect model followed by Tukey's multiple comparison post‐tests (for inter‐groups comparisons).
**Figure S14:** Functional orientation of T cell subsets upon stimulation. PBMC from HDs and melanoma patients as well as CD45^+^ tumour‐infiltrating cells from patients were stimulated or not with either PMA/iono or anti‐CD3/CD28 antibodies, and their metabolism was depicted using the SCENITH method simultaneously to intracellular cytokine measurements by flow cytometry. (A) Profile of cytokine expression by CD8^+^ (upper panel) and CD4^+^ (lower panel) T cells in the different conditions of stimulation for HD blood (blue, *n* = 8–10), patient blood (green, *n* = 8–10) and tumour (pink, *n* = 11–13). (B) Correlation matrix between the proportions of cytokine‐expressing T cell subsets upon PMA/iono (left panel) or anti‐CD3CD28 (right panel) stimulation. *p* values were calculated using a Spearman test followed by Holm correction. Only significant statistics are displayed on graphs. *p ≤ 0.01.
**Figure S15:** The cytokine expression profiles of T cell subsets upon stimulation is linked with metabolic reprogramming in melanoma. PBMC from HDs and melanoma patients as well as CD45^+^ tumour‐infiltrating cells from patients were stimulated or not with anti‐CD3/CD28 antibodies, and their metabolism was depicted using the SCENITH method simultaneously to intracellular cytokine measurements by flow cytometry. (A) Correlation matrix between the metabolic parameters of T cell subsets (global metabolism ‘puro’ as well as glycolytic/mitochondrial dependencies and capacities) and their cytokine expression upon anti‐CD3/CD28 stimulation for HD blood (blue, *n* = 10–11), patient blood (green, *n* = 9–10) or tumour (pink, *n* = 11–12). *p* values were calculated using a Spearman test followed by Holm correction. Only significant statistics are displayed on graphs. *p ≤ 0.01. (B) Heat map displaying the global metabolism of T cell subsets, stratified according to their expression (+) or not (−) of cytokines upon anti‐CD3/CD28 for HD blood (blue, *n* = 10–11), patient blood (green, *n* = 8–10) or tumour (pink, *n* = 12–13). (C) Heat map displaying the glycolytic or mitochondrial dependencies/capacities of T cell subsets, stratified according to their expression (+) or not (−) of cytokines upon anti‐CD3/CD28 stimulation for HD blood (blue, *n* = 10–11), patient blood (green, *n* = 8–10) or tumour (pink, *n* = 12–13). Metabolic profiles were enumerated as follows: (1) glucose dependency, (2) mitochondrial dependency, (3) glycolytic capacity, and (4) FAO/AAO capacity. (D) Heat map displaying cytokine expression levels of T cell subsets stratified according to their low or high global metabolism upon anti‐CD3/CD28 stimulation for HD blood (blue, *n* = 10–11), patient blood (green, *n* = 9–10) or tumour (pink, *n* = 11–13). Groups were separated using the median of the metabolic parameter in the corresponding T cell subsets. (E) Heat map displaying cytokine expression levels of T cell subsets stratified according to their low or high glycolytic or mitochondrial dependencies/capacities upon anti‐CD3/CD28 stimulation for HD blood (blue, *n* = 10–11), patient blood (green, *n* = 9–10) or tumour (pink, *n* = 11–13). Groups were separated using the median of the metabolic parameter in the corresponding T cell subsets. All significant statistics are displayed on corresponding graphs in Figures S16 and S17.
**Figure S16:** Comparative metabolism of CD8^+^ T cells according to their cytokine production profile upon stimulation. PBMC from HDs and melanoma patients as well as CD45^+^ tumour‐infiltrating cells from patients were stimulated with PMA/iono or anti‐CD3/CD28, and the metabolism of CD8^+^ T cells was depicted using the SCENITH method simultaneously to intracellular cytokine measurements by flow cytometry. (A, B) Comparative levels of global metabolism (left panel) and glycolytic or mitochondrial dependencies/capacities (right panels) of CD8^+^ T cells according to their expression (+) or not (−) of cytokines upon PMA/iono (A) or anti‐CD3/CD28 (B) stimulation for HD blood (blue, *n* = 10–11), patient blood (green, *n* = 8–10) or tumour (pink, *n* = 12–13). *p* values were calculated using RM two‐way ANOVA with Sidak's multiple comparison post‐test (for intra‐group comparisons) or non‐parametric two‐way ANOVA or a mixed effect model followed by Tukey's multiple comparison post‐tests (for inter‐groups comparisons).
**Figure S17:** Comparative metabolism of CD4^+^ T cells according to their cytokine production profile upon stimulation. PBMC from HDs and melanoma patients as well as CD45^+^ tumour‐infiltrating cells from patients were stimulated with PMA/iono or anti‐CD3/CD28, and the metabolism of CD4^+^ T cells was depicted using the SCENITH method simultaneously to intracellular cytokine measurements by flow cytometry. (A, B) Comparative levels of global metabolism (left panel) and glycolytic or mitochondrial dependencies/capacities (right panels) of CD4^+^ T cells according to their expression (+) or not (−) of cytokines upon PMA/iono (A) or anti‐CD3/CD28 (B) stimulation for HD blood (blue, *n* = 10–11), patient blood (green, *n* = 8–10) or tumour (pink, *n* = 12–13). *p* values were calculated using RM two‐way ANOVA with Sidak's multiple comparison post‐test (for intra‐group comparisons) or non‐parametric two‐way ANOVA or a mixed effect model followed by Tukey's multiple comparison post‐tests (for inter‐groups comparisons).
**Figure S18:** Link between the metabolic status of T cell subsets and the composition of their secretome under stimulation. PBMC from HDs and melanoma patients as well as CD45^+^ tumour‐infiltrating cells from patients were stimulated or not with either PMA/iono or anti‐CD3/CD28 antibodies, and their metabolism was depicted using the SCENITH method simultaneously to measurement of their secretome by Luminex. Comparative levels of secreted cytokines from HD blood (blue, *n* = 10), patient blood (green, *n* = 10) or tumour (pink, *n* = 11–13) upon stimulation (PMA/iono or anti‐CD3/CD28) or not. Bars indicate median. *p* values were calculated using non‐parametric two‐way ANOVA or a mixed effect model followed by Tukey's multiple comparison post‐tests. Only significant statistics are displayed on graphs. *p ≤ 0.05, **p ≤ 0.01, ***p ≤ 0.001, ****p ≤ 0.0001. (B, C) Correlation matrix between the metabolic parameters of T cell subsets (global metabolism (MFI of puromycin) and glycolytic or mitochondrial dependencies/capacities) and cytokine secretion levels from HD blood (blue, *n* = 10), patient blood (green, *n* = 10) or tumour (pink, *n* = 12–13) upon PMA/iono (B) or anti‐CD3/CD28 (C) stimulation. *p* values were calculated using a Spearman test followed by Holm correction. Only significant statistics are displayed on graphs. *p ≤ 0.01.
**Figure S19:** Strategy of Tyto sorting and LC–MS analysis. PBMC from HDs and melanoma patients as well as CD45^+^ tumour‐infiltrating cells from patients were submitted to Tyto sorting to purify CD4^+^ and CD8^+^ T cells. Metabolomic and lipidomic analysis were then performed by LC–MS. (A) Experimental design. Created in BioRender. (B) Representative dot plots displaying the purity of CD4^+^ T cells (left panels) and CD8^+^ T cells (right panels) obtained after Tyto sorting from PBMC of HD as well as PBMC and tumour of patients (pre‐gated on single alive CD45^+^ cells).
**Figure 20:** Metabolomic and lipidomic deregulations in circulating CD4^+^ T cells from patients. CD4^+^ T cells were isolated from PBMC of HDs and melanoma patients as well as tumour‐infiltrating immune cells from patients using Tyto sorter and conserved in methanol, H_2_O and formic acid solution at −80°C. Cell subset metabolomic and lipidomic was performed using LC–MS method. (A) Bar plots representing the top 12 metabolites and lipid changes sorted by log of Fold Change between HD blood (*n* = 4) and patient blood (*n* = 6) after *limma* analysis (Table S3). Blue: downregulation, red: upregulation of the metabolites in patient's tumour compared to HD blood. (B, C) Enrichment's pathway and mapping of the top 12 changes in metabolites between HD blood (*n* = 4) and patient blood (*n* = 6) using MetaboAnalyst software. (D) Representation of the top 12 changes in lipids among lipid classes and subclasses for differences between HD blood (*n* = 4) and patient blood (*n* = 6).
**Figure S21:** Metabolomic and lipidomic deregulation in circulating CD8^+^ T cells from patients. CD8^+^ T cells were isolated from PBMC from HDs and melanoma patients as well as CD45^+^ tumour‐infiltrating cells from patients using Tyto sorter and conserved in methanol, H_2_O and formic acid solution at −80°C. Cell subset metabolomic and lipidomic was performed using LC–MS method. (A) Bar plots representing the top 12 metabolites and lipid changes sorted by log of Fold Change between HD blood (*n* = 4) and patient blood (*n* = 6) after *limma* analysis (Table S3). Blue: downregulation, red: upregulation of the metabolites in patient's tumour compared to HD blood. (B, C) Enrichment's pathway and mapping of the top 12 changes in metabolites between HD blood (*n* = 4) and patient blood (*n* = 6) using MetaboAnalyst software. (D) Representation of the top 12 changes in lipids among lipid classes and subclasses for differences between HD blood (*n* = 4) and patient blood (*n* = 6).
**Figure S22:** Expression of the candidate enzymes and transporters connected to the perturbated metabolites in tumour‐infiltrating T cells was not correlated with tumour cells Correlation of the gene expression of candidate enzymes and nutrient transporters with the melanoma specific *MLANA/MITF* genes using RNA sequencing data from the TCGA dataset (SKCM cohort, *n* = 461 patients) using the GEPIA2 server.

**Table S1:** Clinical features of patients used in the study.
**Table S2:** Results of Log‐Rank tests run between the metabolic features of T cell subsets and patient's PFS and OS.
**Table S3:** Differential abundance analysis of general metabolites found in CD4 and CD8 T cells between patient's blood or tumour and HD's blood.
**Table S4:** Differential abundance analysis of lipids found in CD4 and CD8 T cells between patient's blood or tumour and HD's blood.
**Table S5:** List of upstream and downstream enzymes as well as transporters connected with the metabolites that were found to be perturbated within tumour‐infiltrating CD4+ and CD8+ T cells.
**Table S6:** List of enzymes/transporters found to be linked with melanoma patients' clinical outcome.

## Data Availability

The data that support the findings of this study are available from the corresponding author upon reasonable request.
