# Supplementary material for: Metabolic Profiling of Circulating and Tumour‐Infiltrating T Cells Uncovers Bioenergetic Checkpoints Linked With Immune and Clinical Skewing in Melanoma Patients
Source: Immunology. 2026 Aug 4;179(3):486–515. doi: 10.1111/imm.70185 (PMC13636174; doi:10.1111/imm.70185)

Supplementary Figure 1

A

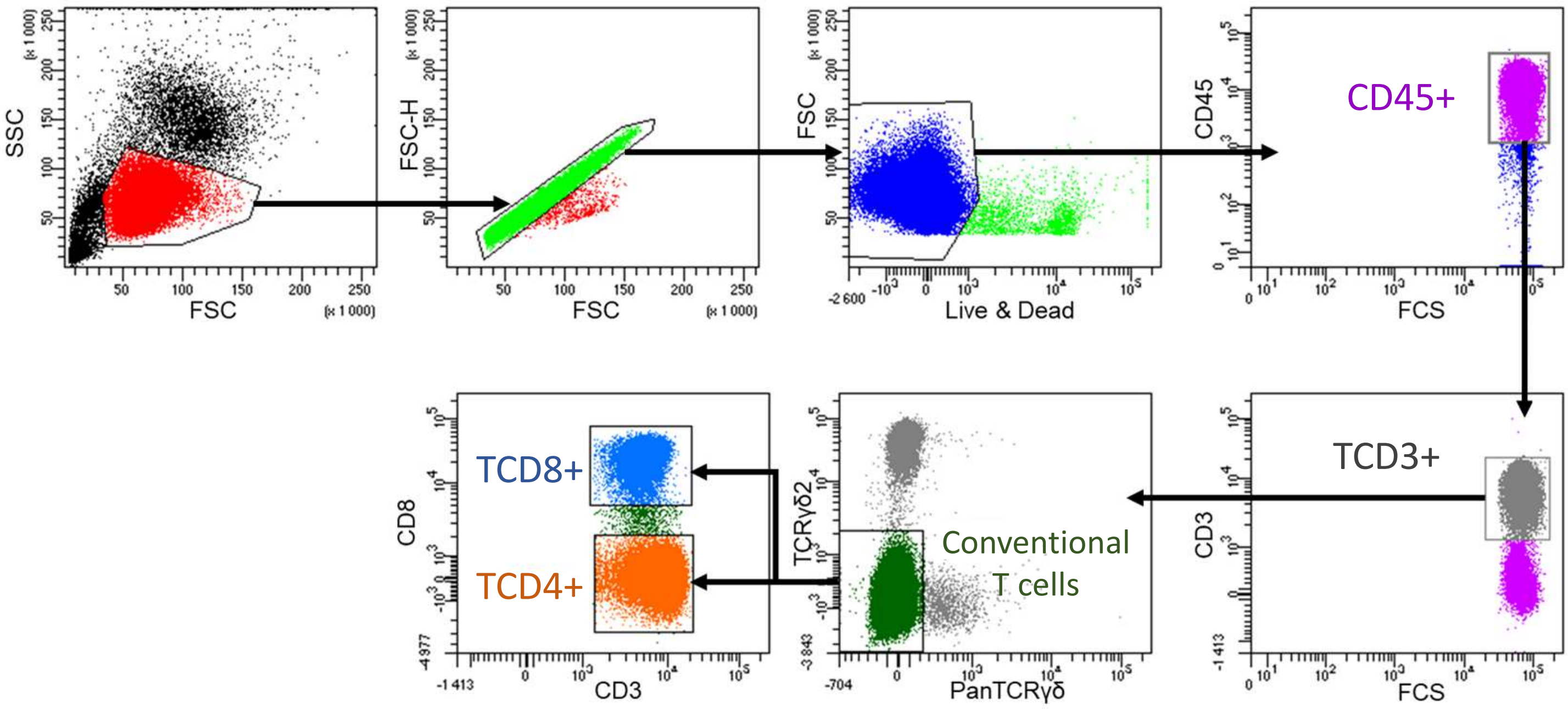

B

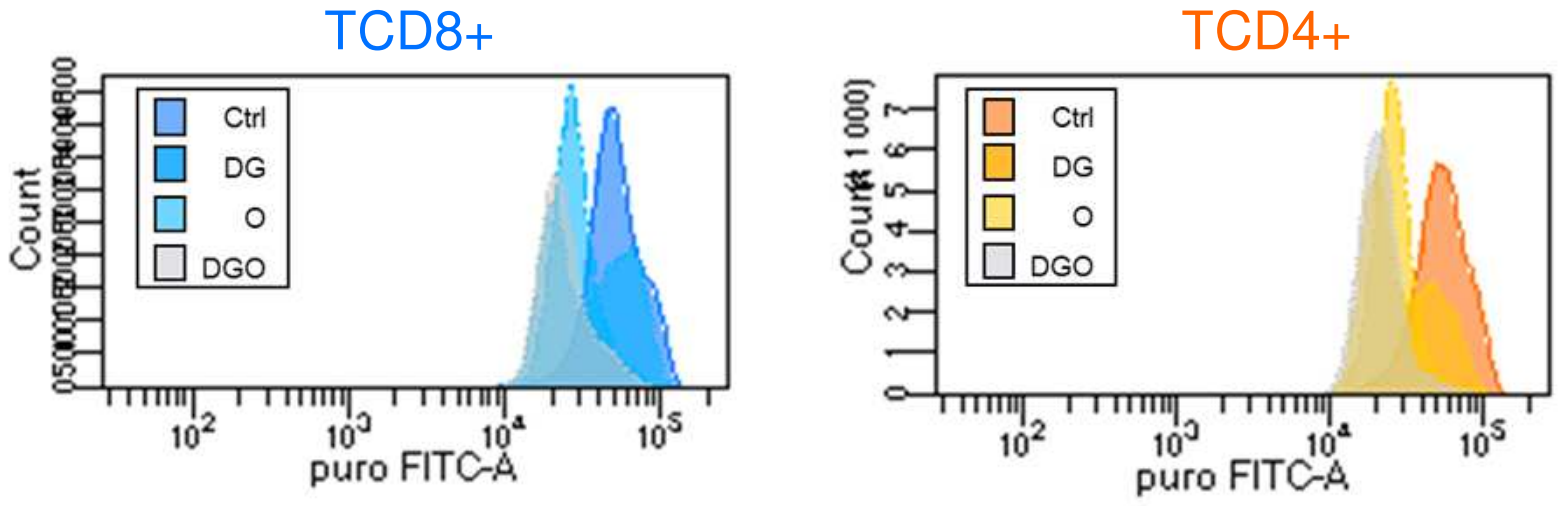

Supplementary Figure 2

A Patient Blood (diagnosis time)

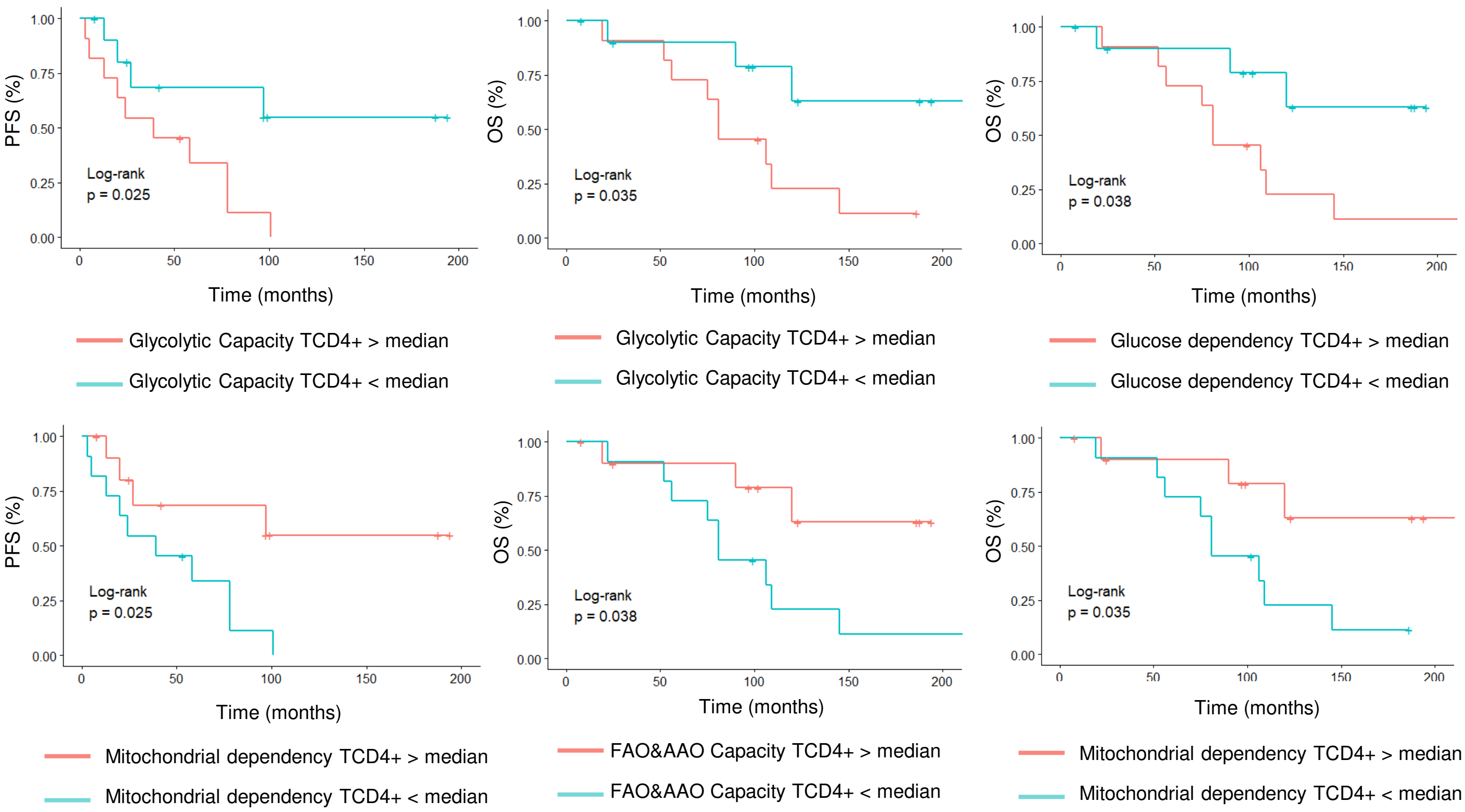

B

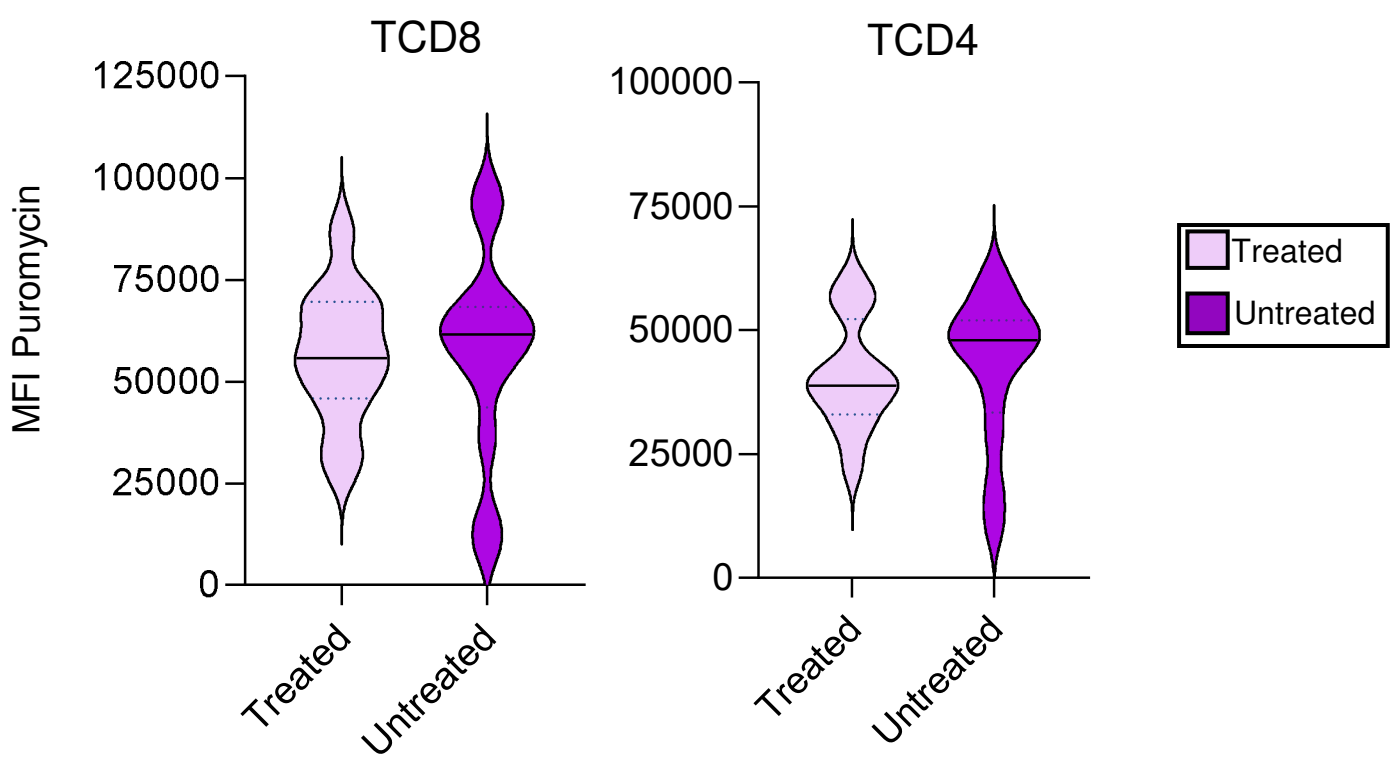

C

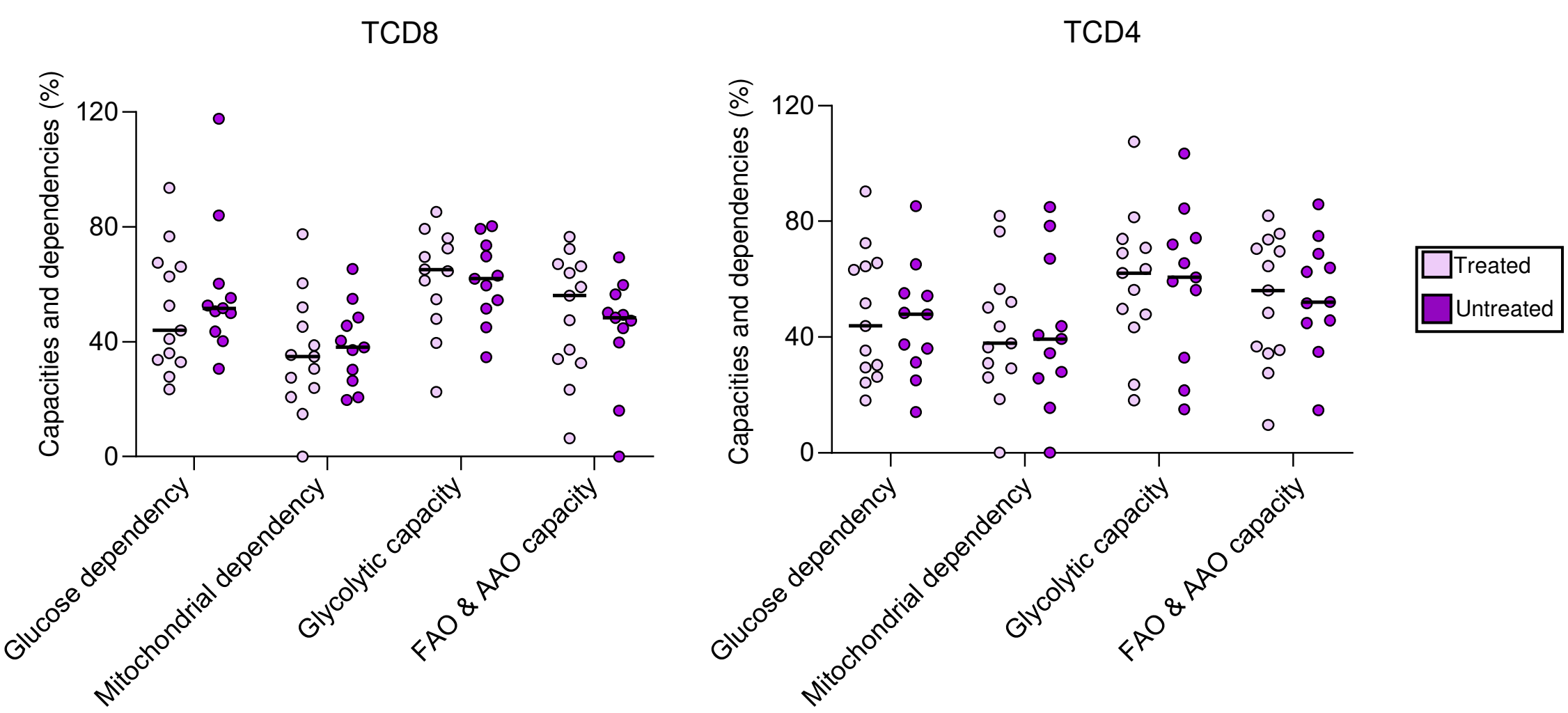

Supplementary Figure 3

A

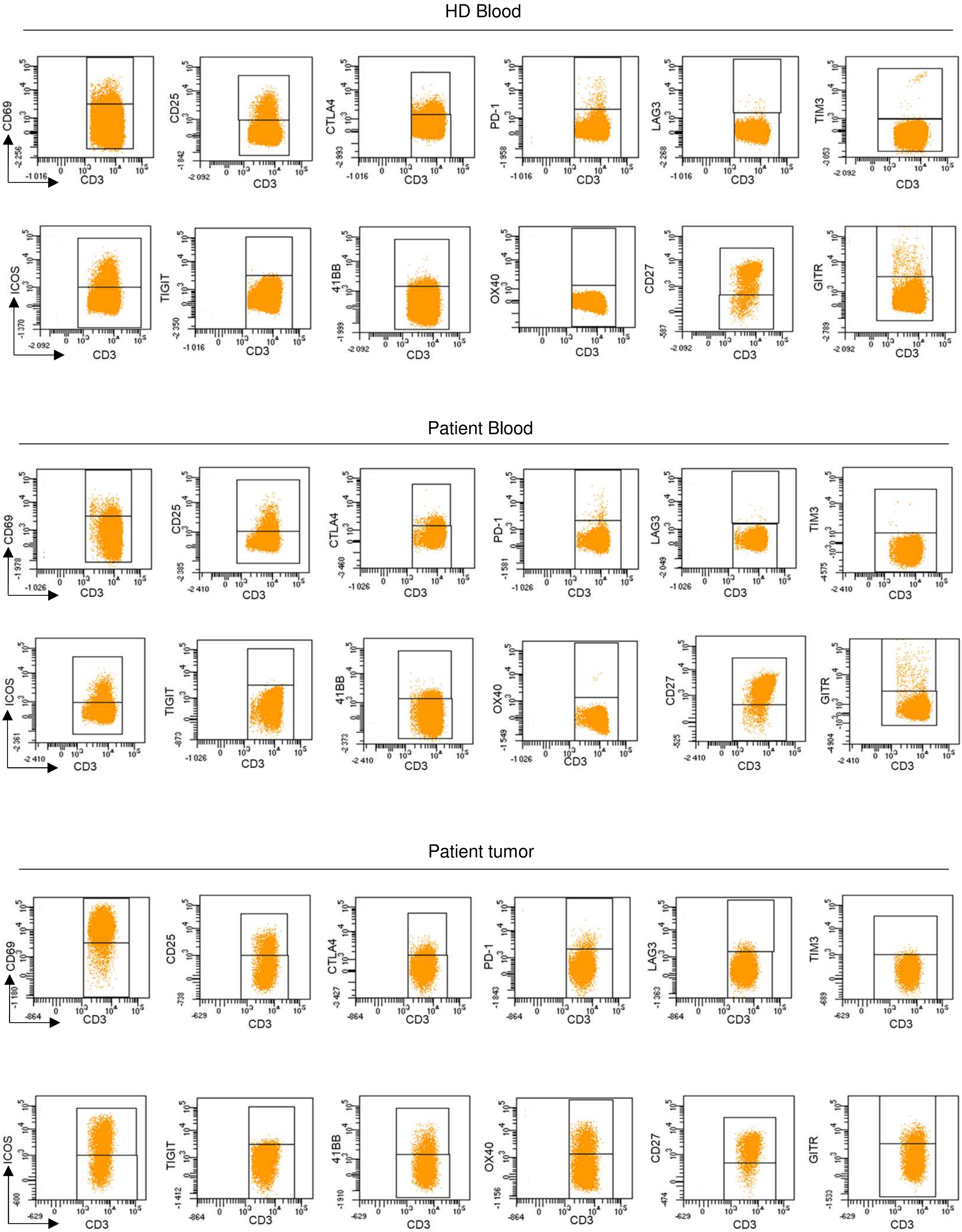

Supplementary Figure 3 (following)

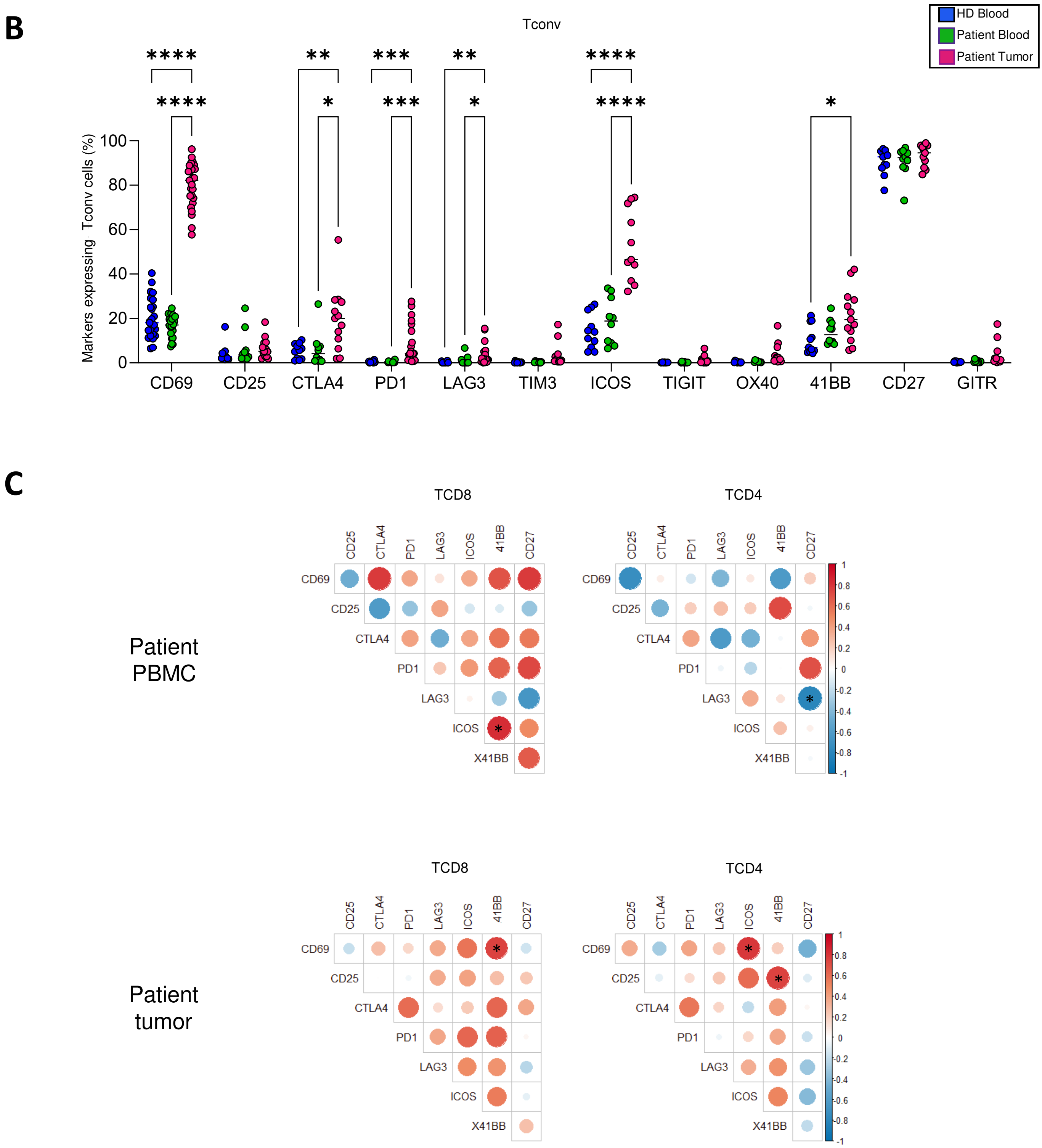

Supplementary Figure 4

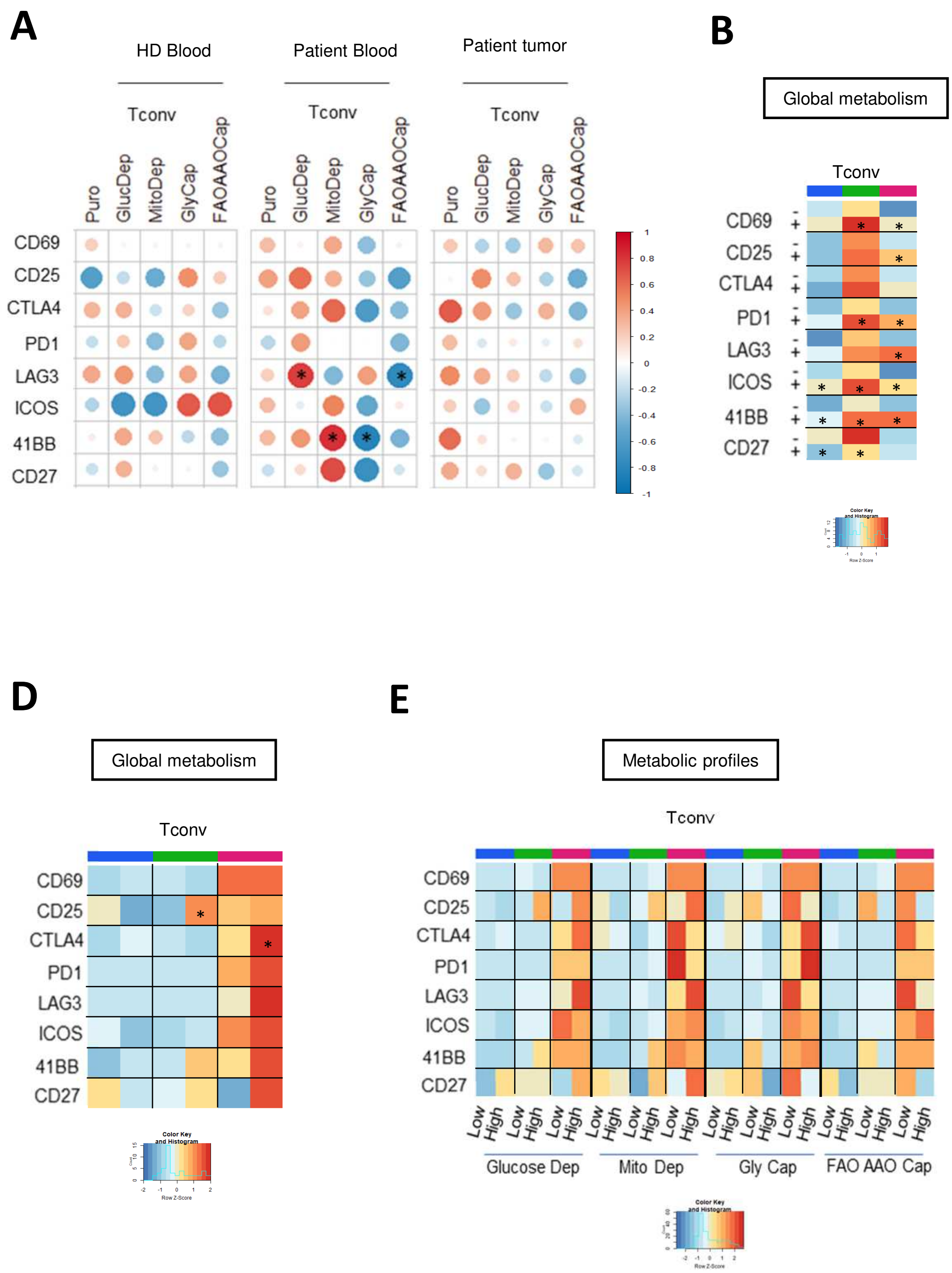

Supplementary Figure 5

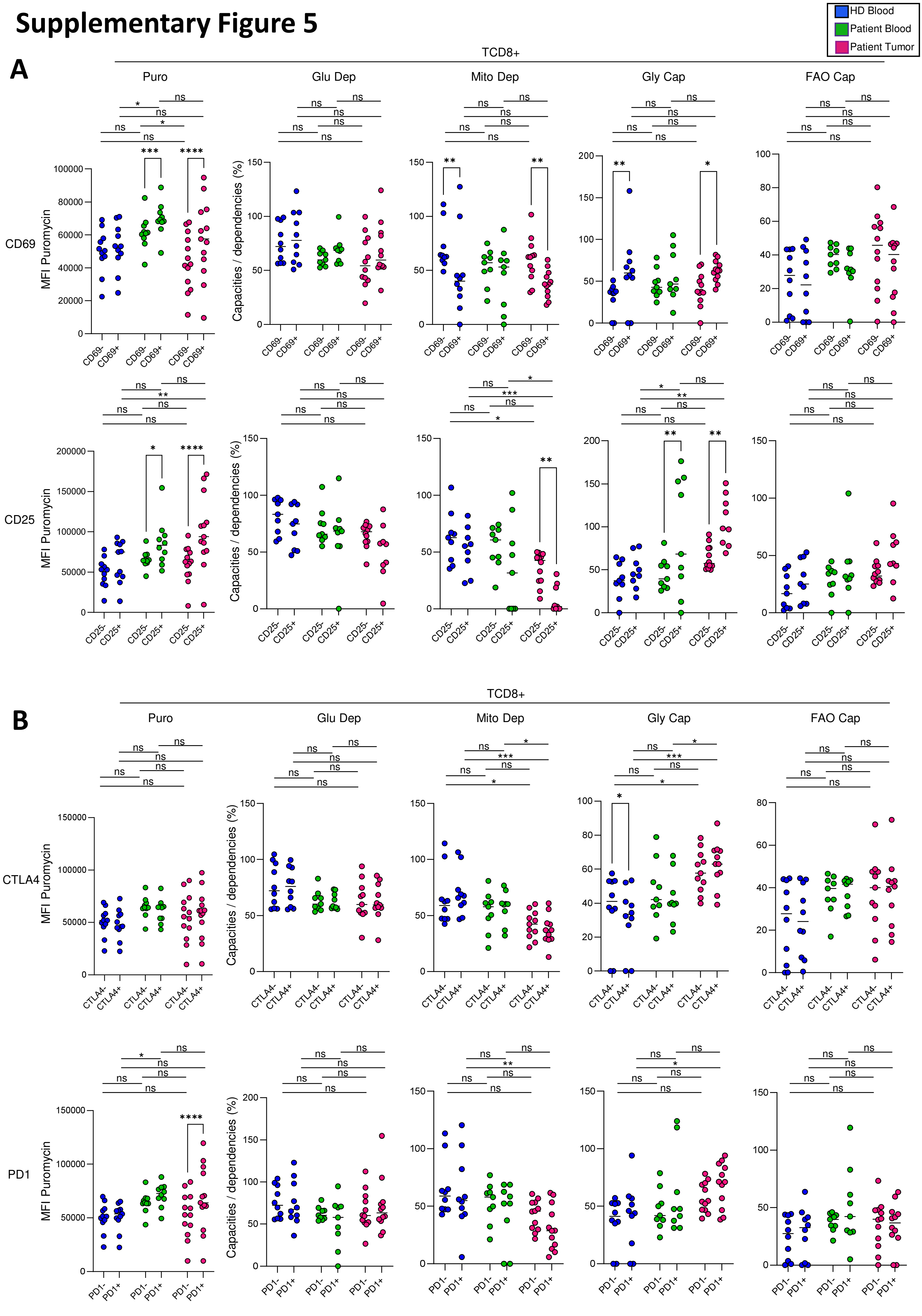

Supplementary Figure 5 (following)

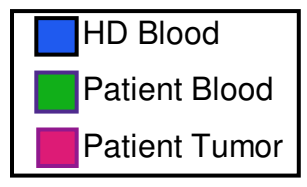

C

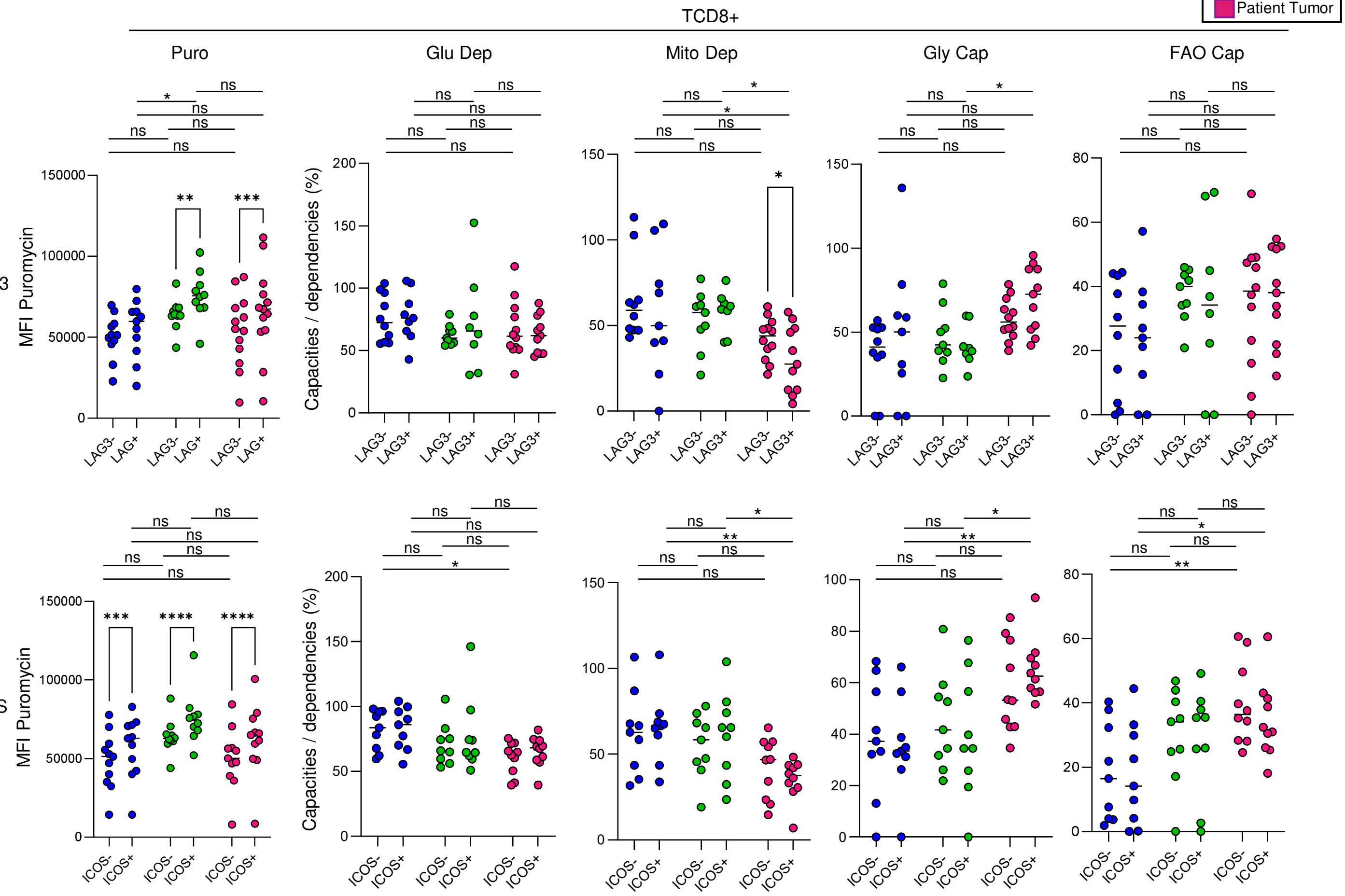

D

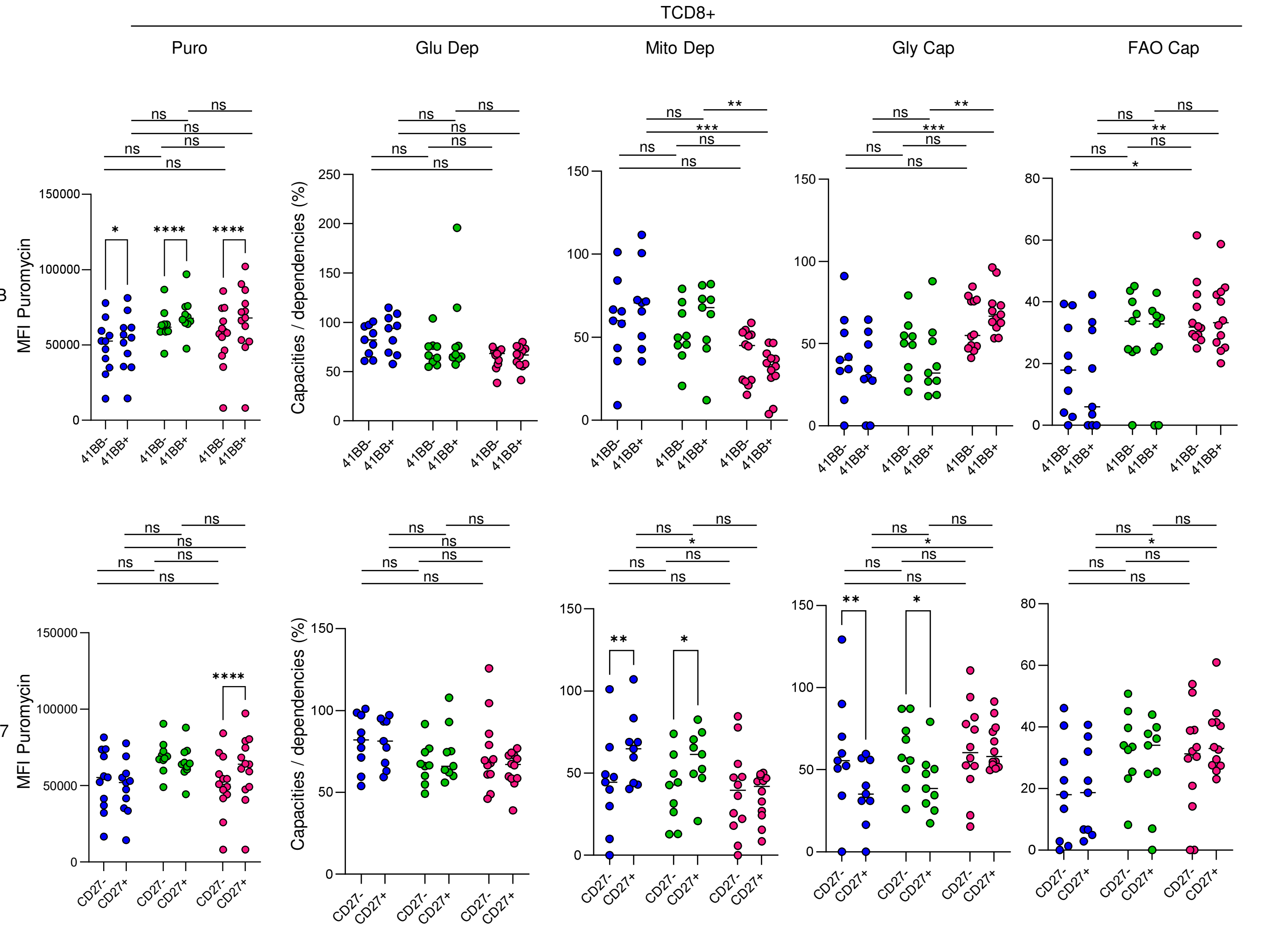

Supplementary Figure 6

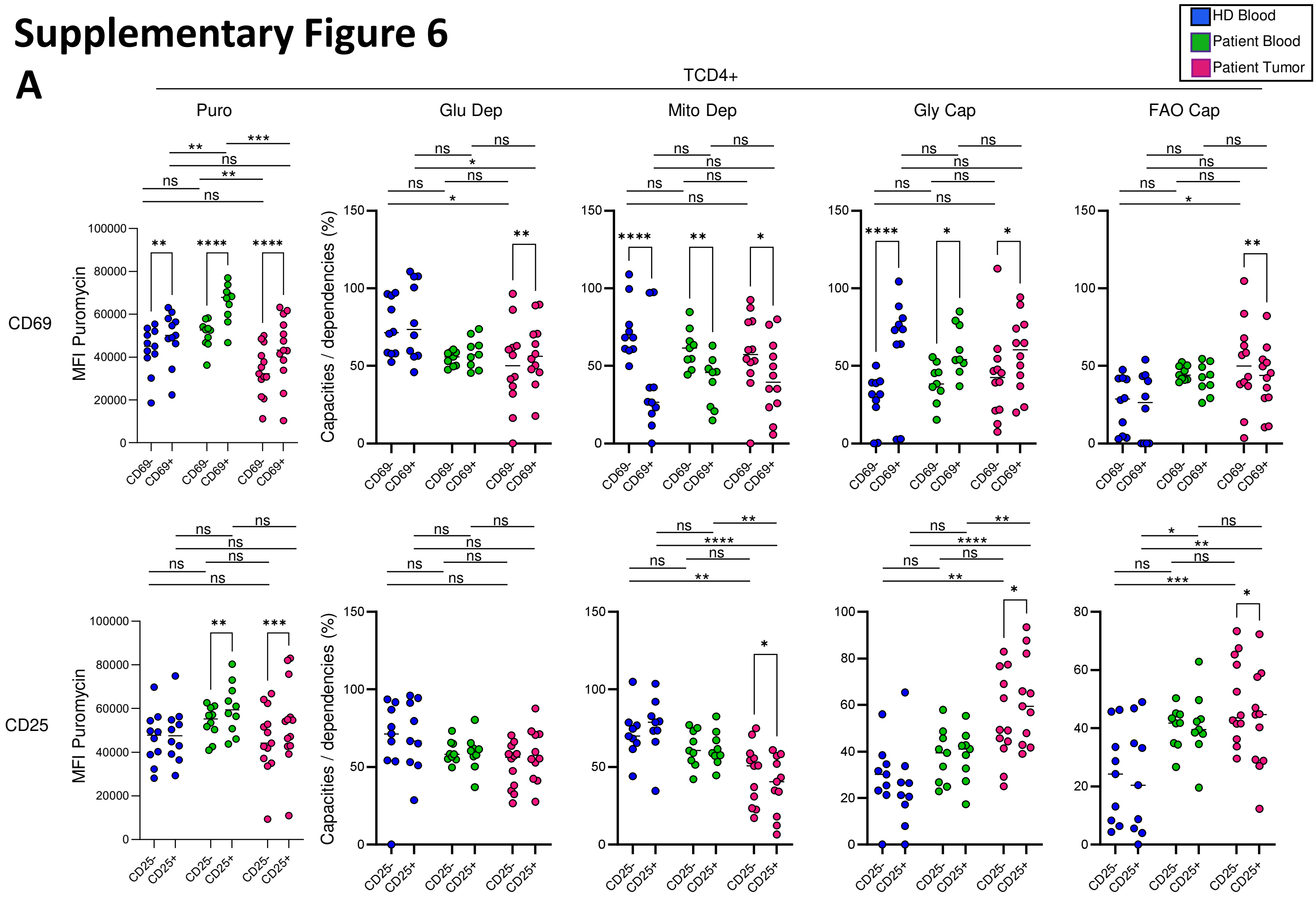

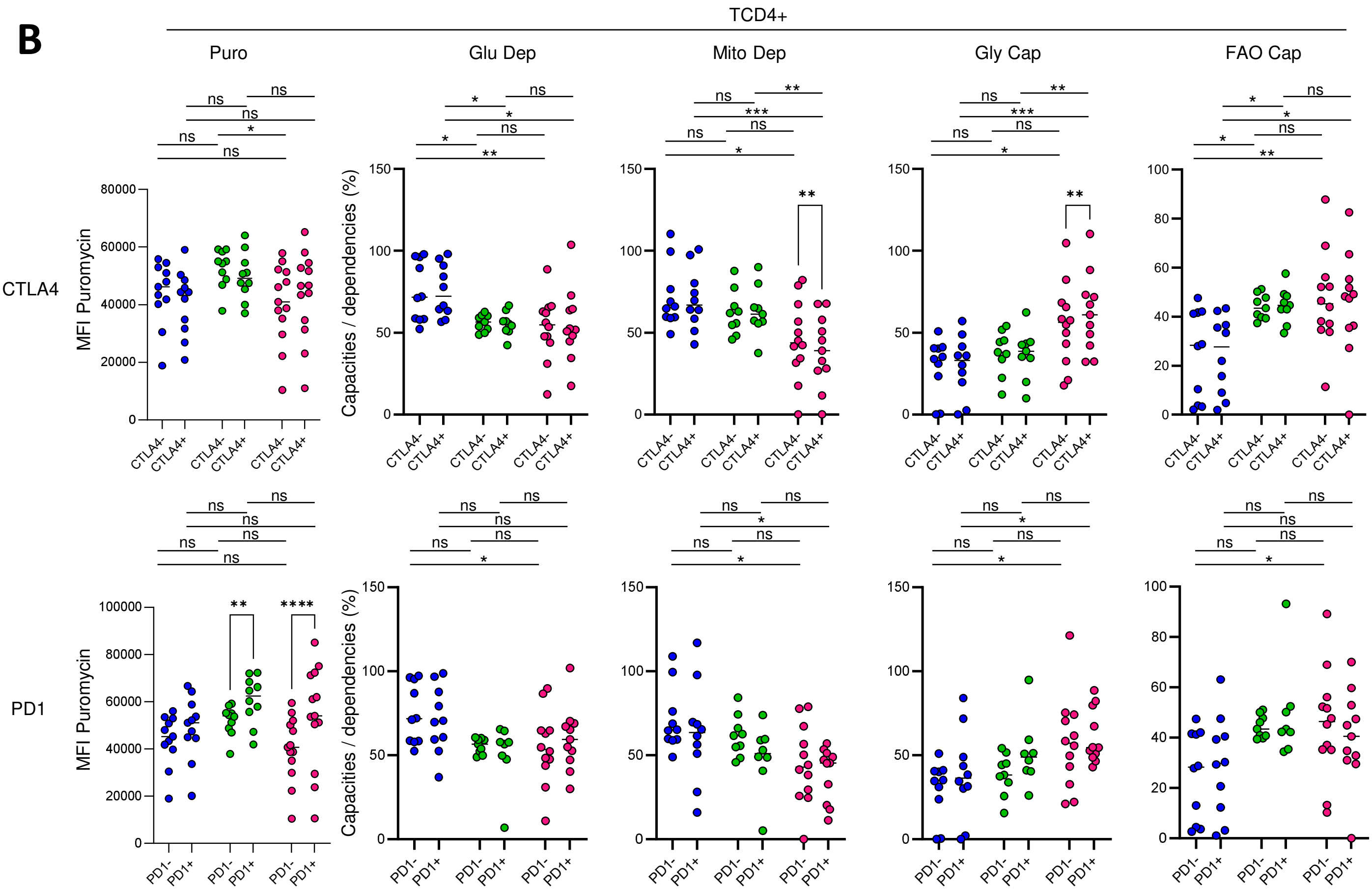

Supplementary Figure 6 (following)

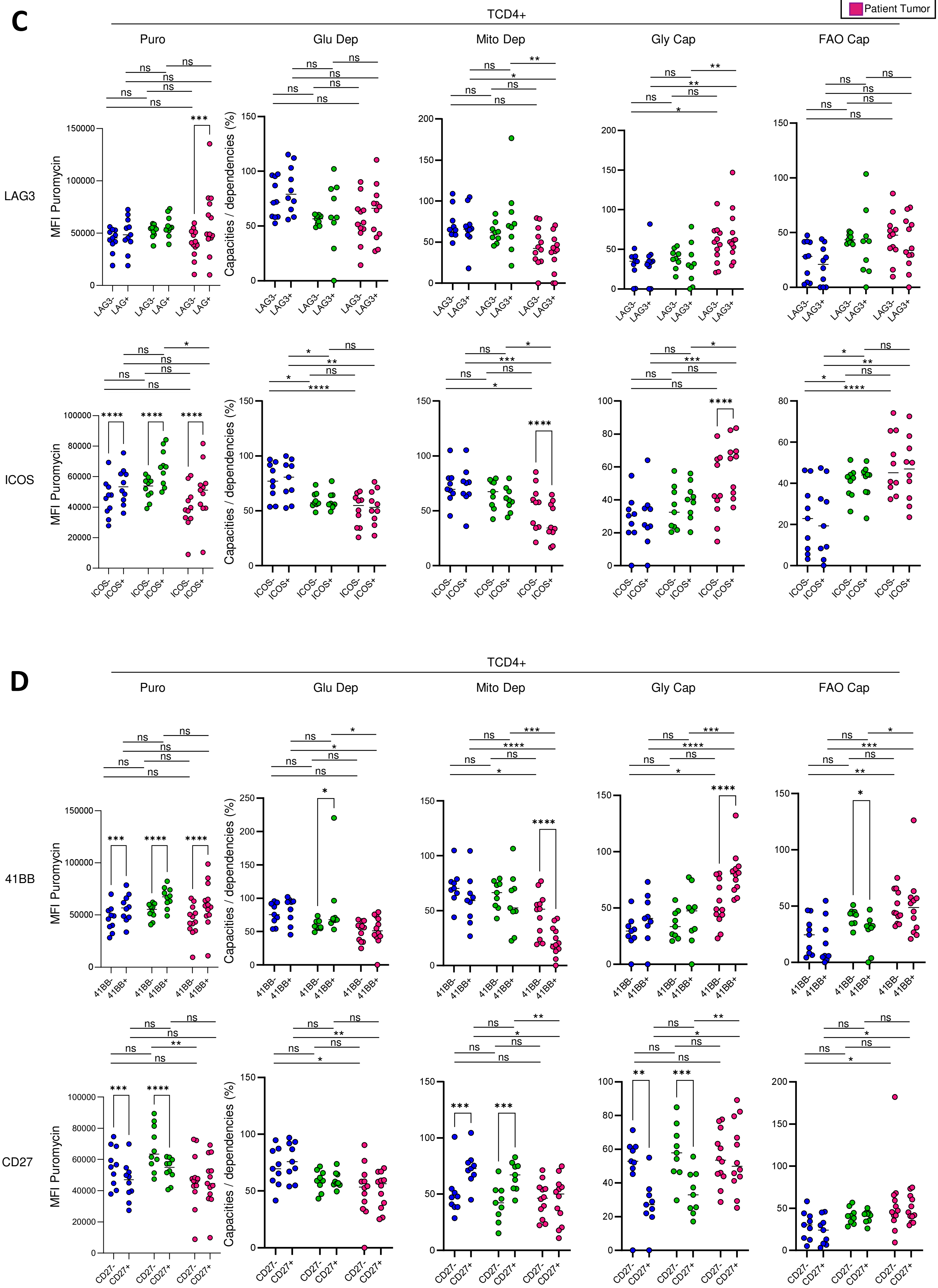

Supplementary Figure 7

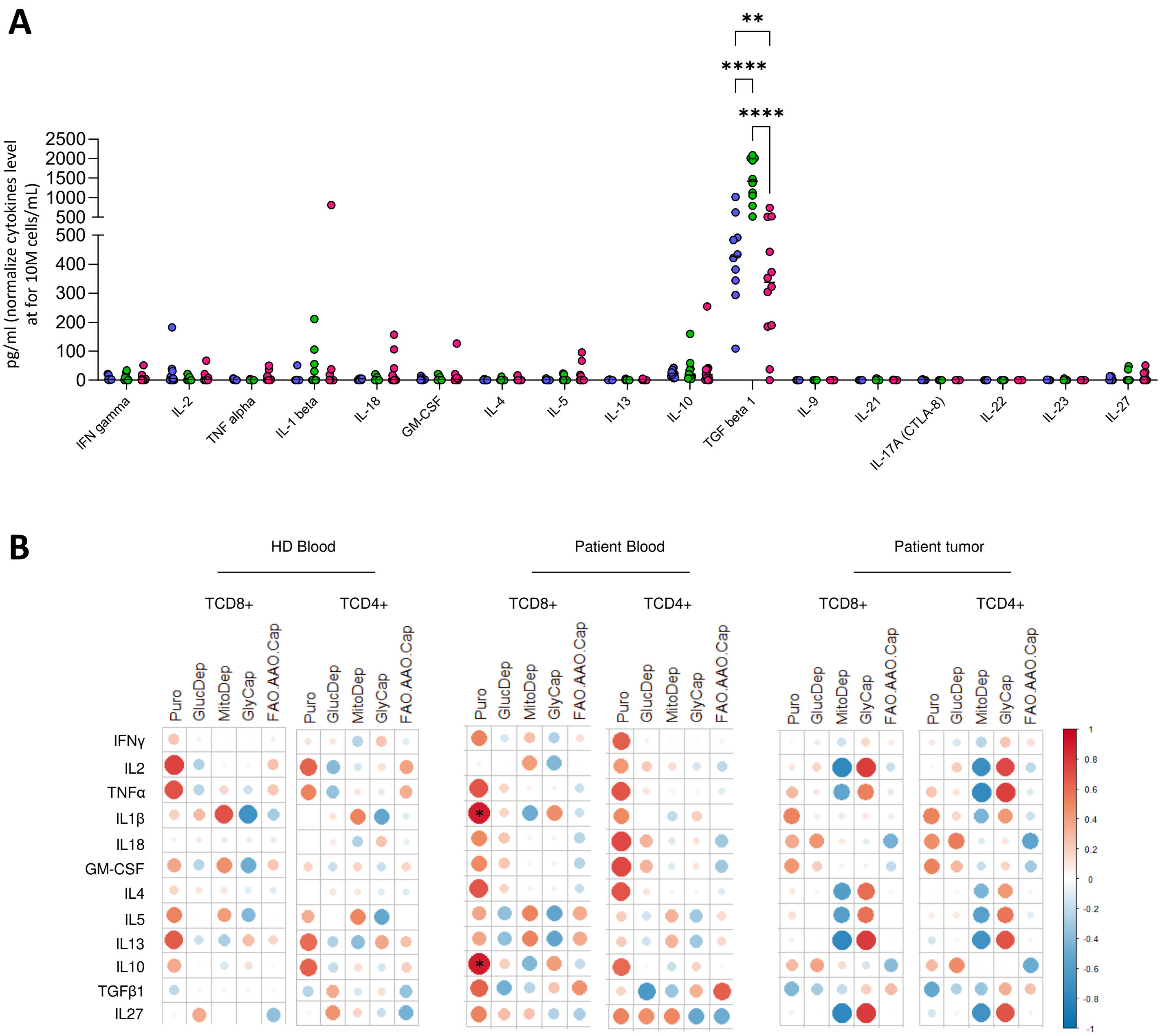

Supplementary Figure 8

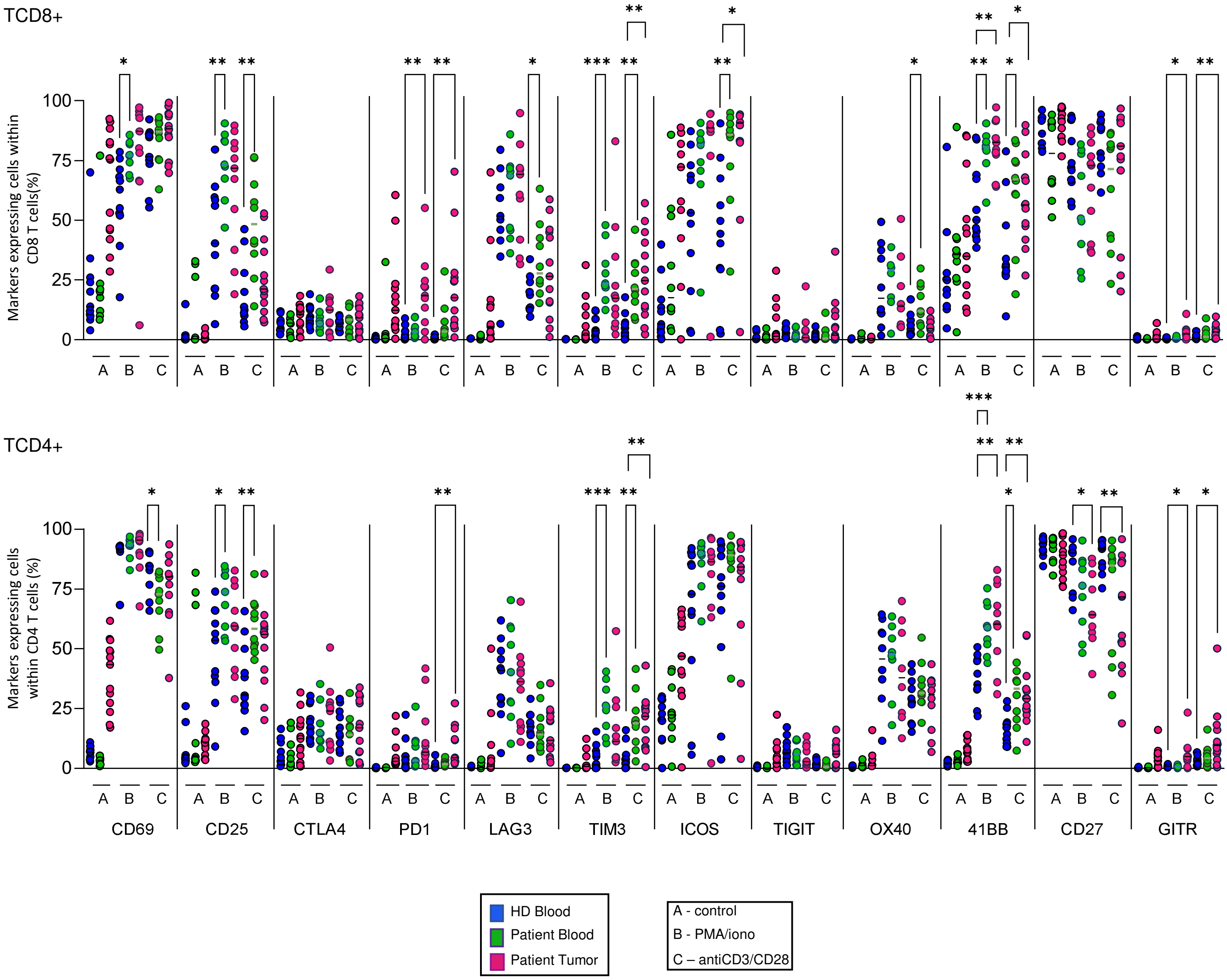

Supplementary Figure 9

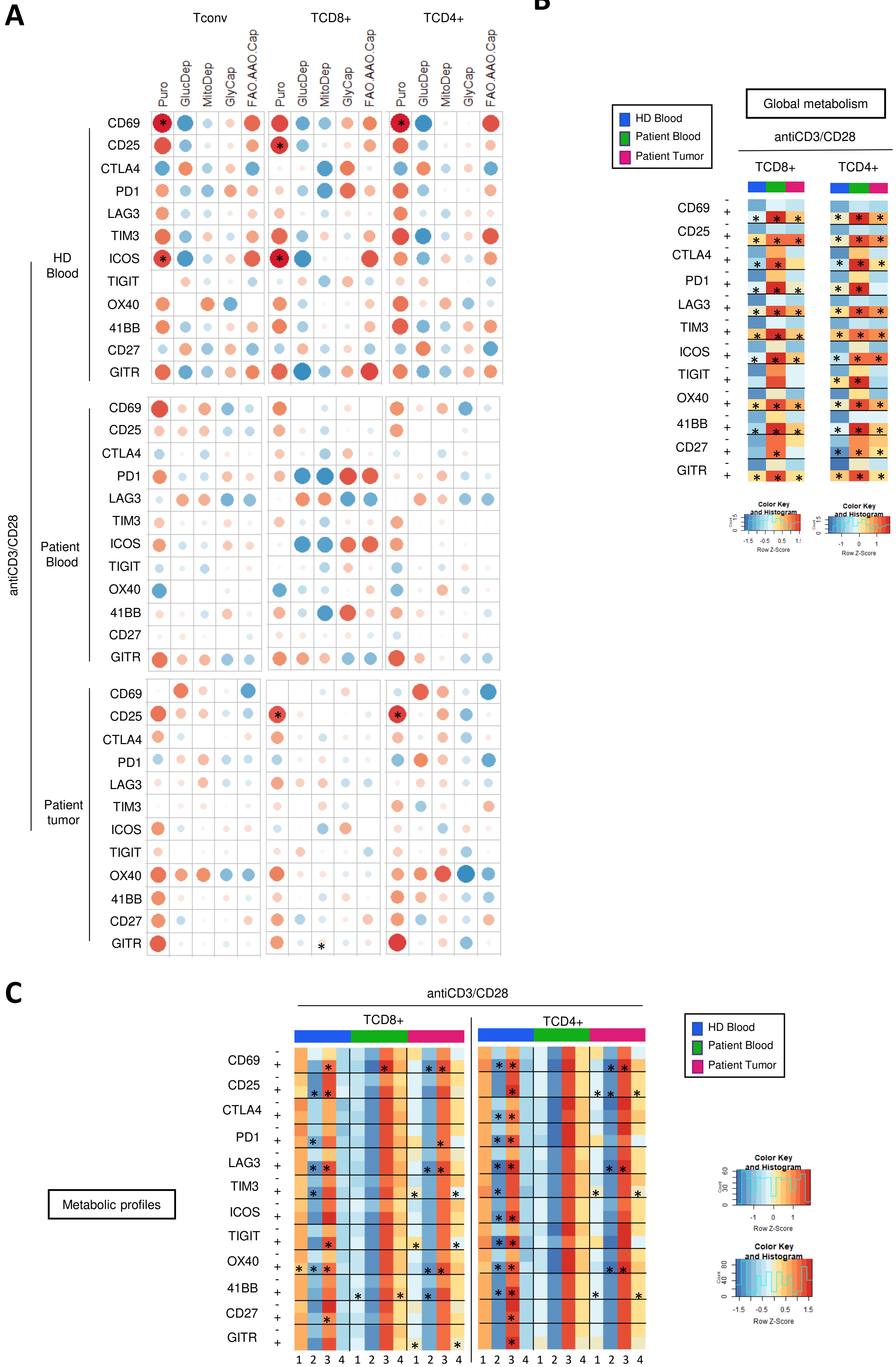

Supplementary Figure 9 (followed)

D

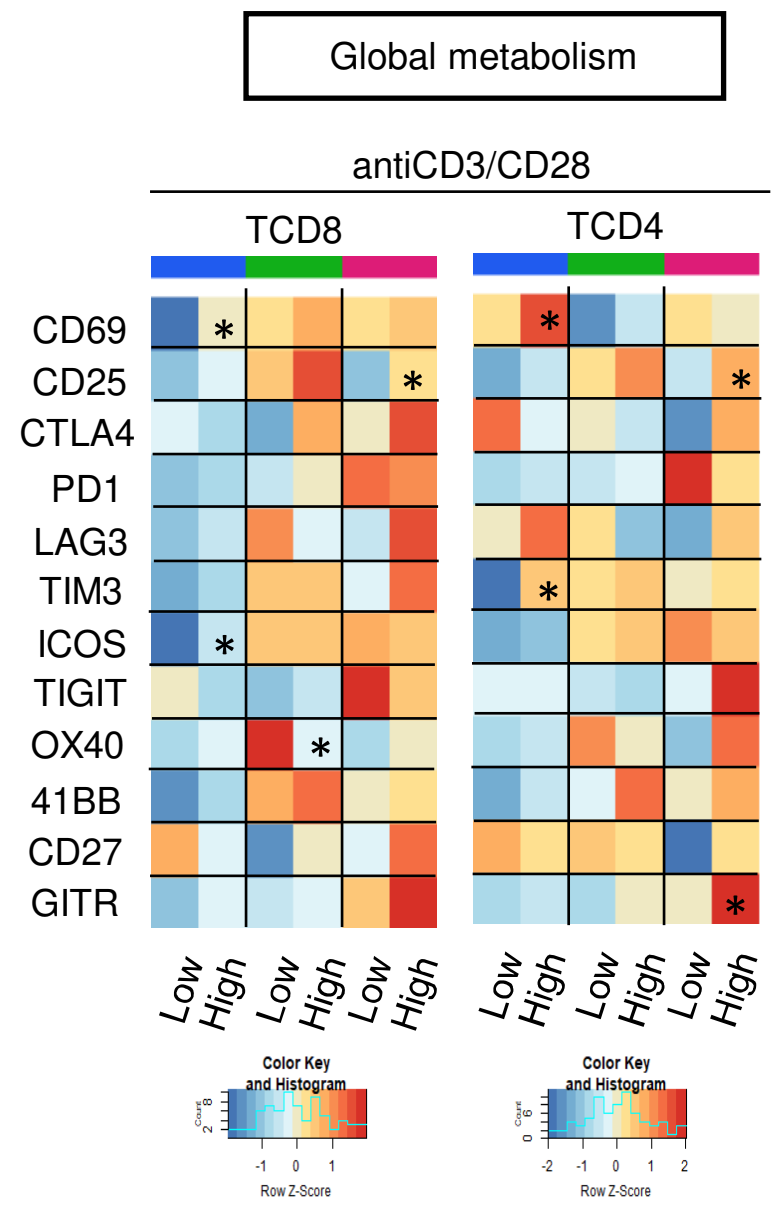

E

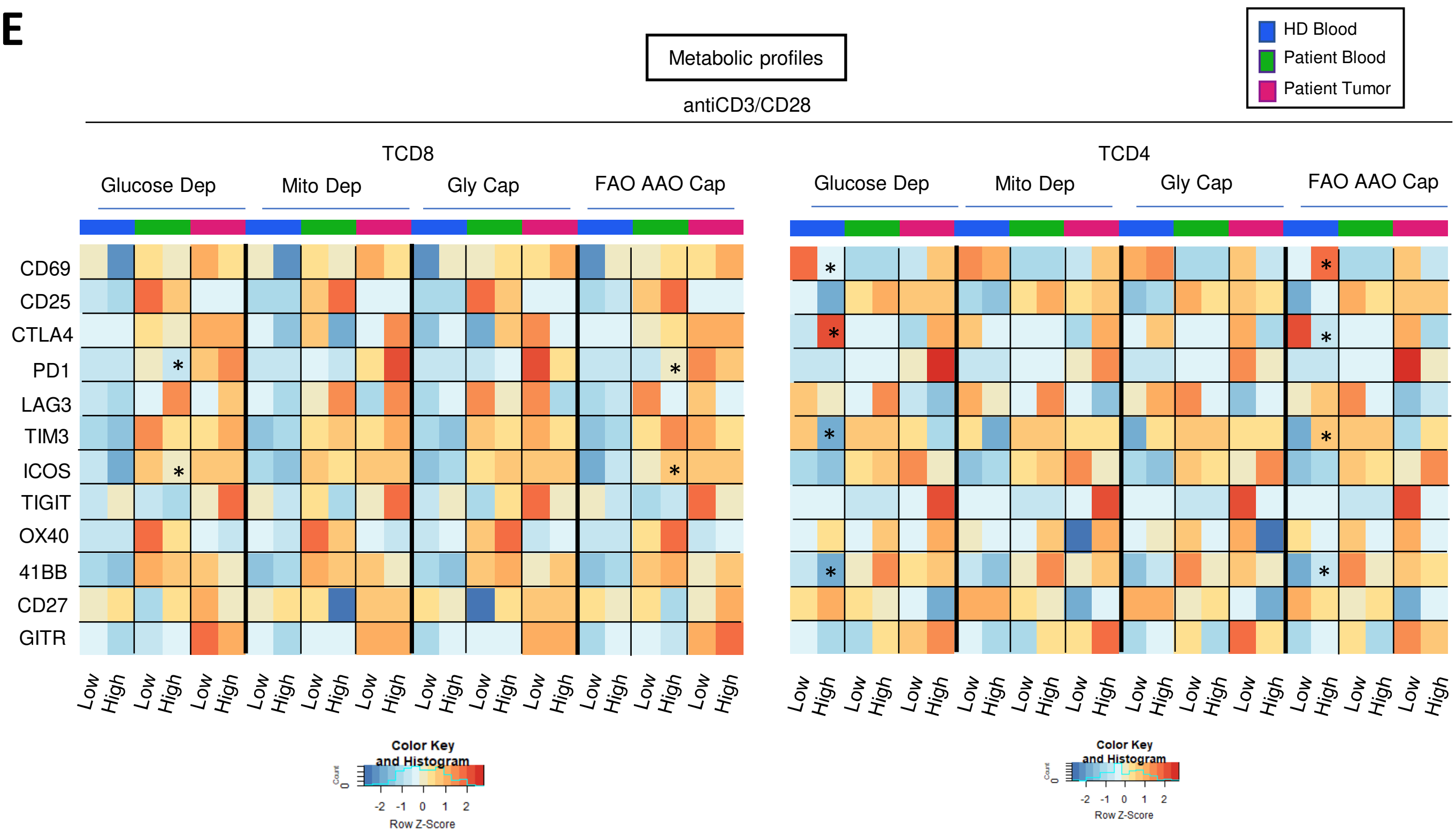

Supplementary Figure 10

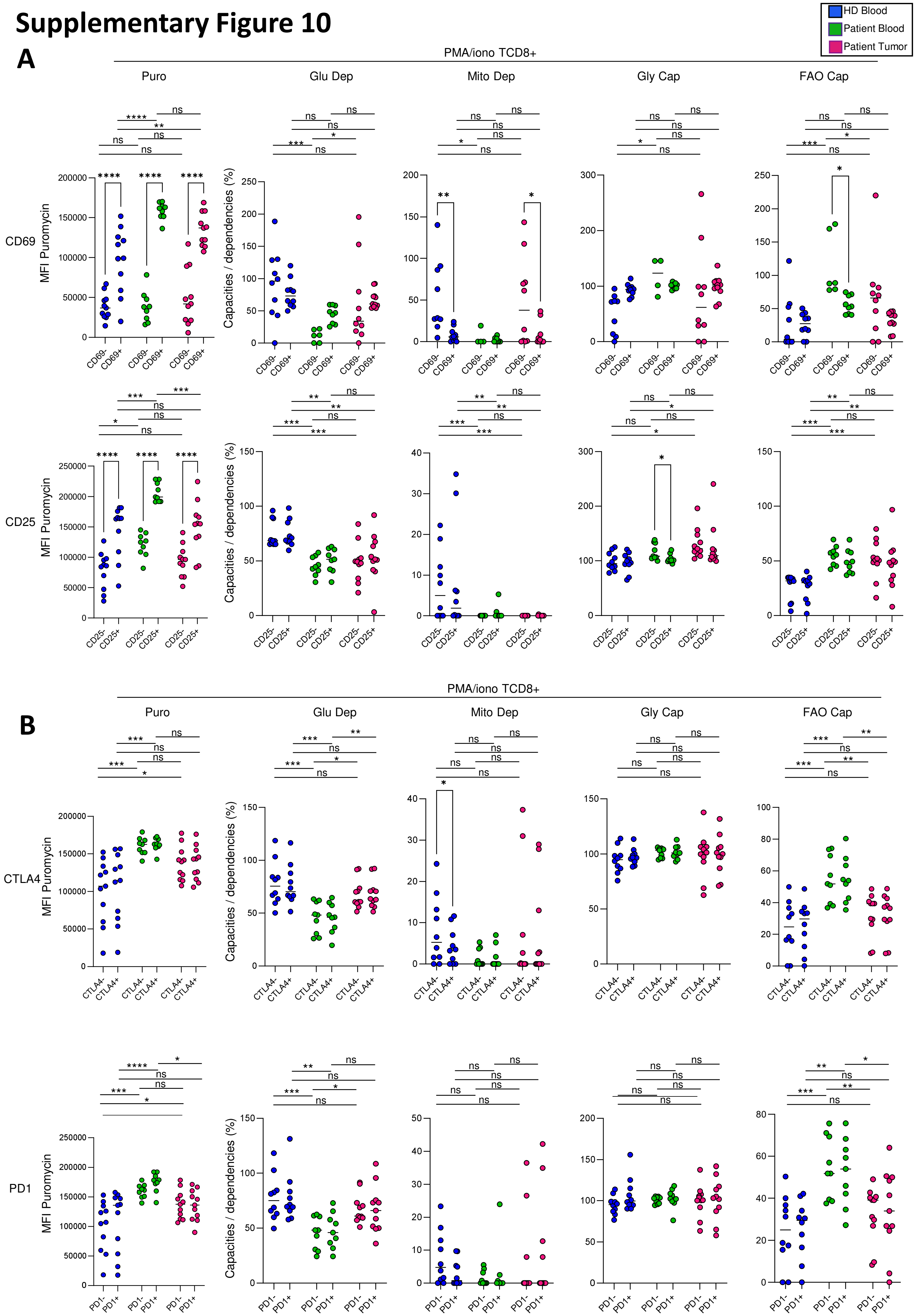

Supplementary Figure 10 (following)

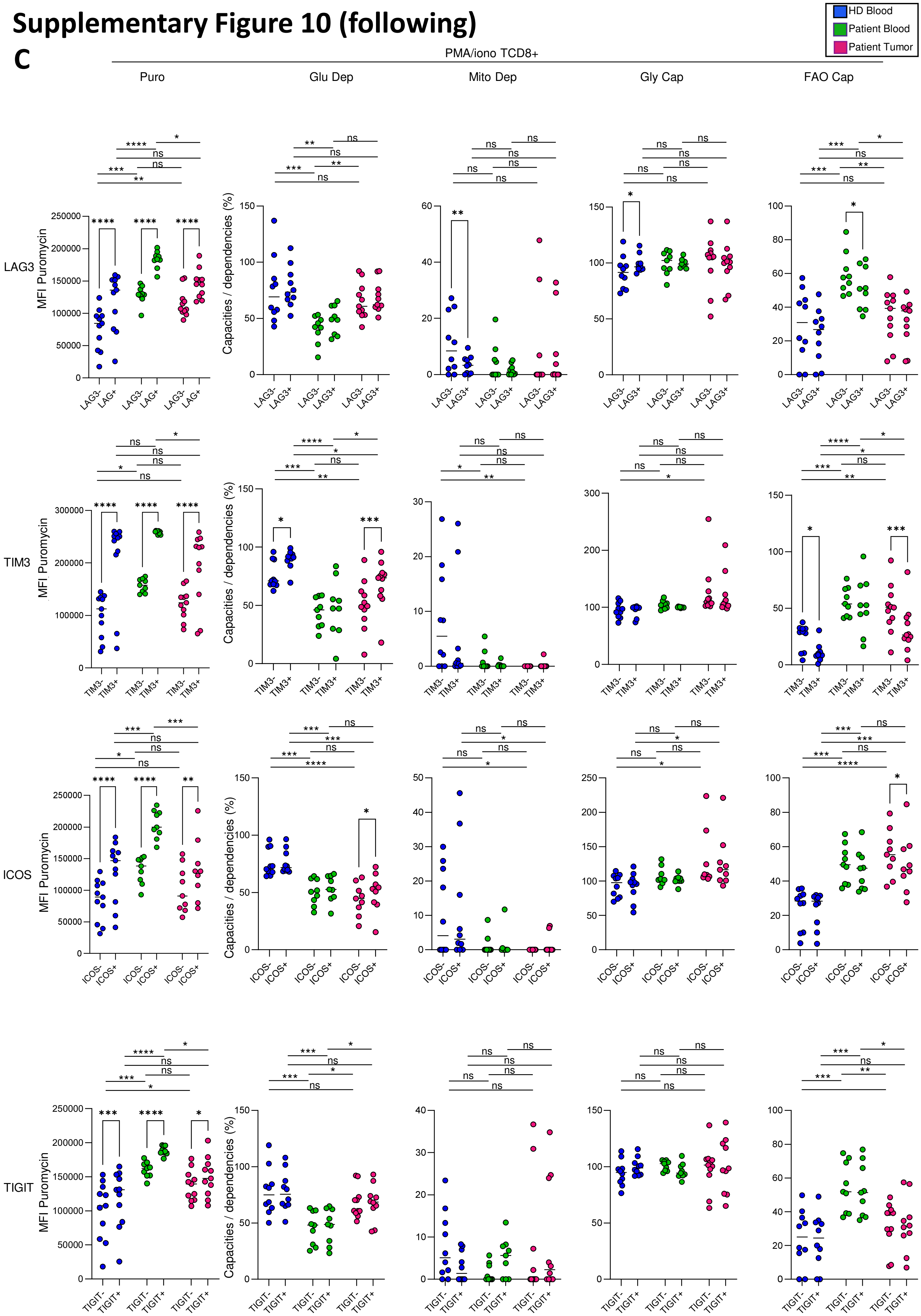

Supplementary Figure 10 (following)

D

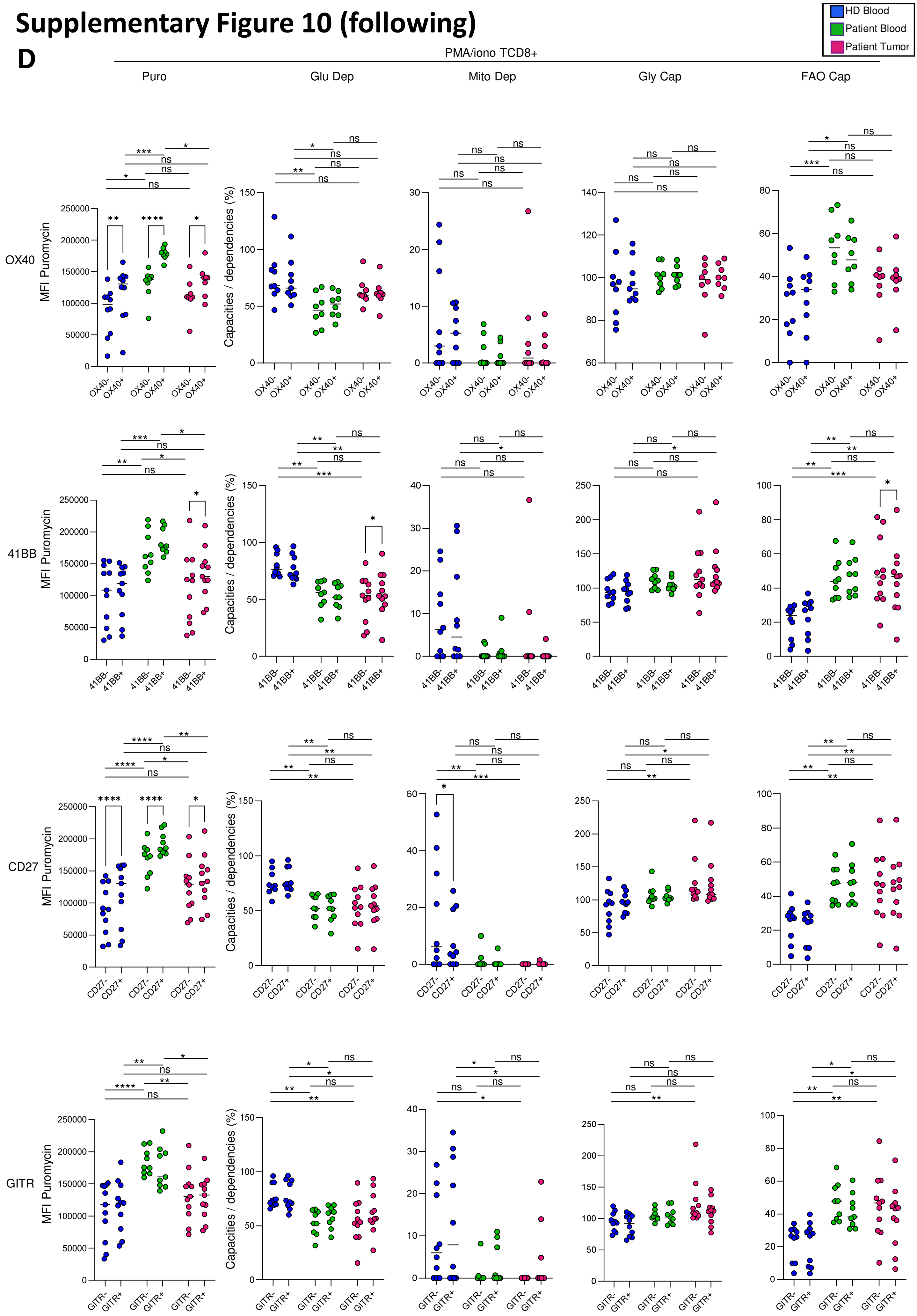

Supplementary Figure 11

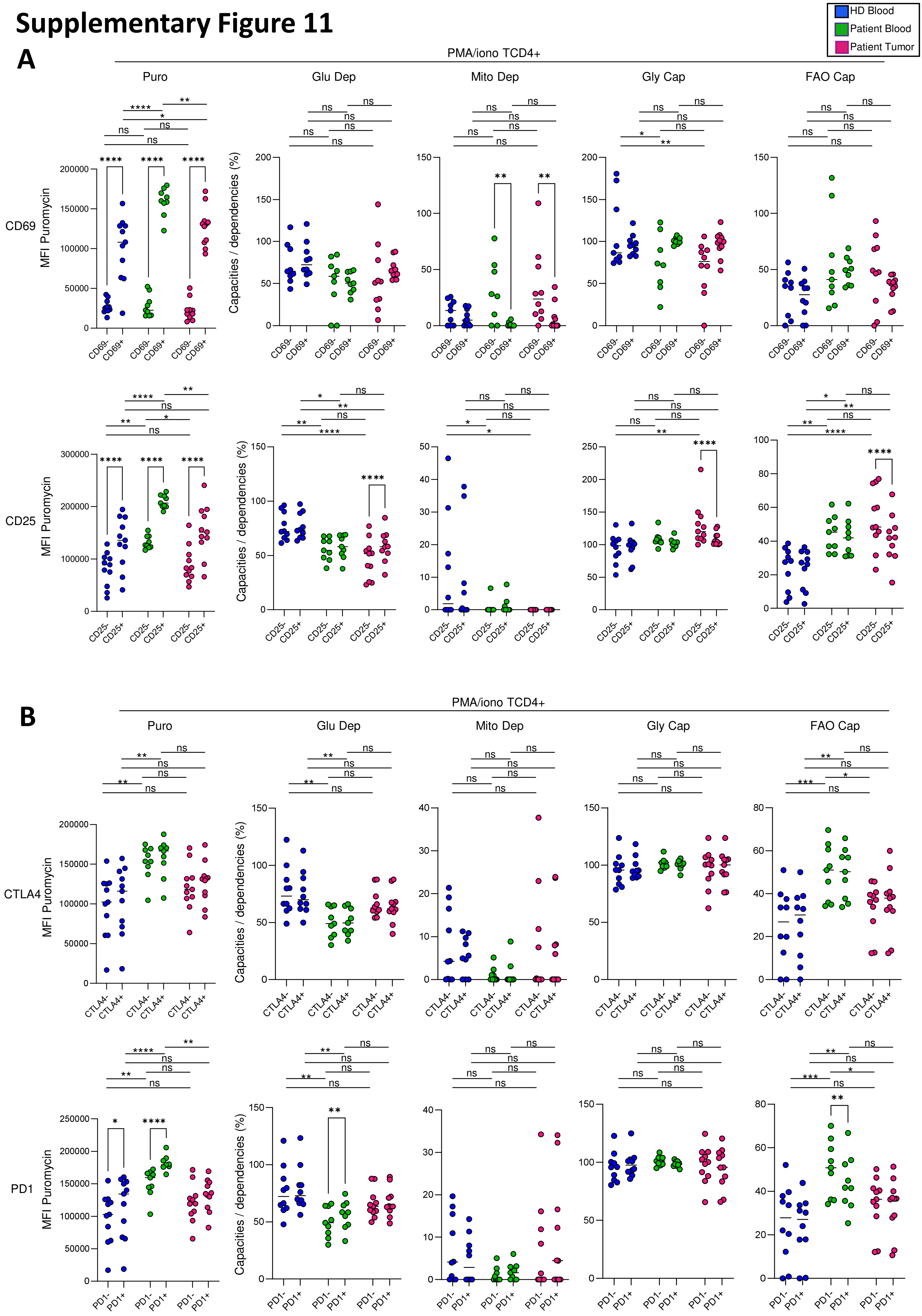

Supplementary Figure 11 (following)

C

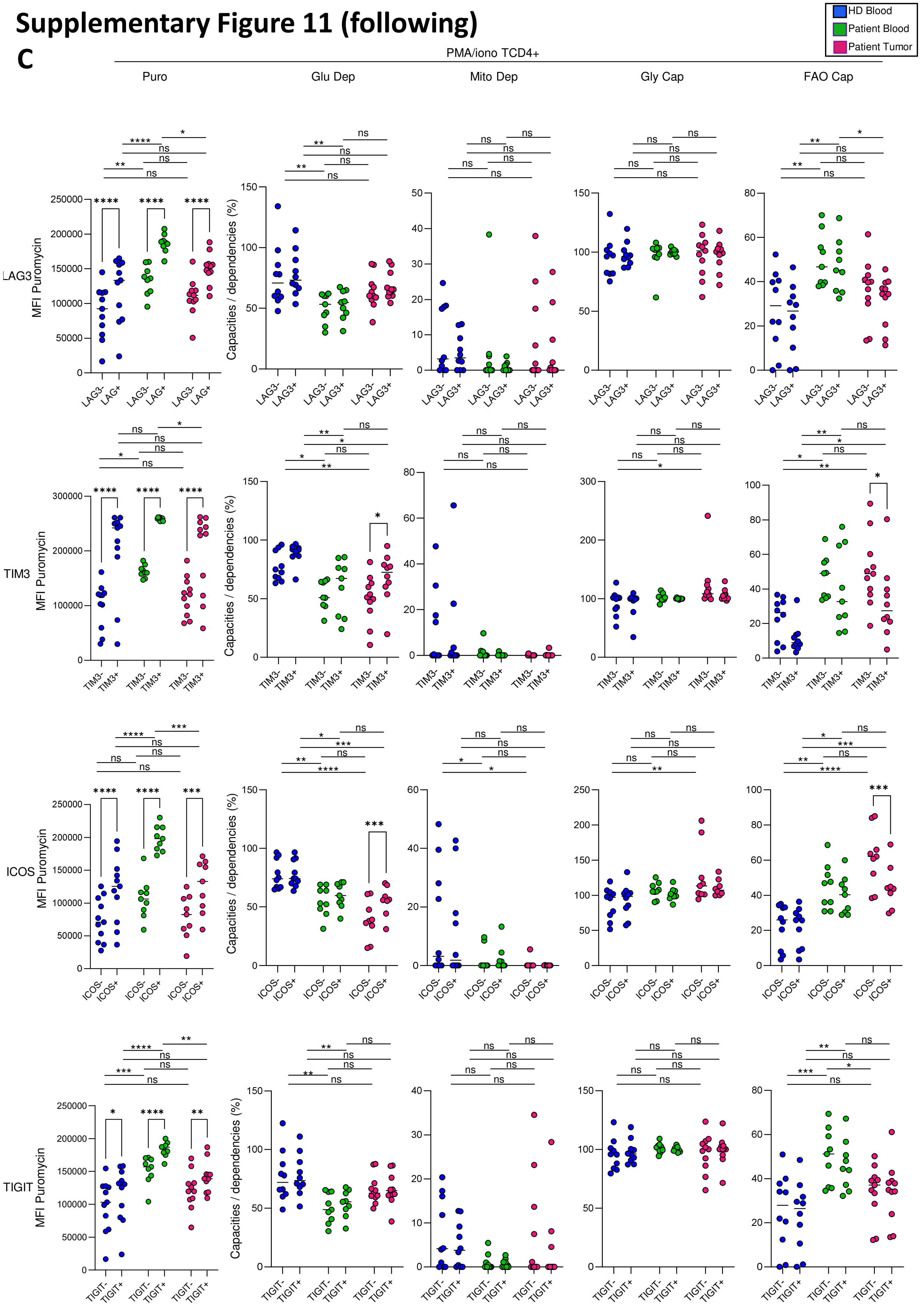

Supplementary Figure 11 (following)

D

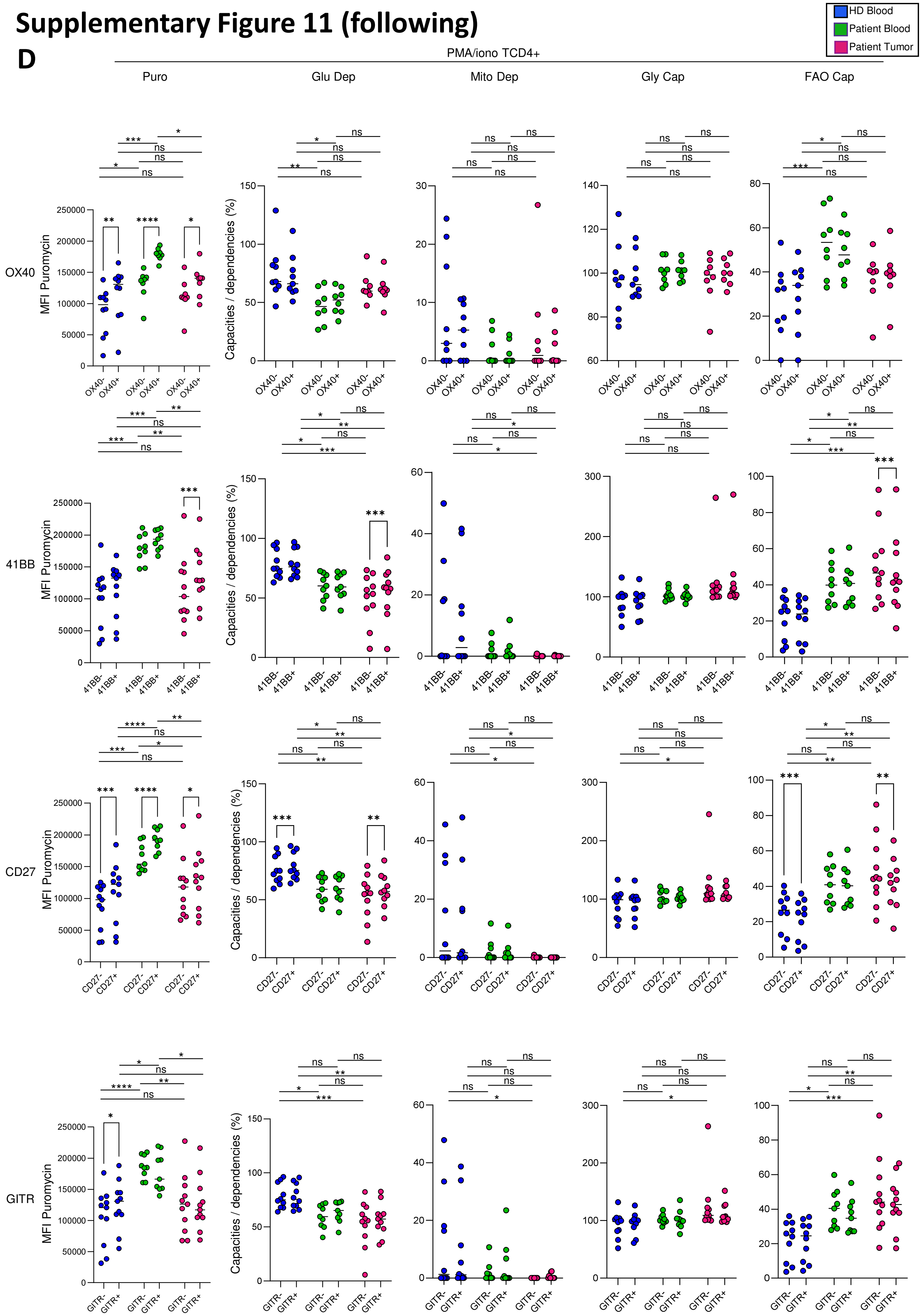

Supplementary Figure 12

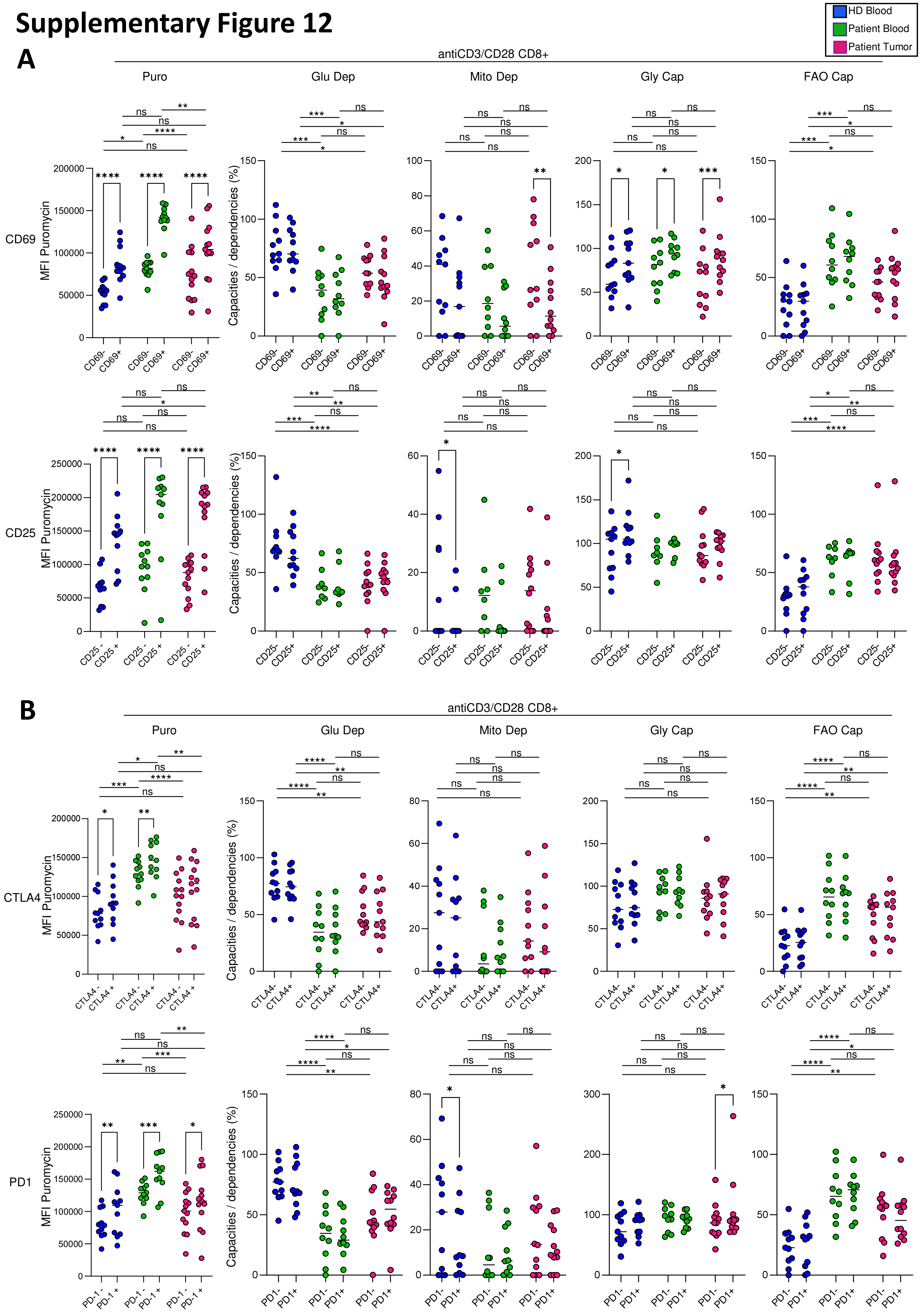

Supplementary Figure 12 (following)

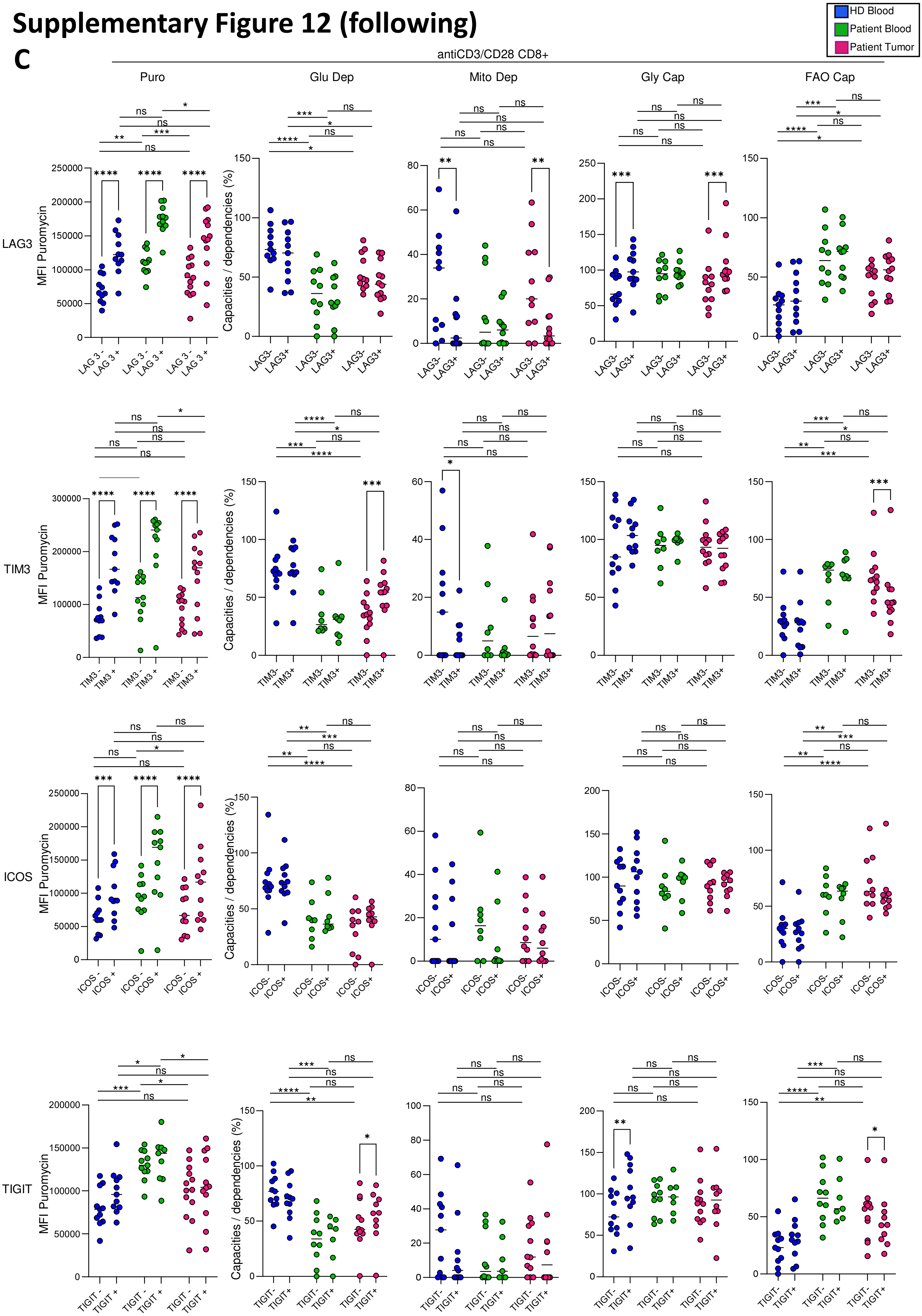

Supplementary Figure 12 (following)

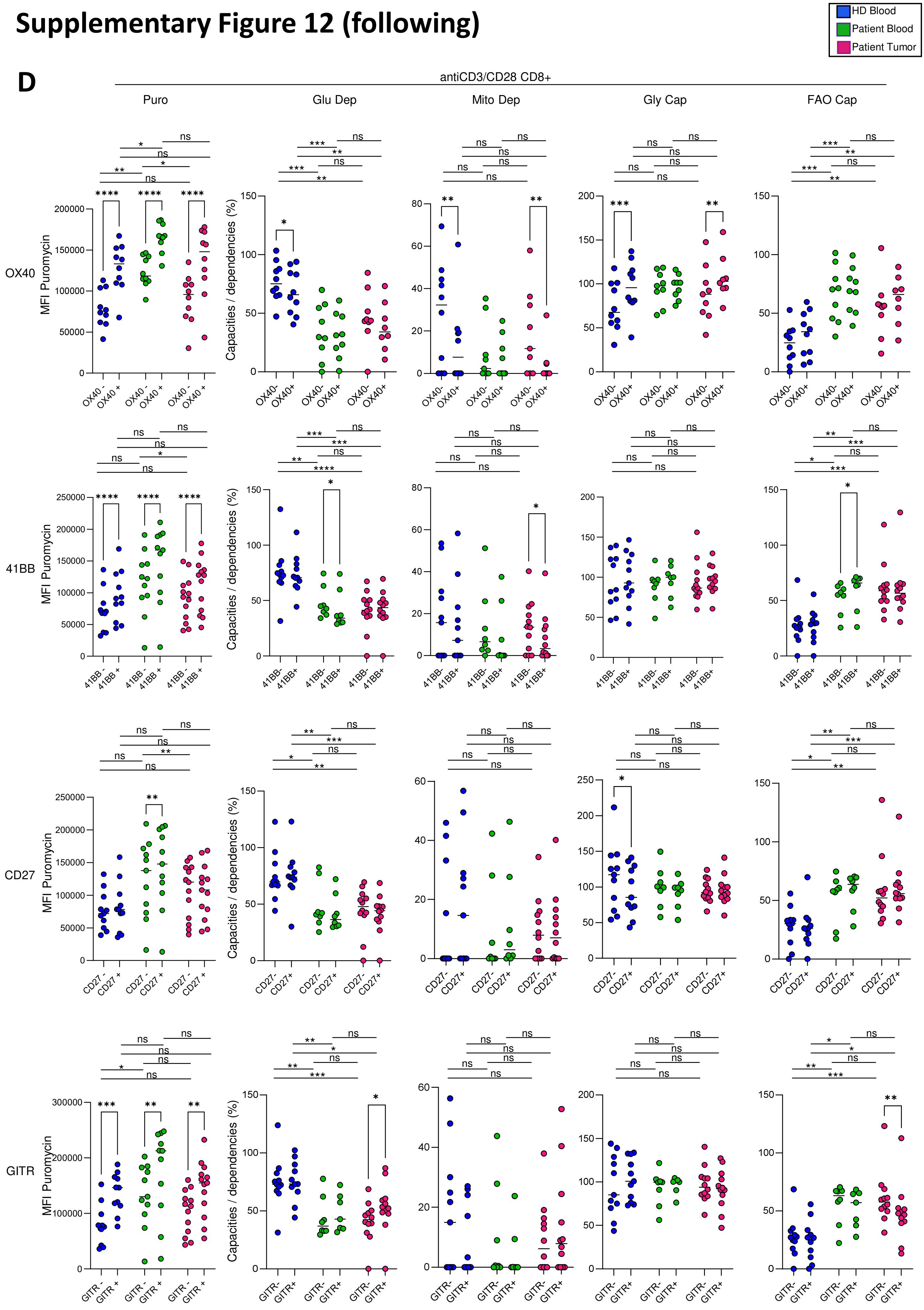

Supplementary Figure 13

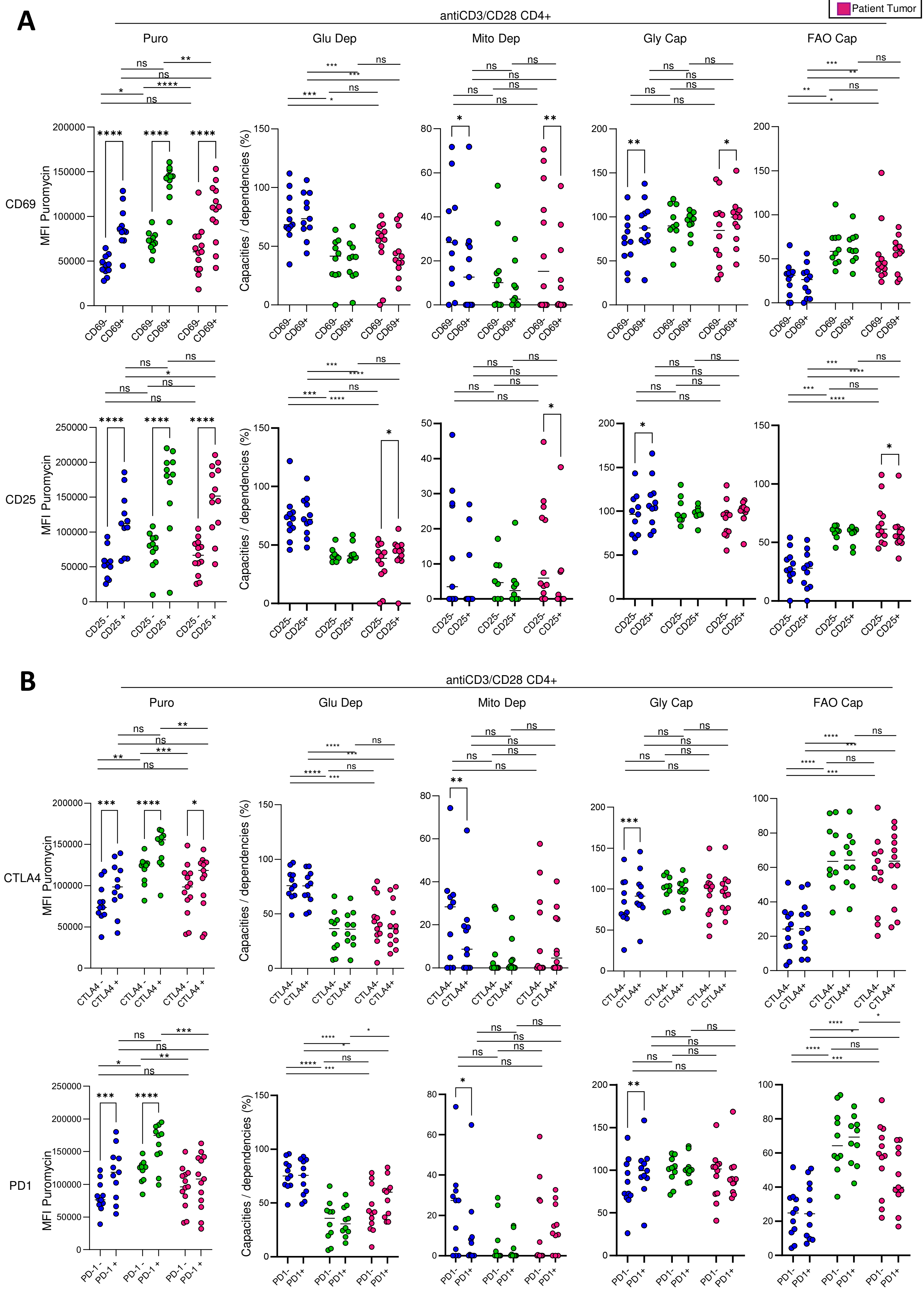

Supplementary Figure 13 (following)

C

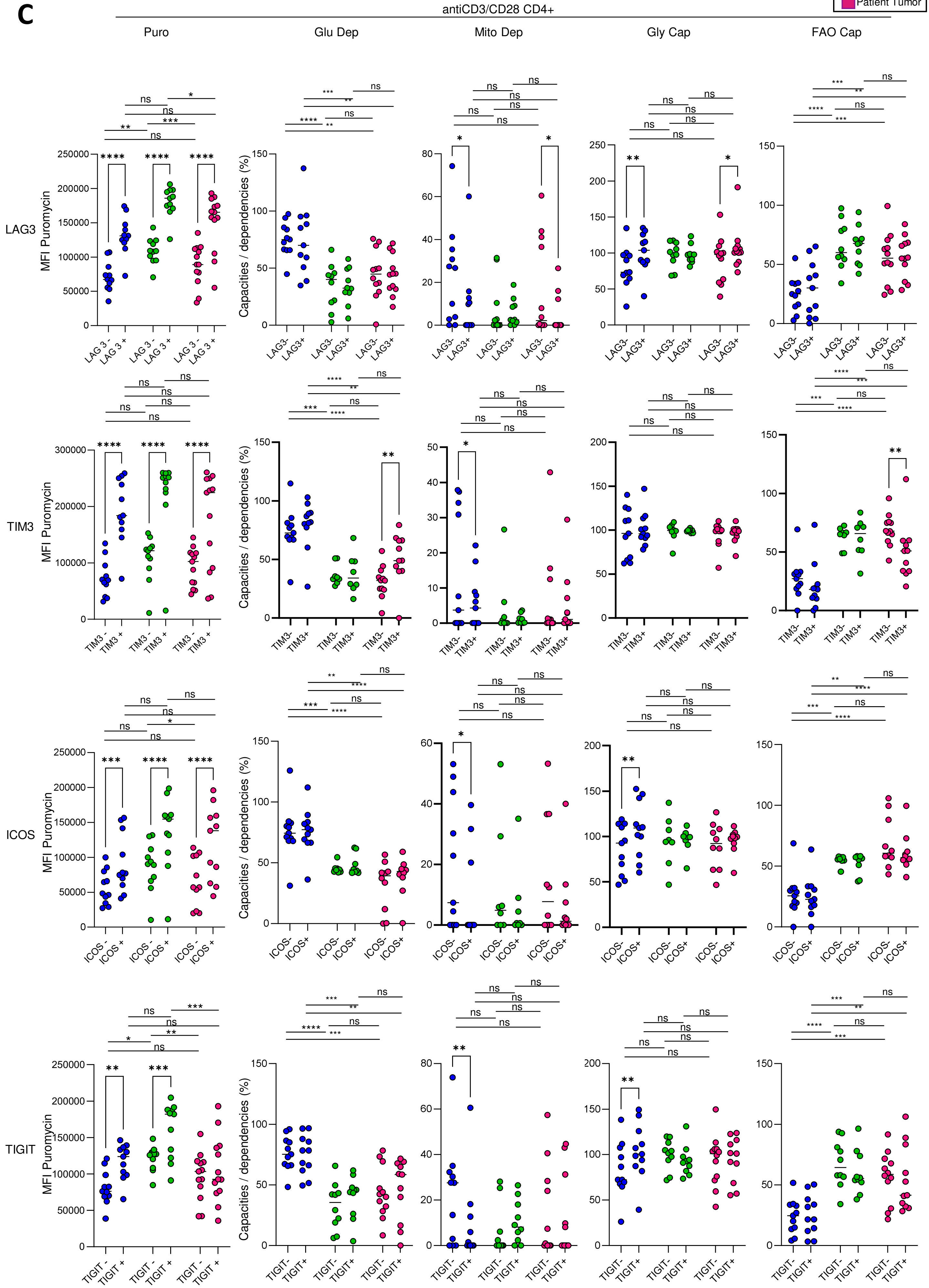

Supplementary Figure 13 (following)

D

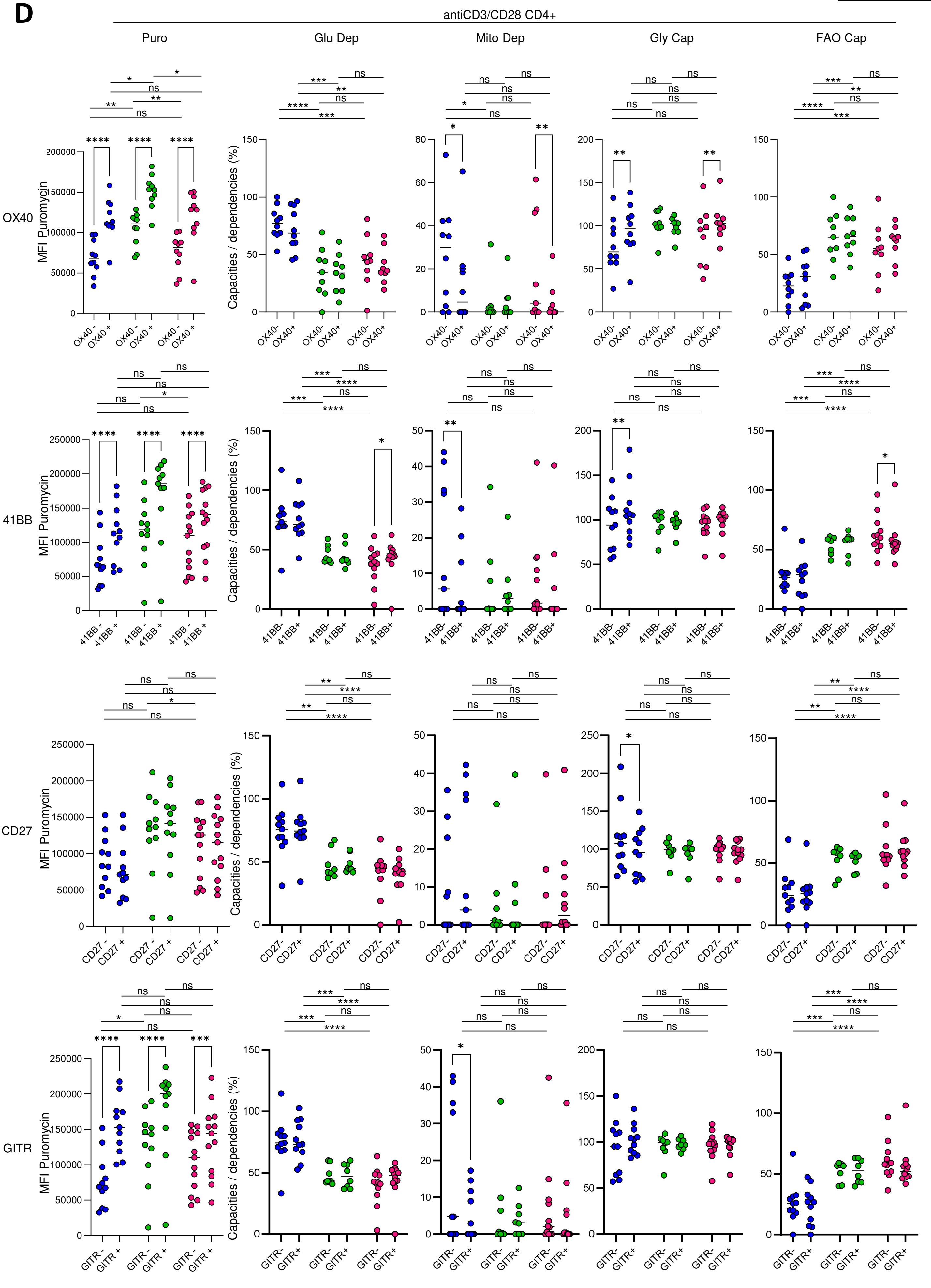

Supplementary Figure 14

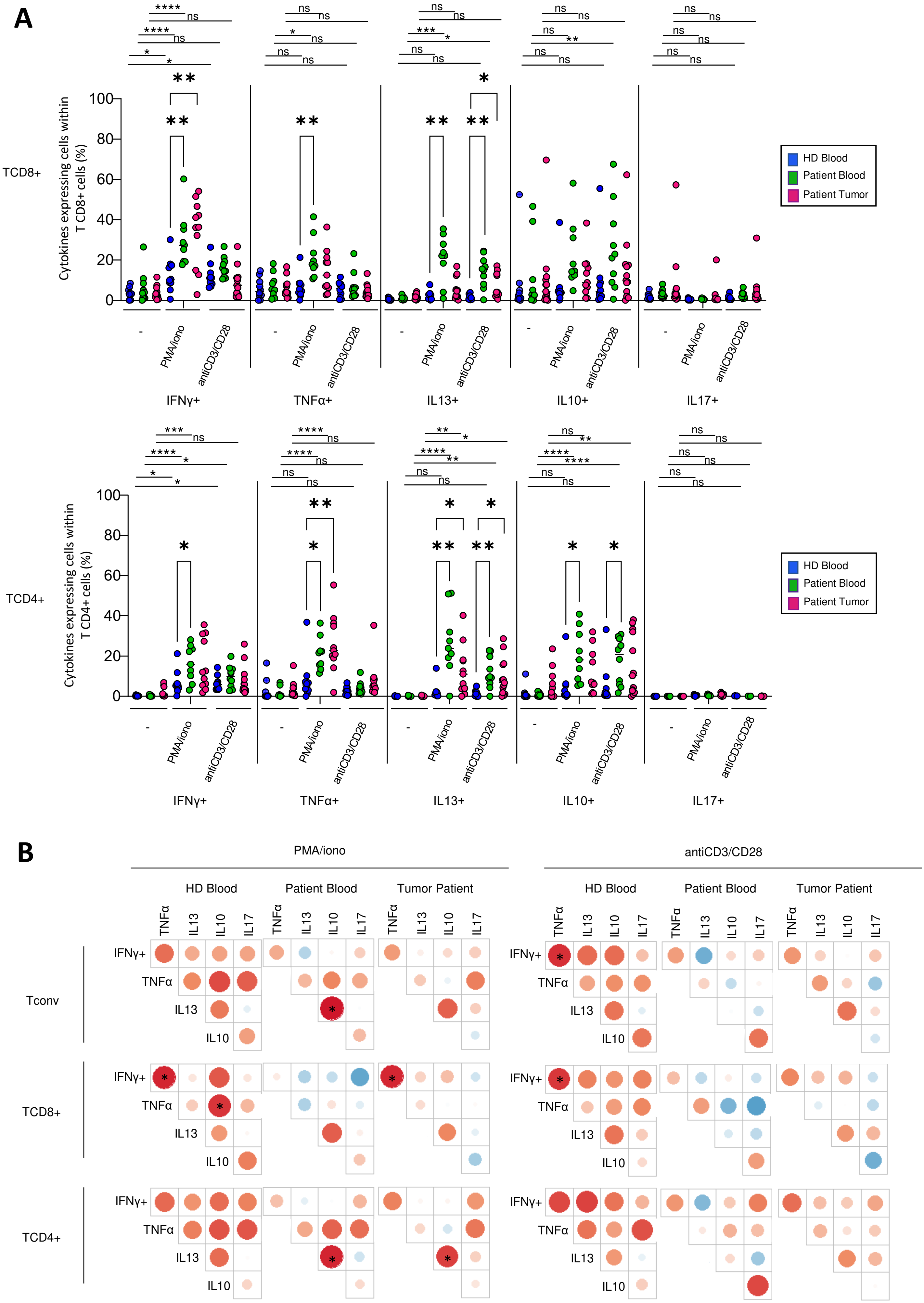

Supplementary Figure 15

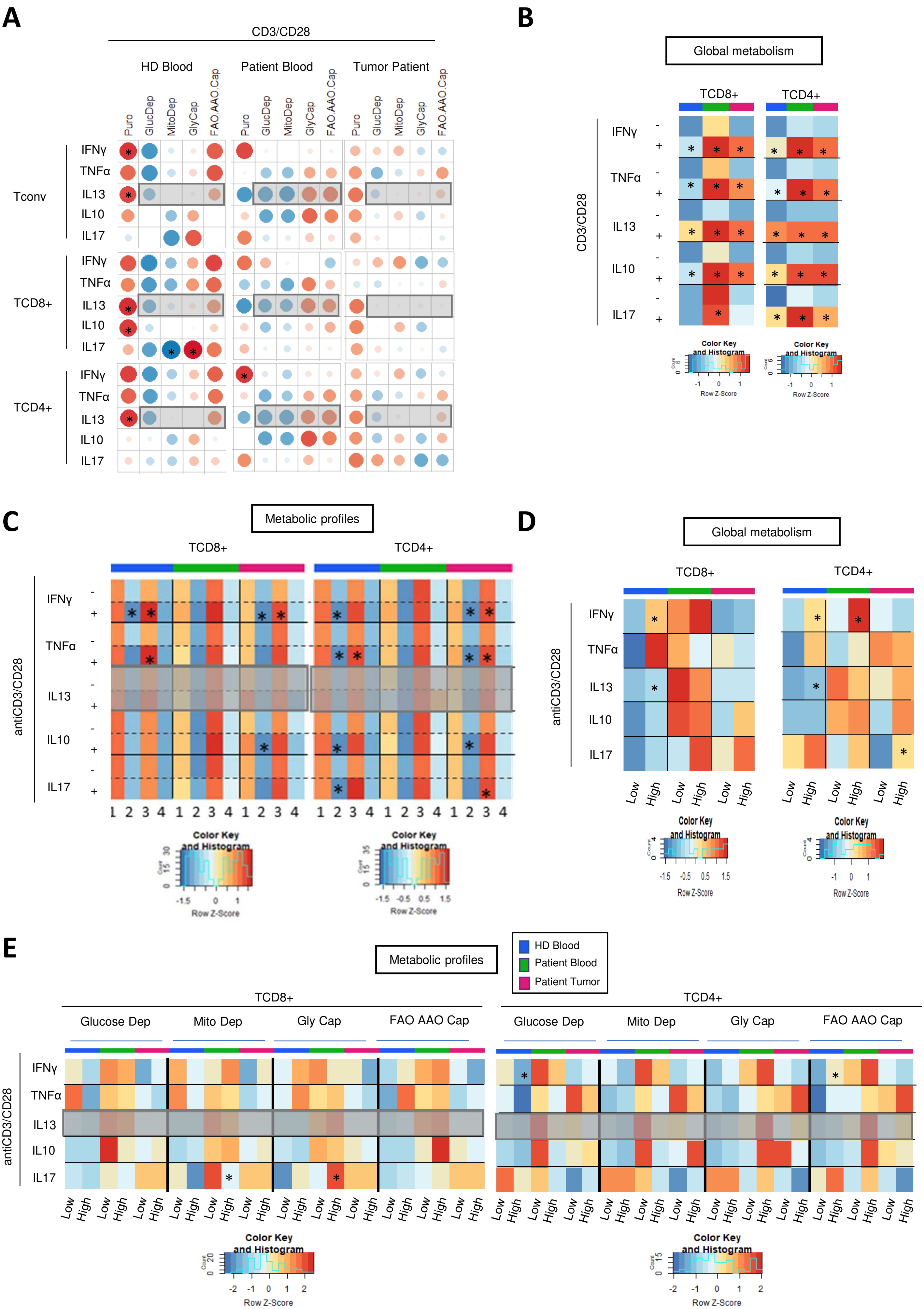

Supplementary Figure 16

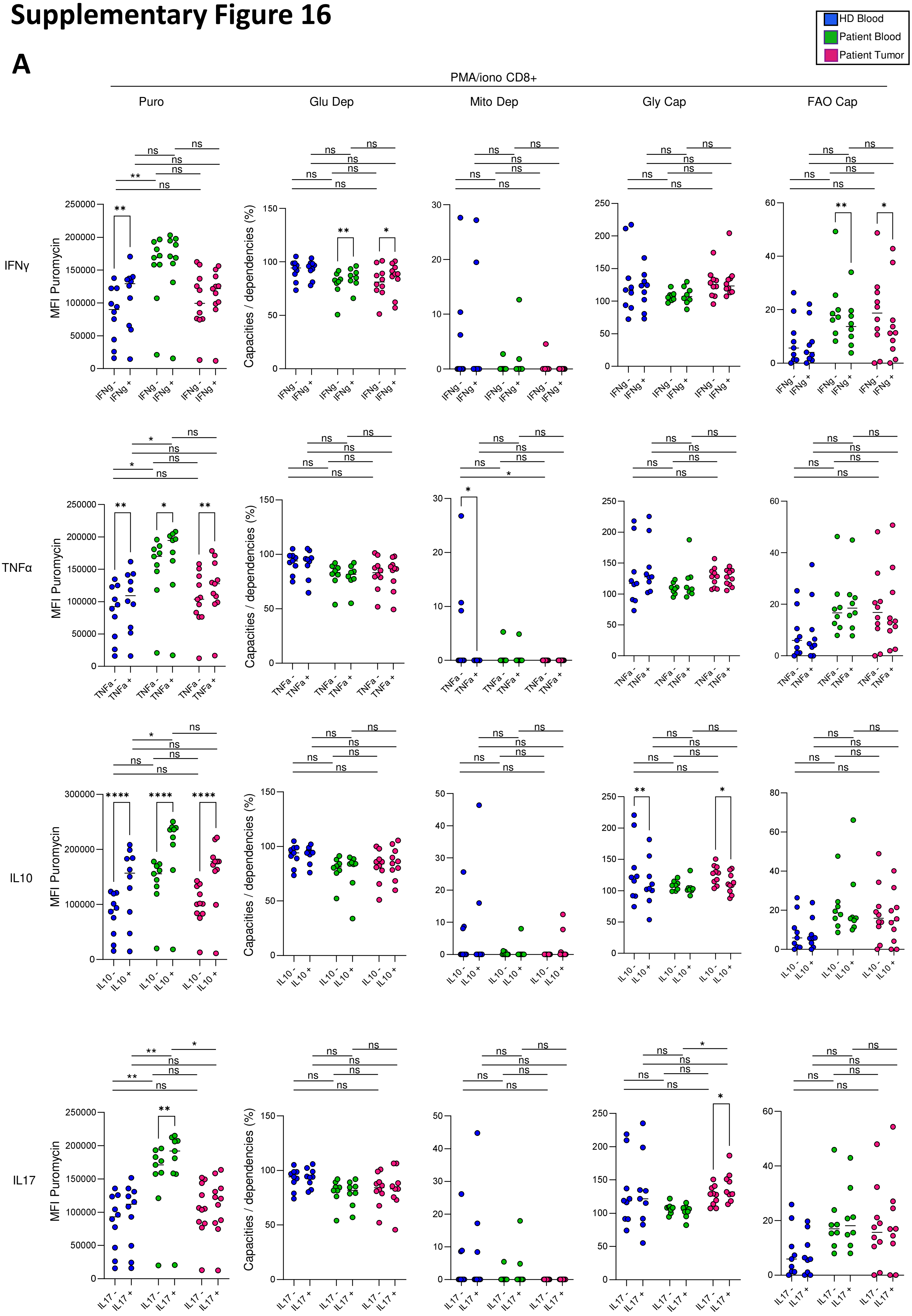

Supplementary Figure 16 (following)

B

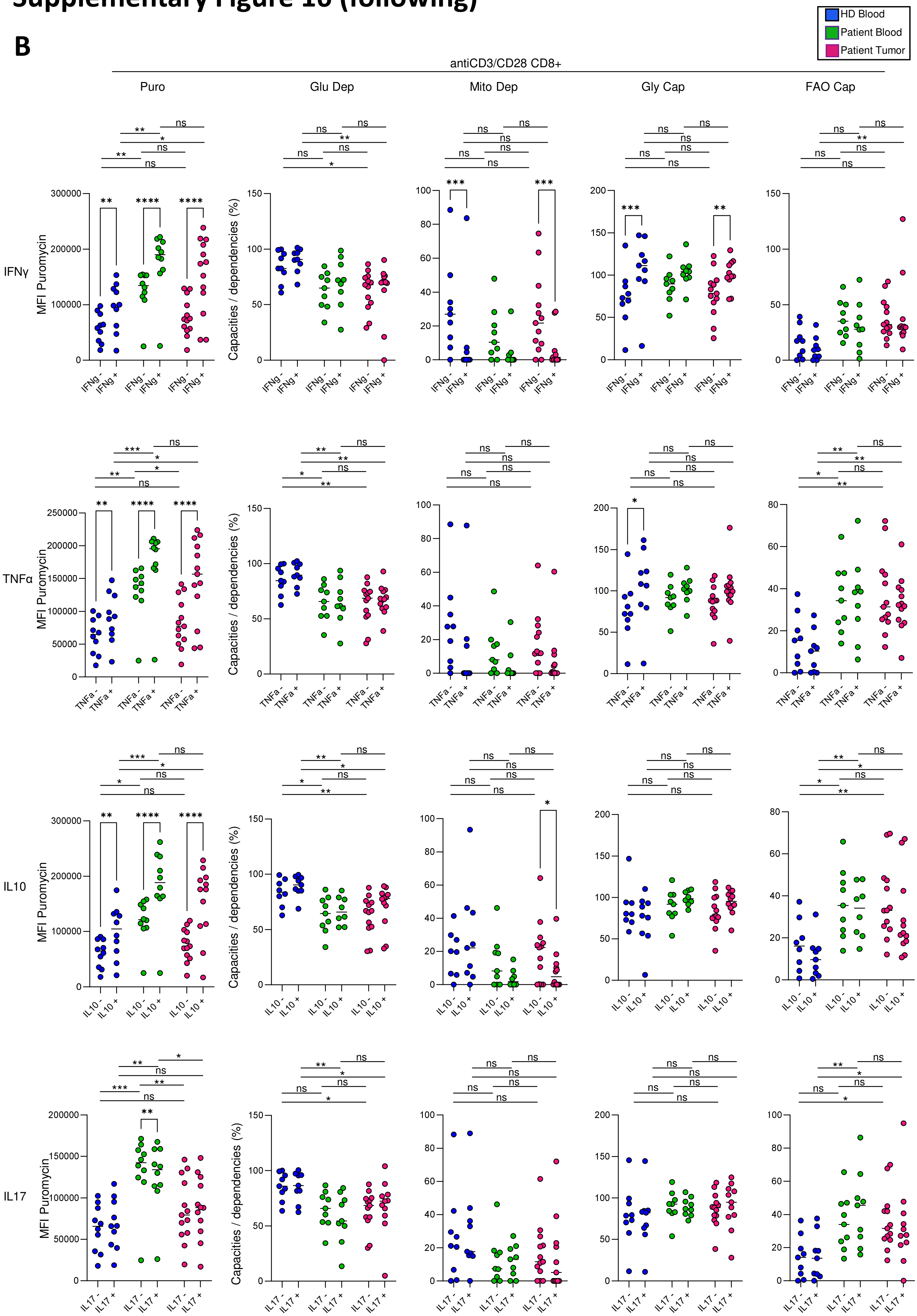

Supplementary Figure 17

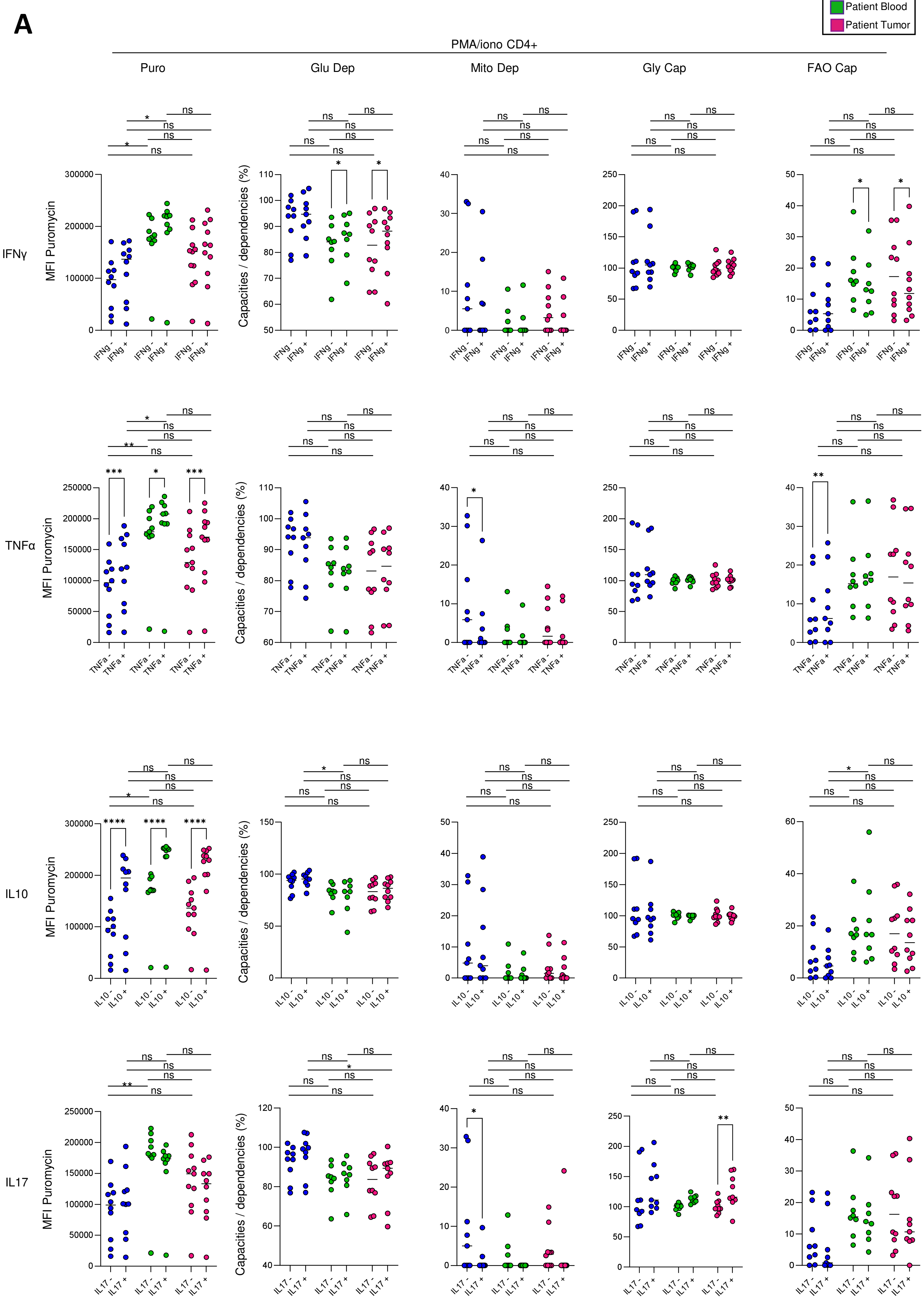

Supplementary Figure 17 (following)

B

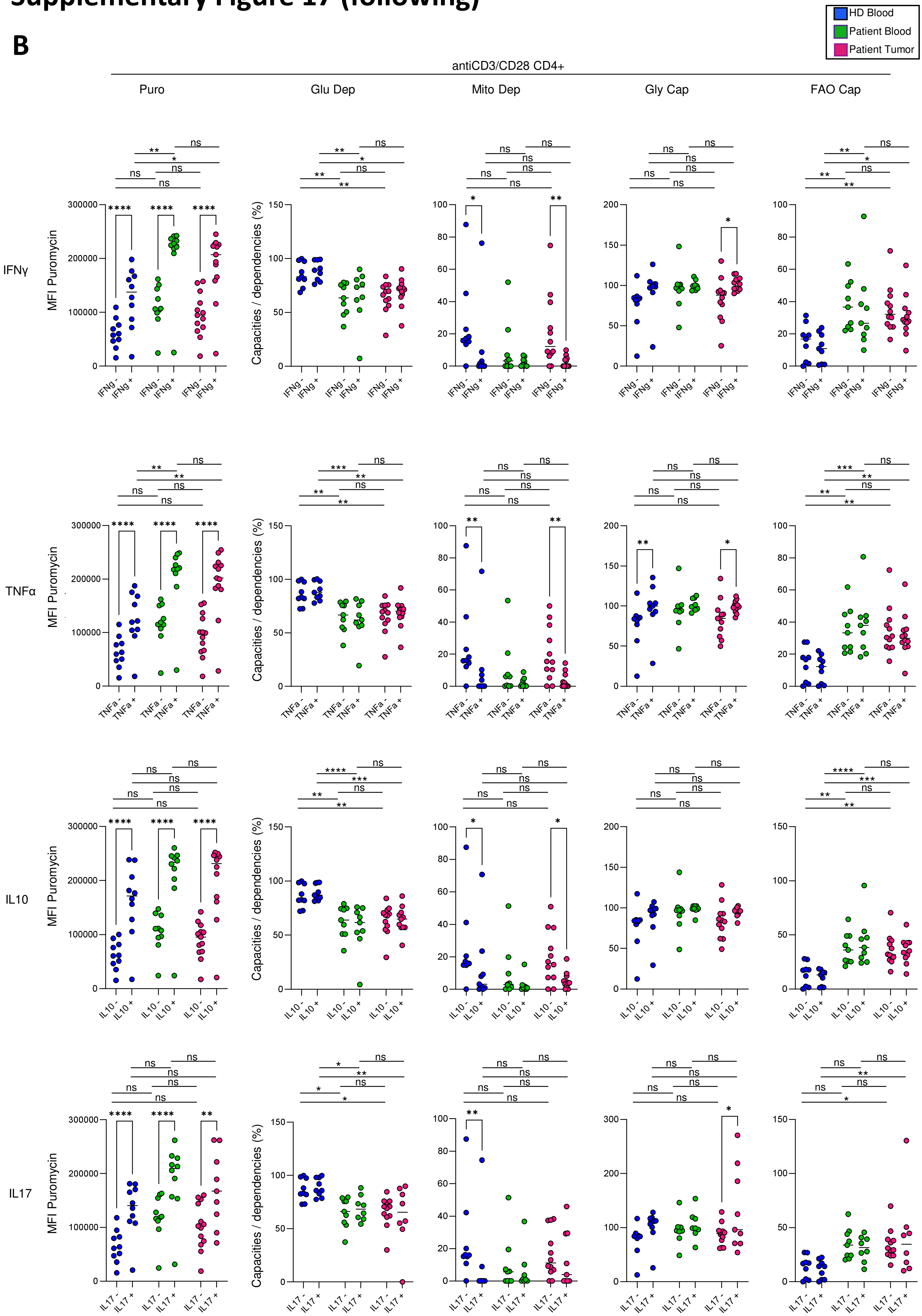

Supplementary Figure 18

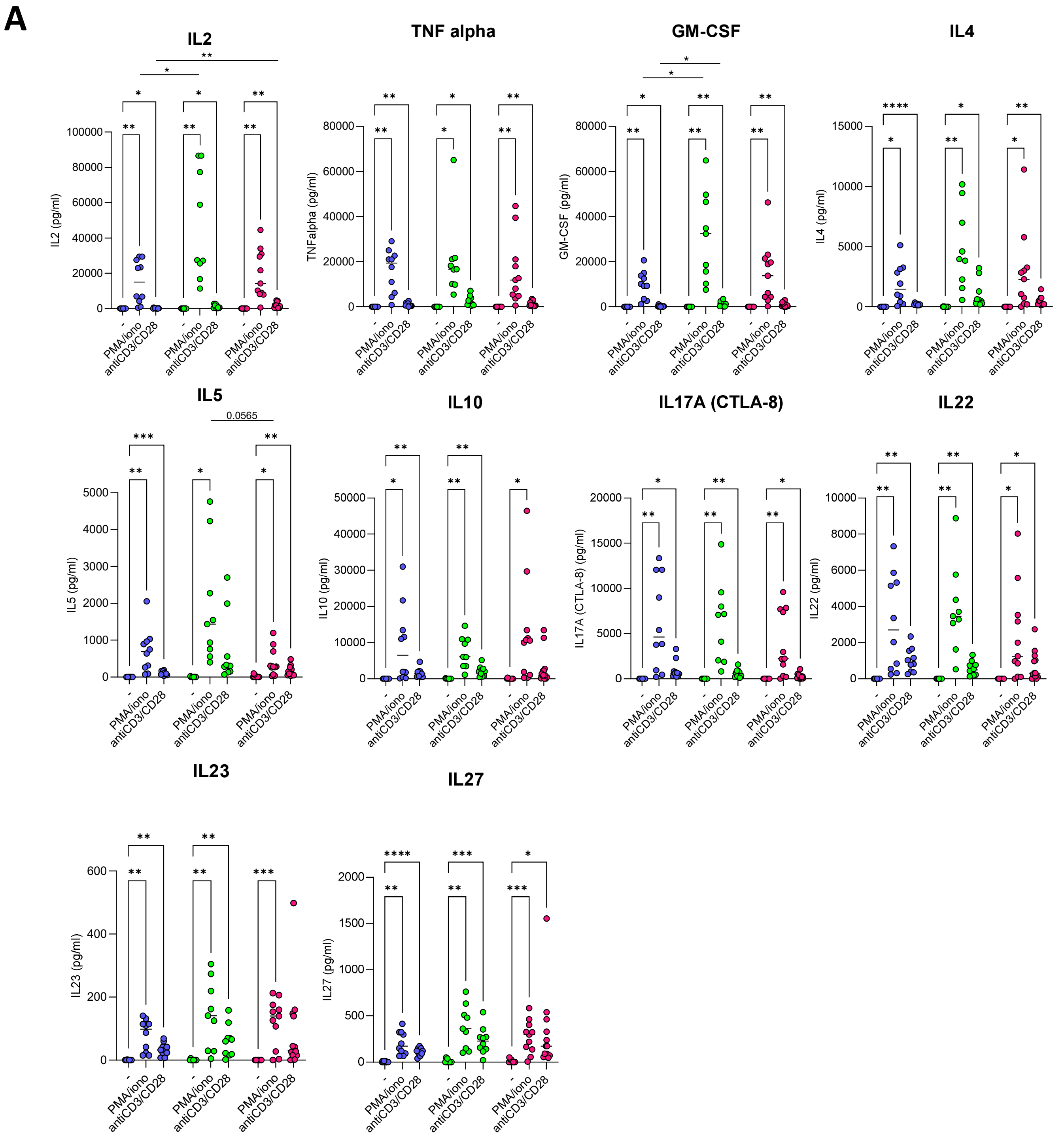

Supplementary Figure 18 (followed)

B

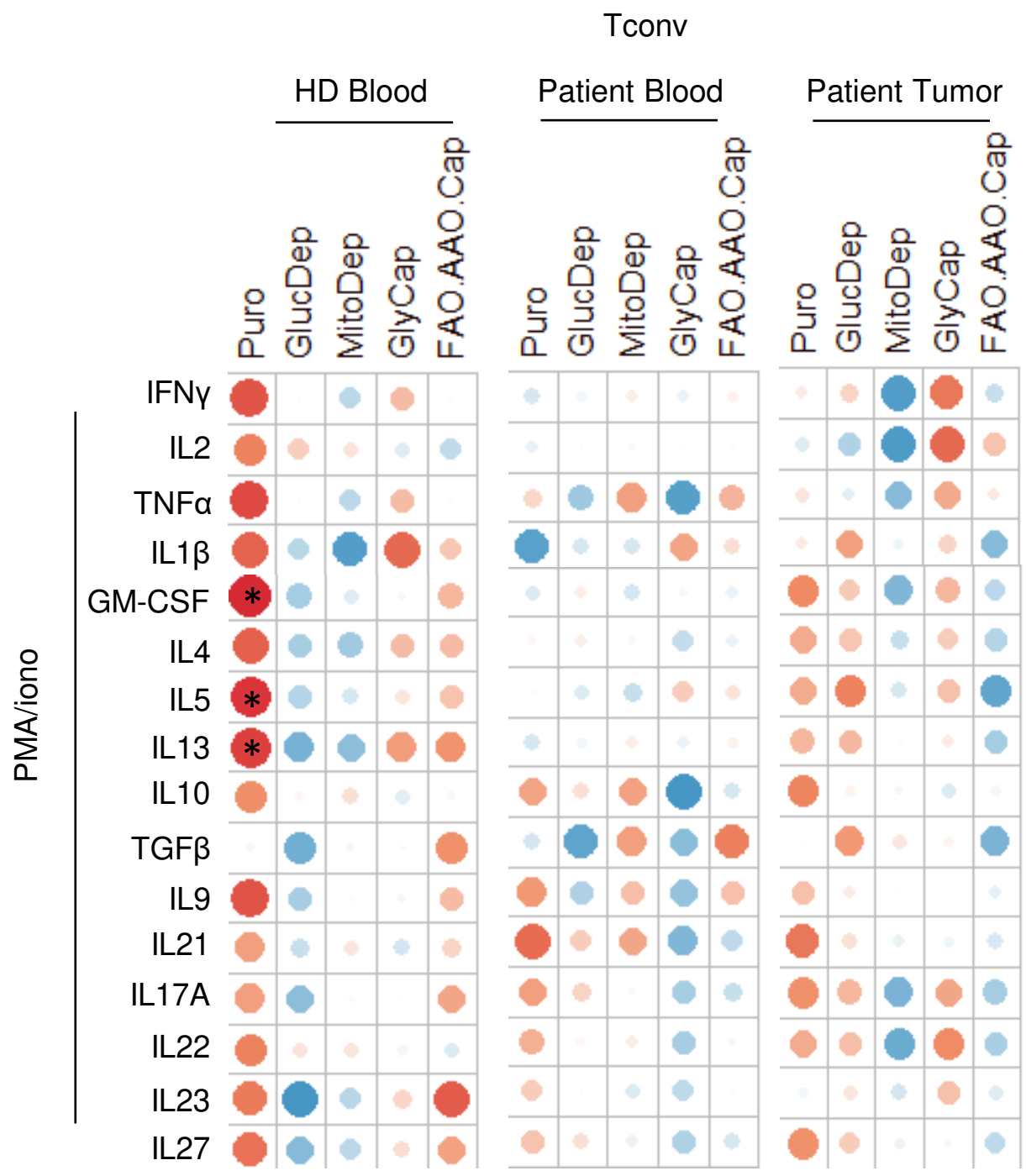

C

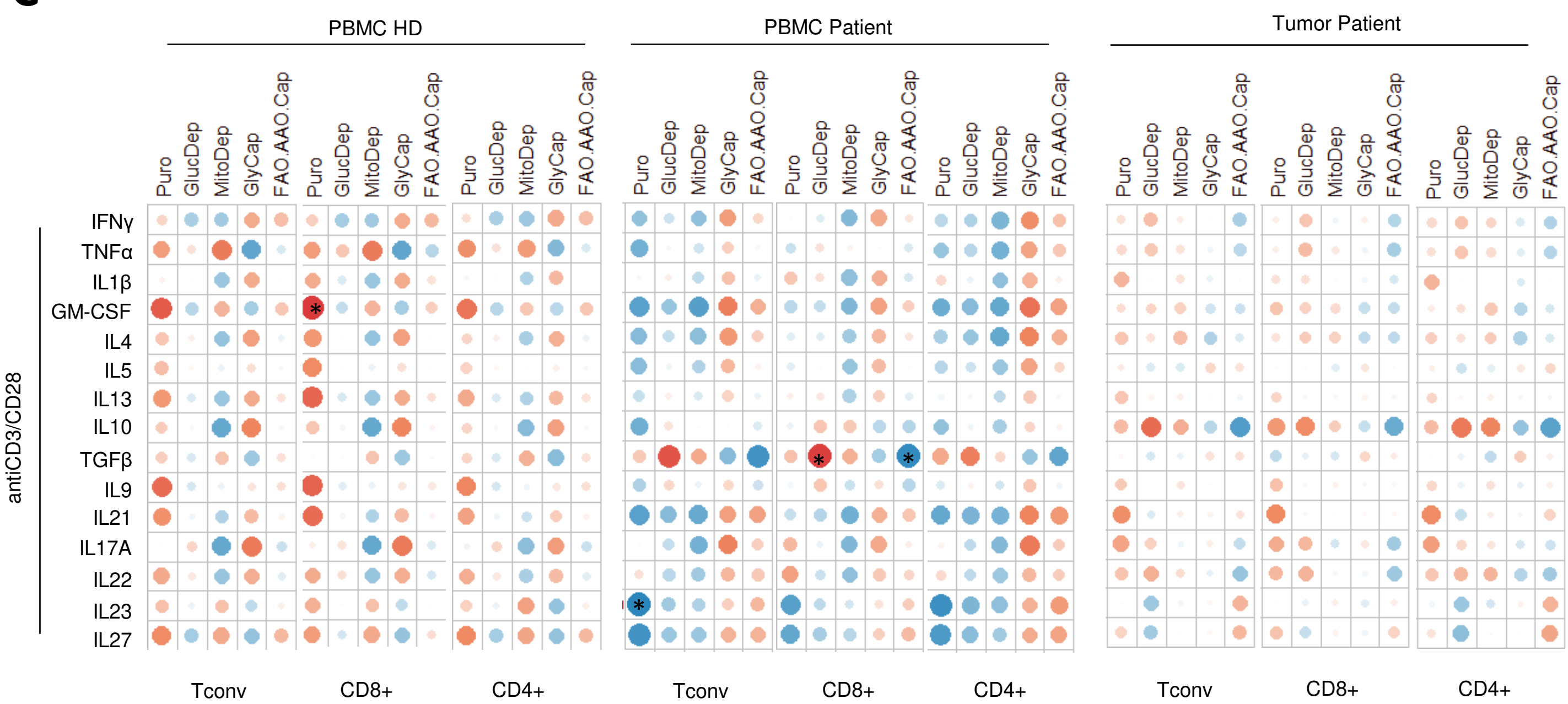

Supplementary Figure 19

A

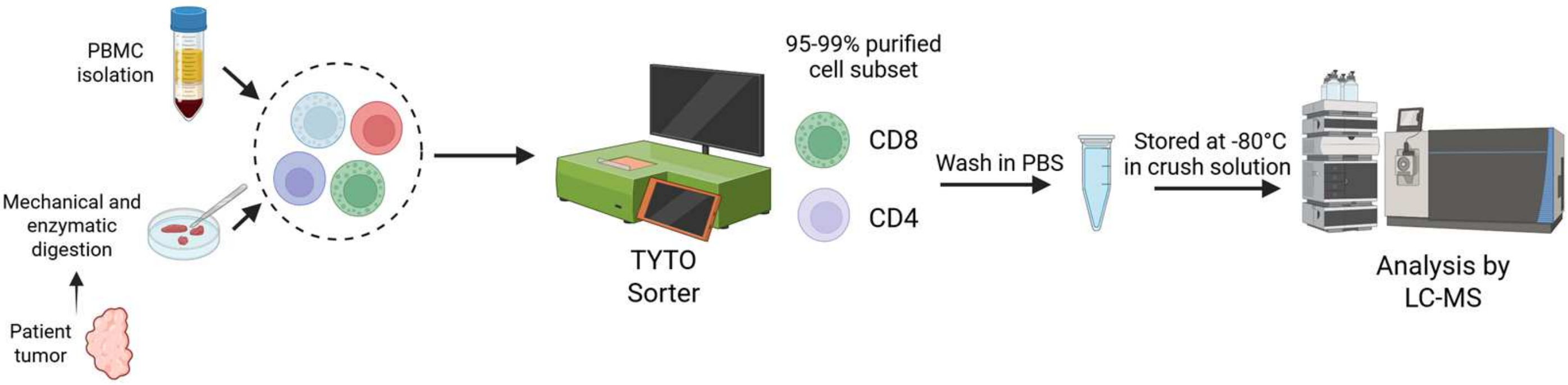

B

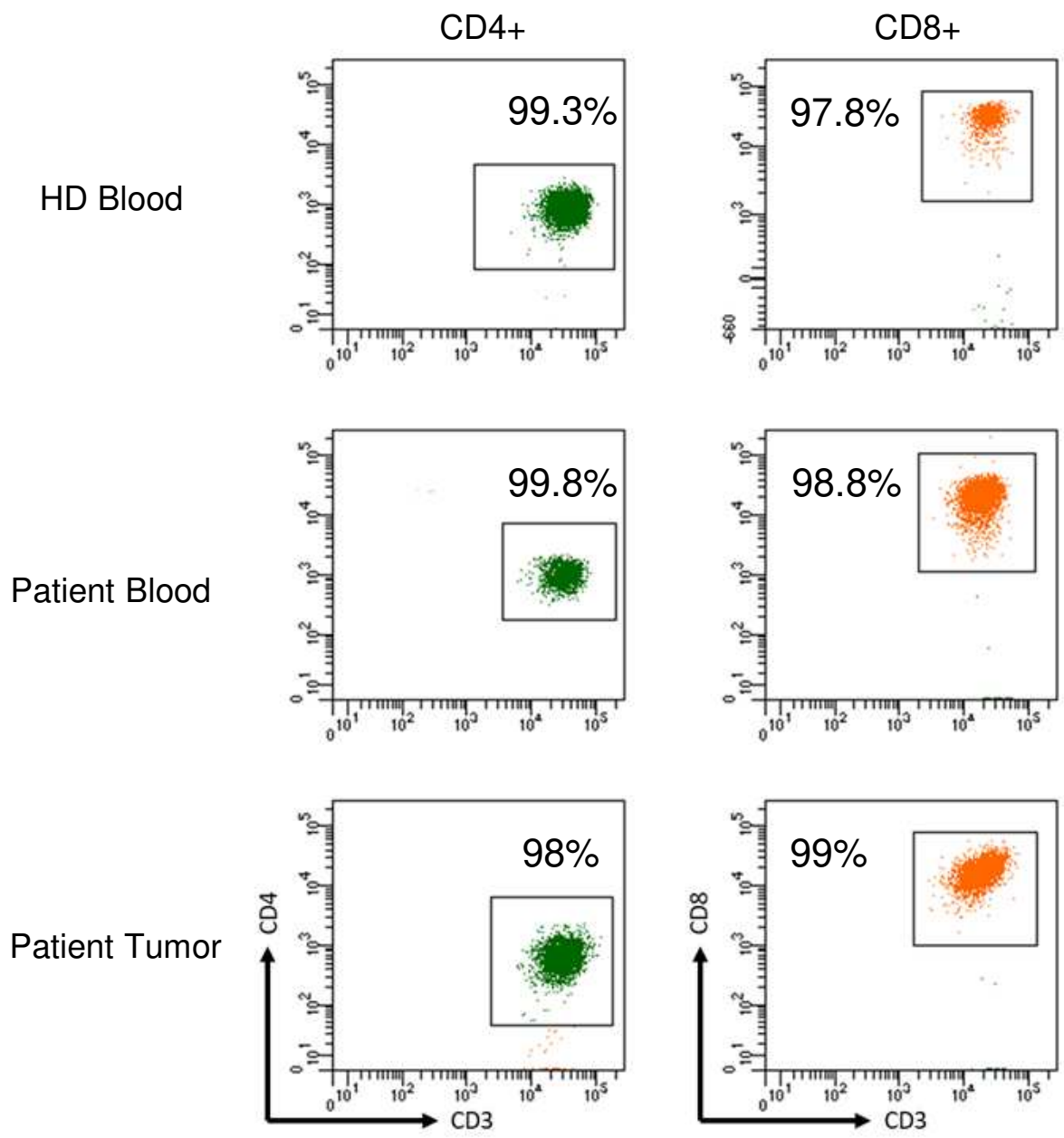

Supplementary Figure 20

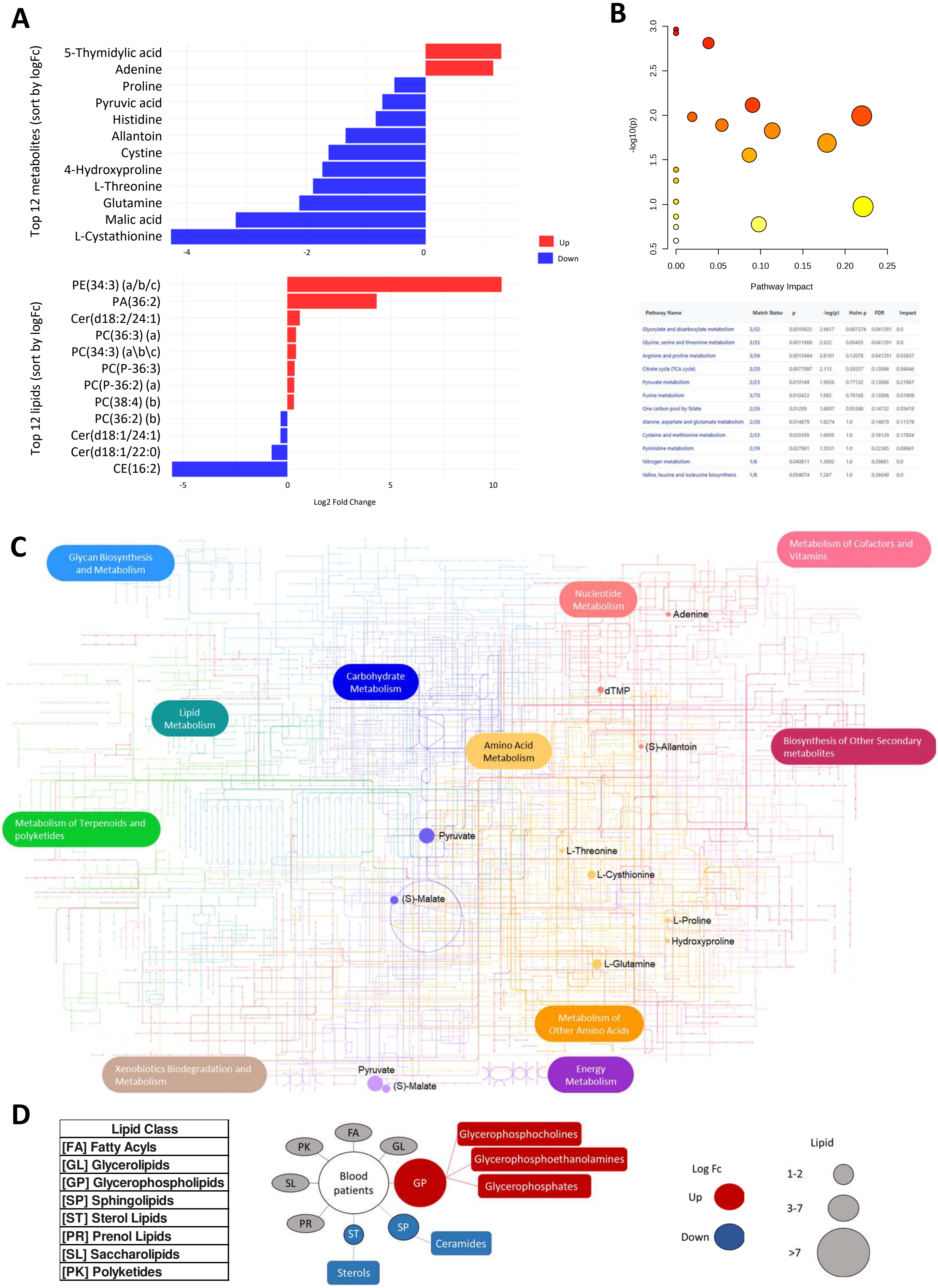

Pathway Name	Match Status	p	-log(p)	Holm p	FDR	Impact
Glyoxylate and dicarboxylate metabolism	3/32	0.0010922	2.9617	0.087374	0.041291	0.0
Glycine, serine and threonine metabolism	3/33	0.0011968	2.922	0.09455	0.041291	0.0
Arginine and proline metabolism	3/36	0.0015484	2.8101	0.12078	0.041291	0.03837
Citrate cycle (TCA cycle)	2/20	0.0077087	2.113	0.59357	0.13896	0.09046
Pyruvate metabolism	2/23	0.010149	1.9936	0.77132	0.13896	0.21967
Purine metabolism	3/70	0.010422	1.982	0.78168	0.13896	0.01908
One carbon pool by folate	2/26	0.01289	1.8897	0.95388	0.14732	0.05419
Alanine, aspartate and glutamate metabolism	2/28	0.014879	1.8274	1.0	0.14879	0.11378
Cysteine and methionine metabolism	2/33	0.020395	1.6905	1.0	0.18129	0.17854
Pyrimidine metabolism	2/39	0.027981	1.5531	1.0	0.22385	0.08661
Nitrogen metabolism	1/6	0.040811	1.3892	1.0	0.29681	0.0
Valine, leucine and isoleucine biosynthesis	1/8	0.054074	1.267	1.0	0.36049	0.0

Lipid Class
[FA] Fatty Acyls
[GL] Glycerolipids
[GP] Glycerophospholipids
[SP] Sphingolipids
[ST] Sterol Lipids
[PR] Prenol Lipids
[SL] Saccharolipids
[PK] Polyketides

Supplementary Figure 21

A

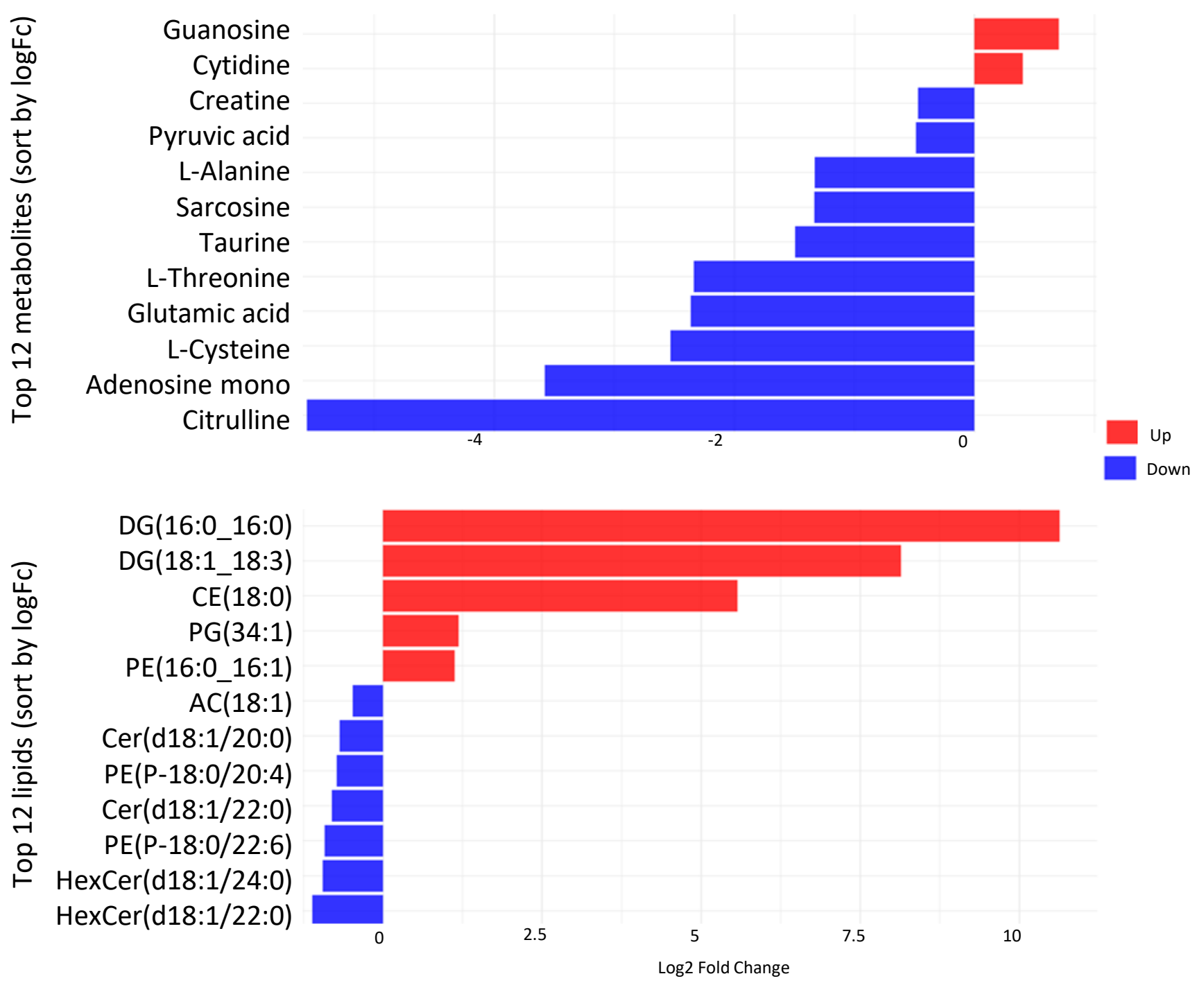

B

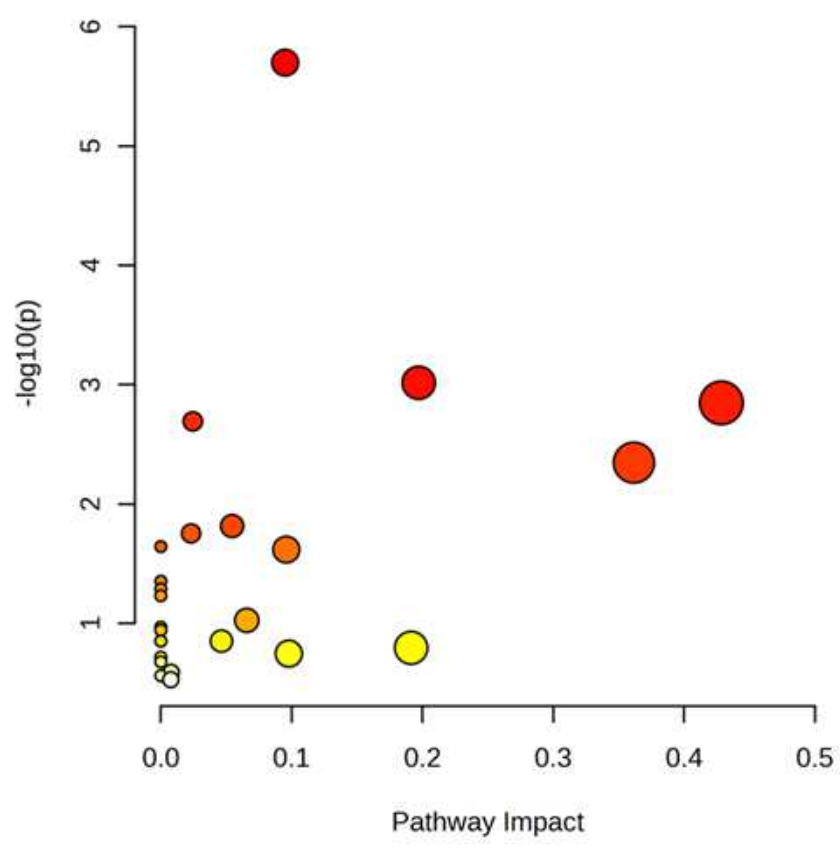

Pathway Name	Match Status	p	-log(p)	Holm p	FDR	Impact
Glycine, serine and threonine metabolism	5/33	2.001E-6	5.6988	1.6008E-4	1.6008E-4	0.09505
Alanine, aspartate and glutamate metabolism	3/28	9.6509E-4	3.0154	0.076242	0.037944	0.19712
Taurine and hypotaurine metabolism	2/8	0.0014229	2.8468	0.11099	0.037944	0.42857
Arginine and proline metabolism	3/36	0.0020326	2.692	0.15651	0.040651	0.02442
Arginine biosynthesis	2/14	0.0045093	2.3459	0.34271	0.072149	0.3617
One carbon pool by folate	2/26	0.015314	1.8149	1.0	0.20185	0.0545
Glutathione metabolism	2/28	0.017662	1.753	1.0	0.20185	0.02309
Glyoxylate and dicarboxylate metabolism	2/32	0.02279	1.6422	1.0	0.21475	0.0
Cysteine and methionine metabolism	2/33	0.024159	1.6169	1.0	0.21475	0.09592
Nitrogen metabolism	1/6	0.044451	1.3521	1.0	0.35561	0.0
Thiamine metabolism	1/7	0.051681	1.2867	1.0	0.37586	0.0
Valine, leucine and isoleucine biosynthesis	1/8	0.058861	1.2302	1.0	0.3924	0.0

C

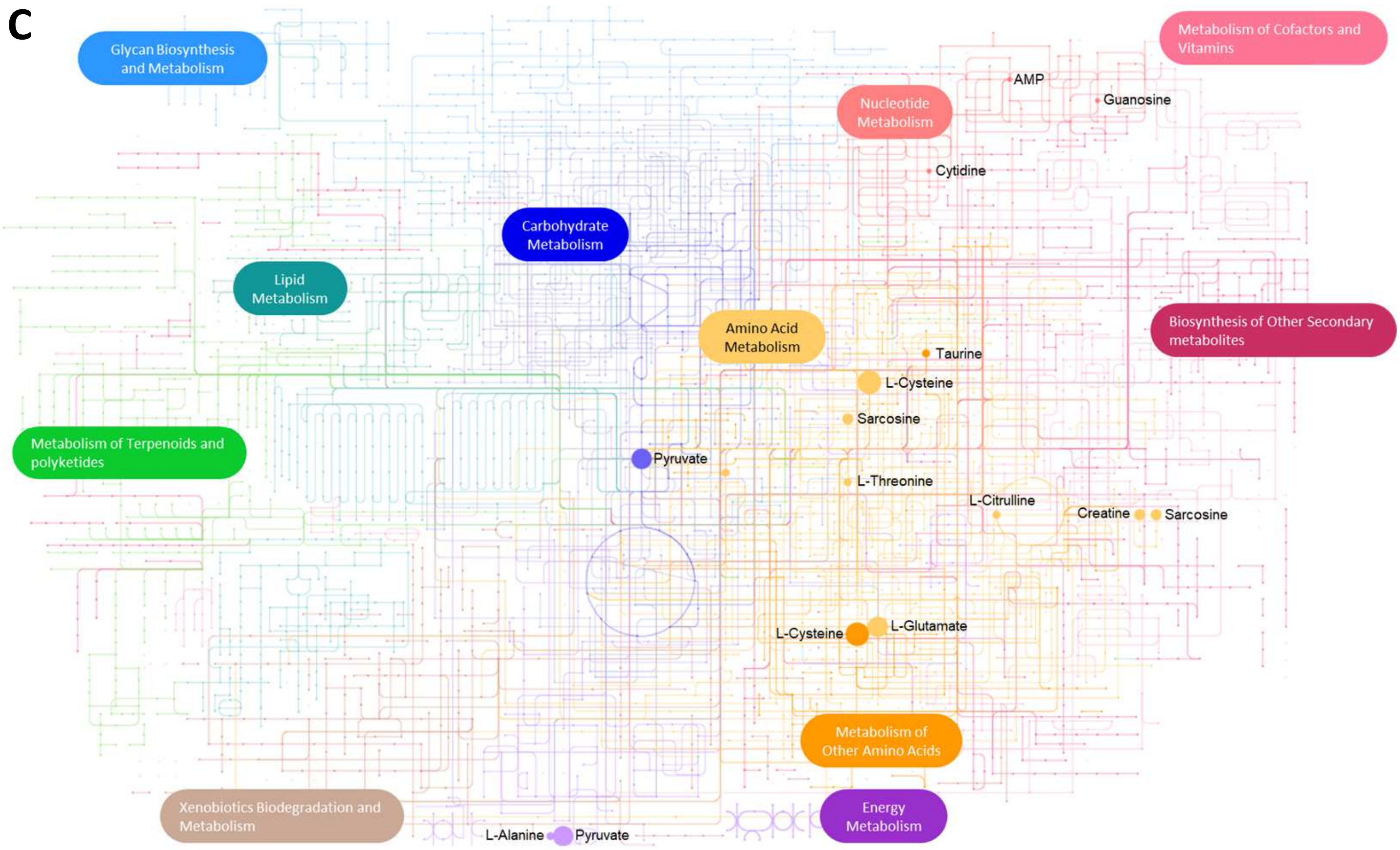

D

Lipid Class
[FA] Fatty Acyls
[GL] Glycerolipids
[GP] Glycerophospholipids
[SP] Sphingolipids
[ST] Sterol Lipids
[PR] Prenol Lipids
[SL] Saccharolipids
[PK] Polyketides

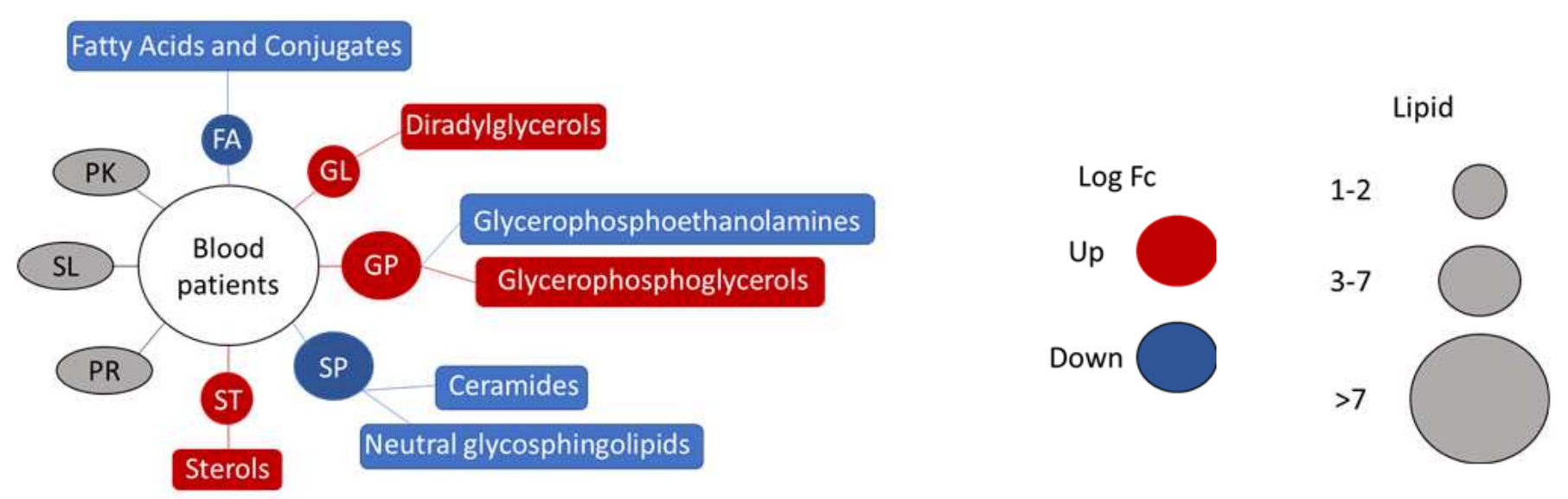

Supplementary Figure 22

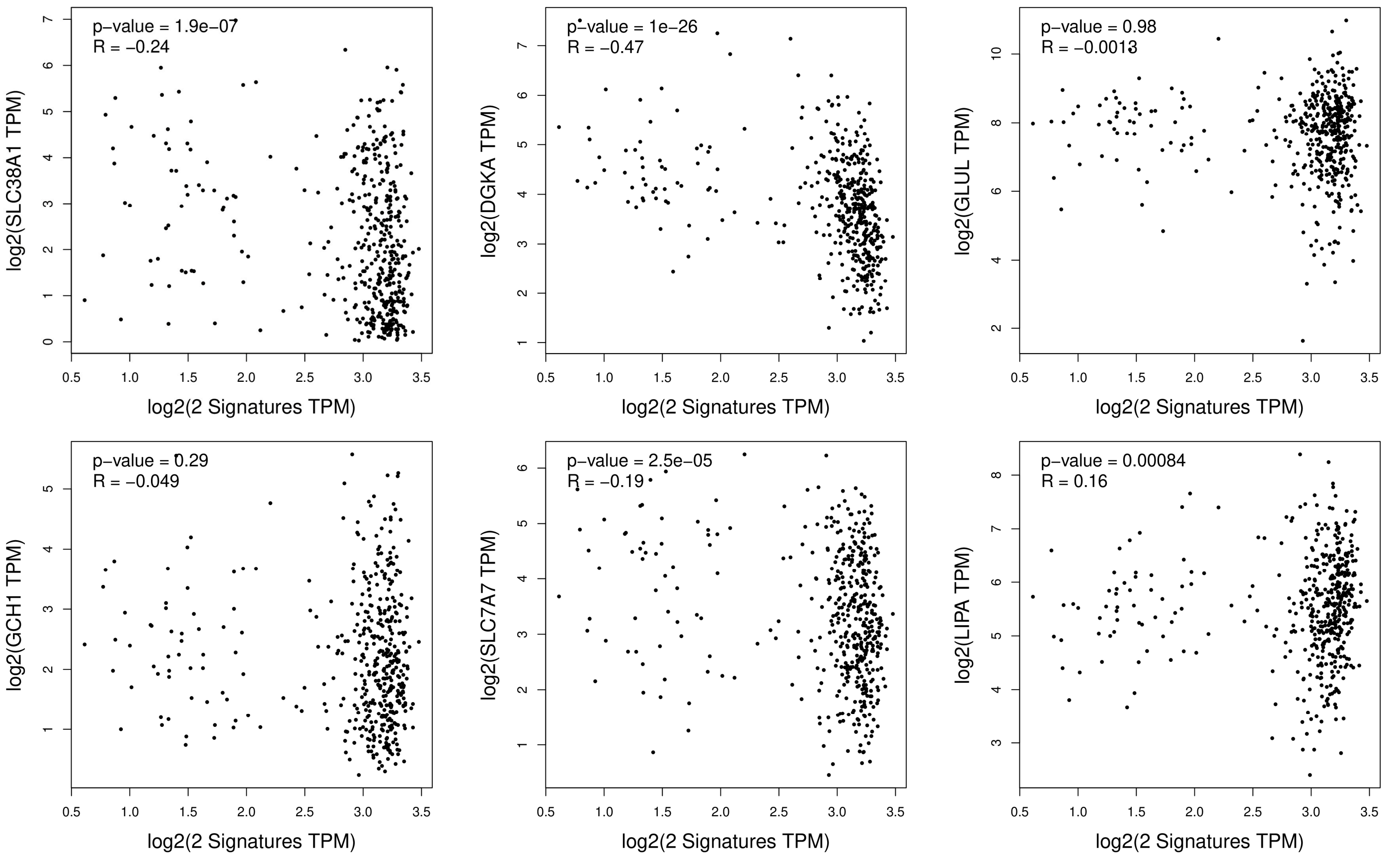

Supplement: Supplementary file 1 [file IMM-179-486-s002.pdf]
