# Supplementary material for: Metabolic Profiling of Circulating and Tumour‐Infiltrating T Cells Uncovers Bioenergetic Checkpoints Linked With Immune and Clinical Skewing in Melanoma Patients
Source: Immunology. 2026 Aug 4;179(3):486–515. doi: 10.1111/imm.70185 (PMC13636174; doi:10.1111/imm.70185)
Supplement: Supplementary file 2 [file IMM-179-486-s001.docx]

# Supplementary Figure legends

**Supplementary Figure 1: Measurement of the metabolic parameters of T cell subsets using SCENITH method**

PBMC from HDs and melanoma patients as well as CD45^+^ tumor-infiltrating cells from patients were submitted to the SCENITH method to depict the metabolism of T cell subsets by flow cytometry. (A) Gating strategy of T cell subsets. Cell debris and cell doublets were first excluded using the FSC and SSC parameters. Viable (Live&Dead^-^) CD45^+^ cells were gated prior to CD3^+^ gating. From total CD3^+^ T cells, conventional T cells (Tconv) were defined as CD45^+^ CD3^+^ PanTCRγδ^-^ TCRδ2^-^. CD8^+^ and CD4^+^ T cell subsets were further depicted as CD3^+^CD8^+^ and CD3^+^CD8^-^ respectively. (B) Flow cytometry histograms depicting the incorporation of puromycin by T cell subsets in the presence of the inhibitors of glycolysis (2-Deoxy-Glucose, DG) or OXPHOS (Oligomycin, O) metabolic pathways or both inhibitors, required for SCENITH analysis.

**Supplementary Figure 2: Metabolic features of patients’ T cell subsets according to clinical outcome, disease stage and previous treatment.**

PBMC from HDs and melanoma patients as well as CD45^+^ tumor-infiltrating cells from patients were isolated and the metabolism of T cell subsets was depicted by flow cytometry using the SCENITH method. (A) Comparative PFS and OS (from diagnosis time) of patients displaying a high or low mitochondrial dependency, or a high or low glycolytic capacity of circulating TCD4^+^ cells (5-6 patients per group). Groups were separated using the median of the corresponding parameter for circulating T CD4^+^ cells (glucose dependency [46.1%], mitochondrial dependency [65.5%], glycolytic capacity [34.5%], FAO/AAO capacity [53.9%]). Comparisons performed using Log-Rank test. (B, C) Comparative levels of (B) global metabolism (Mean Fluorescence Intensity (MFI) of puromycin) and (C) glycolytic or mitochondrial dependencies / capacities of T cell subsets from patient tumor according to prior treatment (immunotherapy or targeted therapy) or not. The central line indicates median. *P*-values were calculated using non-parametric two-tailed Mann-Whitney tests (for global protein synthesis) and two-way ANOVA with the Geisser-Greenhouse correction (for metabolic profiles).

**Supplementary Figure 3: Phenotypic profiling of T cell subsets**

PBMC from HDs and melanoma patients as well as CD45^+^ tumor-infiltrating cells from patients were isolated, and the phenotypic features of T cell subsets were depicted by flow cytometry. (A) Representative dot plots displaying expression of activation markers (CD69 and CD25) and ICPs (CTLA4, PD1, LAG3, TIM3, ICOS, TIGIT, OX40, 41BB, CD27 and GITR) on T cells from HD blood, patient blood and patient tumor (pre-gated on alived CD45^+^ CD3^+^ cells). (B) Profile of expression of activation markers and immune checkpoints in Tconv cells from HD blood (blue, n=11-25), patient blood (green, n=10-25) and tumor (pink, n=11-25). (C, D) Correlation matrix between the phenotypic parameters of T cell subsets (activation markers and ICPs) for (C) patient blood and (D) patient tumor. *P*-values were calculated using a Spearman test followed by Holm correction. Only significant statistics are displayed on graphs. **P* ≤ 0.01.

**Supplementary Figure 4: Link between the metabolic and phenotypic profiles of Tconv cells**

PBMC from HDs and melanoma patients as well as CD45^+^ tumor-infiltrating cells from patients were isolated, and the metabolism of T cell subsets was depicted using the SCENITH method together with their phenotypic features by flow cytometry. (A) Correlation matrix between the metabolic parameters of Tconv cells (global metabolism “puro” as well as glycolytic/mitochondrial dependencies and capacities) and their expression of activation markers and ICPs for HD blood (n=11-25), patient blood (n=9-24) or tumor (n=11-25). *P*-values were calculated using a Spearman test followed by Holm correction. Only significant statistics are displayed on graphs. **P* ≤ 0.01. (B, C) Heat map displaying the (B) global metabolism and (C) glycolytic or mitochondrial dependencies / capacities of Tconv cells, stratified according to their expression (+) or not (-) of activation markers and ICPs for HD blood (blue, n=10-11), patient blood (green, n=9-10) or tumor (pink, n=11-13). (D) Metabolic profiles were enumerated as follows: (1) glucose dependency, (2) mitochondrial dependency, (3) glycolytic capacity, and (4) FAO/AAO capacity. (D, E) Heat map displaying the expression levels of activation markers and ICPs on Tconv cells stratified according to their low or high (D) global metabolism and (E) glycolytic or mitochondrial dependencies / capacities, for HD blood (blue, n=10-11), patient blood (green, n=9-10) or tumor (pink, n=11-13). Groups were separated using the median of the metabolic parameter Tconv cells.

**Supplementary Figure 5: Comparative metabolism of CD8^+^ T cells according to their expression or not of activation markers and ICPs**

PBMC from HDs and melanoma patients as well as CD45^+^ tumor-infiltrating cells from patients were isolated, and the metabolism of CD8^+^ T cells was depicted using the SCENITH method together with their phenotypic features by flow cytometry. (A, B, C, D) Comparative levels of global metabolism (left panel) and glycolytic or mitochondrial dependencies / capacities (right panels) of CD8^+^ T cells according to their expression (+) or not (-) of activation markers (A) and ICPs (B, C, D) for HD blood (blue, n=10-11), patient blood (green, n=9-10) or tumor (pink, n=11-13). *P*-values were calculated using RM two-way ANOVA with Sidak’s multiple comparison post-test (for intra-group comparisons) or non-parametric two-way ANOVA or a mixed effect model followed by Tukey’s multiple comparison post-tests (for inter-groups comparisons).

**Supplementary Figure 6: Comparative metabolism of CD4^+^ T cells according to their expression or not of activation markers and ICPs**

PBMC from HDs and melanoma patients as well as CD45^+^ tumor-infiltrating cells from patients were isolated, and the metabolism of CD4^+^ T cells was depicted using the SCENITH method together with their phenotypic features by flow cytometry. (A, B, C, D) Comparative levels of global metabolism (left panel) and glycolytic or mitochondrial dependencies / capacities (right panels) of CD4^+^ T cells according to their expression (+) or not (-) of activation markers (A) and ICPs (B, C, D) for HD blood (blue, n=10-11), patient blood (green, n=9-10) or tumor (pink, n=11-13). *P*-values were calculated using RM two-way ANOVA with Sidak’s multiple comparison post-test (for intra-group comparisons) or non-parametric two-way ANOVA or a mixed effect model followed by Tukey’s multiple comparison post-tests (for inter-groups comparisons).

**Supplementary Figure 7: Link between the metabolic status of T cell subsets and the composition of their secretome at steady state**

PBMC from HDs and melanoma patients as well as CD45^+^ tumor-infiltrating cells from patients were cultured for 20h, and their metabolism was depicted using the SCENITH method simultaneously to measurement of their secretome by Luminex. (A) Comparative levels of secreted cytokines from HD blood (blue, n=10), patient blood (green, n=10) or tumor (pink, n=12). Bars indicate median. *P*-values were calculated using non-parametric two-way ANOVA or a mixed effect model followed by Tukey’s multiple comparison post-tests. Only significant statistics are displayed on graphs. ***P* ≤ 0.01, *****P* ≤ 0.0001. (B) Correlation matrix between the metabolic parameters of T cell subsets (global metabolism (MFI of puromycin) and glycolytic or mitochondrial dependencies / capacities) and cytokine secretion levels from HD blood (blue, n=9), patient blood (green, n=9) or tumor (pink, n=9). *P*-values were calculated using a Spearman test followed by Holm correction. Only significant statistics are displayed on graphs. **P* ≤ 0.01.

**Supplementary Figure 8: Remodeling of the expression profiles of activation markers and immune checkpoints by T cell subsets upon stimulation**

PBMC from HDs and melanoma patients as well as CD45^+^ tumor-infiltrating cells from patients were stimulated or not with either PMA/iono or anti-CD3/CD28 antibodies, and their phenotypic features were depicted by flow cytometry. Profile of expression of activation markers and immune checkpoints in CD8^+^ and CD4^+^ T cells in the different conditions of stimulation for HD blood (blue, n=10-11), patient blood (green, n=8-10) and tumor (pink, n=8-13). *P*-values were calculated using non-parametric two-way ANOVA or a mixed effect model followed by Tukey’s multiple comparison post-tests. Only significant statistics are displayed on graphs. **P* ≤ 0.05, ***P* ≤ 0.01, ****P* ≤ 0.001, *****P* ≤ 0.0001.

**Supplementary Figure 9: The remodeling of the expression profiles of activation markers and immune checkpoints by T cell subsets upon stimulation is linked with metabolic reprogramming in melanoma**

PBMC from HDs and melanoma patients as well as CD45^+^ tumor-infiltrating cells from patients were stimulated or not with either PMA/iono or anti-CD3/CD28 antibodies, and the metabolism of T cell subsets was depicted using the SCENITH method along with their phenotypic features by flow cytometry. (A) Correlation matrix between the metabolic parameters of T cell subsets (global metabolism “puro” as well as glycolytic/mitochondrial dependencies and capacities) and their expression of activation markers and ICPs upon anti-CD3/CD28 stimulation for HD blood, patient blood or tumor. *P*-values were calculated using a Spearman test followed by Holm correction. Only significant statistics are displayed on graphs. **P* ≤ 0.01. (B, C) Heat map displaying the (B) global metabolism and (C) glycolytic or mitochondrial dependencies / capacities of T cell subsets, stratified according to their expression (+) or not (-) of activation markers and ICP upon anti-CD3/CD28 stimulation for HD blood (blue, n=9-10), patient blood (green, n=8-9) or tumor (pink, n=8-12). (C) Metabolic profiles were enumerated as follows: (1) glucose dependency, (2) mitochondrial dependency, (3) glycolytic capacity, and (4) FAO/AAO capacity. (D, E) Heat map displaying the expression levels of activation markers and ICPs on ~~Tconv and~~ T cell subsets stratified according to their low or high (D) global metabolism and (E) glycolytic or mitochondrial dependencies / capacities upon anti-CD3/CD28 stimulation for HD blood (blue, n=10-11), patient blood (green, n=8-9) or tumor (pink, n=8-12). Groups were separated using the median of the metabolic parameter in the corresponding T cell subsets.

**Supplementary Figure 10: Comparative metabolism of CD8^+^ T cells according to their expression or not of activation markers and ICPs upon PMA/iono stimulation**

PBMC from HDs and melanoma patients as well as CD45^+^ tumor-infiltrating cells from patients were stimulated with PMA/iono, and the metabolism of CD8^+^ T cells was depicted using the SCENITH method together with their phenotypic features by flow cytometry. (A, B, C, D) Comparative levels of global metabolism (left panel) and glycolytic or mitochondrial dependencies / capacities (right panels) of CD8^+^ T cells according to their expression (+) or not (-) of activation markers (A) and ICPs (B, C, D) upon PMA/iono stimulation for HD blood (blue, n=9-10), patient blood (green, n=8-9) or tumor (pink, n=8-12). *P*-values were calculated using RM two-way ANOVA with Sidak’s multiple comparison post-test (for intra-group comparisons) or non-parametric two-way ANOVA or a mixed effect model followed by Tukey’s multiple comparison post-tests (for inter-groups comparisons).

**Supplementary Figure 11: Comparative metabolism of CD4^+^ T cells according to their expression or not of activation markers and ICPs upon PMA/iono stimulation**

PBMC from HDs and melanoma patients as well as CD45^+^ tumor-infiltrating cells from patients were stimulated with PMA/iono, and the metabolism of CD4^+^ T cells was depicted using the SCENITH method together with their phenotypic features by flow cytometry. (A, B, C, D) Comparative levels of global metabolism (left panel) and glycolytic or mitochondrial dependencies / capacities (right panels) of CD4^+^ T cells according to their expression (+) or not (-) of activation markers (A) and ICPs (B, C, D) upon PMA/iono stimulation for HD blood (blue, n=9-10), patient blood (green, n=8-9) or tumor (pink, n=8-12). *P*-values were calculated using RM two-way ANOVA with Sidak’s multiple comparison post-test (for intra-group comparisons) or non-parametric two-way ANOVA or a mixed effect model followed by Tukey’s multiple comparison post-tests (for inter-groups comparisons).

**Supplementary Figure 12: Comparative metabolism of CD8^+^ T cells according to their expression or not of activation markers and ICPs upon anti-CD3/CD28 stimulation**

PBMC from HDs and melanoma patients as well as CD45^+^ tumor-infiltrating cells from patients were stimulated with anti-CD3/CD28, and the metabolism of CD8^+^ T cells was depicted using the SCENITH method together with their phenotypic features by flow cytometry. (A, B, C, D) Comparative levels of global metabolism (left panel) and glycolytic or mitochondrial dependencies / capacities (right panels) of CD8^+^ T cells according to their expression (+) or not (-) of activation markers (A) and ICPs (B, C, D) upon anti-CD3/CD28 stimulation for HD blood (blue, n=9-10), patient blood (green, n=8-9) or tumor (pink, n=8-12). *P*-values were calculated using RM two-way ANOVA with Sidak’s multiple comparison post-test (for intra-group comparisons) or non-parametric two-way ANOVA or a mixed effect model followed by Tukey’s multiple comparison post-tests (for inter-groups comparisons).

**Supplementary Figure 13: Comparative metabolism of CD4^+^ T cells according to their expression or not of activation markers and ICPs upon anti-CD3/CD28 stimulation**

PBMC from HDs and melanoma patients as well as CD45^+^ tumor-infiltrating cells from patients were stimulated with anti-CD3/CD28, and the metabolism of CD8^+^ T cells was depicted using the SCENITH method together with their phenotypic features by flow cytometry. (A, B, C, D) Comparative levels of global metabolism (left panel) and glycolytic or mitochondrial dependencies / capacities (right panels) of CD4^+^ T cells according to their expression (+) or not (-) of activation markers (A) and ICPs (B, C, D) upon anti-CD3/CD28 stimulation for HD blood (blue, n=9-10), patient blood (green, n=8-9) or tumor (pink, n=8-12). *P*-values were calculated using RM two-way ANOVA with Sidak’s multiple comparison post-test (for intra-group comparisons) or non-parametric two-way ANOVA or a mixed effect model followed by Tukey’s multiple comparison post-tests (for inter-groups comparisons).

**Supplementary Figure 14: Functional orientation of T cell subsets upon stimulation**

PBMC from HDs and melanoma patients as well as CD45^+^ tumor-infiltrating cells from patients were stimulated or not with either PMA/iono or anti-CD3/CD28 antibodies, and their metabolism was depicted using the SCENITH method simultaneously to intracellular cytokine measurements by flow cytometry. (A) Profile of cytokine expression by CD8^+^ (upper panel) and CD4^+^ (lower panel) T cells in the different conditions of stimulation for HD blood (blue, n=8-10), patient blood (green, n=8-10) and tumor (pink, n=11-13). (B) Correlation matrix between the proportions of cytokine-expressing T cell subsets upon PMA/iono (left panel) or anti-CD3CD28 (right panel) stimulation. *P*-values were calculated using a Spearman test followed by Holm correction. Only significant statistics are displayed on graphs. **P* ≤ 0.01.

**Supplementary Figure 15: The cytokine expression profiles of T cell subsets upon stimulation is linked with metabolic reprogramming in melanoma**

PBMC from HDs and melanoma patients as well as CD45^+^ tumor-infiltrating cells from patients were stimulated or not with anti-CD3/CD28 antibodies, and their metabolism was depicted using the SCENITH method simultaneously to intracellular cytokine measurements by flow cytometry. (A) Correlation matrix between the metabolic parameters of T cell subsets (global metabolism “puro” as well as glycolytic/mitochondrial dependencies and capacities) and their cytokine expression upon anti-CD3/CD28 stimulation for HD blood (blue, n=10-11), patient blood (green, n=9-10) or tumor (pink, n=11-12). *P*-values were calculated using a Spearman test followed by Holm correction. Only significant statistics are displayed on graphs. **P* ≤ 0.01. (B) Heat map displaying the global metabolism of T cell subsets, stratified according to their expression (+) or not (-) of cytokines upon anti-CD3/CD28 for HD blood (blue, n=10-11), patient blood (green, n=8-10) or tumor (pink, n=12-13). (C) Heat map displaying the glycolytic or mitochondrial dependencies / capacities of T cell subsets, stratified according to their expression (+) or not (-) of cytokines upon anti-CD3/CD28 stimulation for HD blood (blue, n=10-11), patient blood (green, n=8-10) or tumor (pink, n=12-13). Metabolic profiles were enumerated as follows: (1) glucose dependency, (2) mitochondrial dependency, (3) glycolytic capacity, and (4) FAO/AAO capacity. (D) Heat map displaying cytokine expression levels of T cell subsets stratified according to their low or high global metabolism upon anti-CD3/CD28 stimulation for HD blood (blue, n=10-11), patient blood (green, n=9-10) or tumor (pink, n=11-13). Groups were separated using the median of the metabolic parameter in the corresponding T cell subsets. (E) Heat map displaying cytokine expression levels of T cell subsets stratified according to their low or high glycolytic or mitochondrial dependencies / capacities upon anti-CD3/CD28 stimulation for HD blood (blue, n=10-11), patient blood (green, n=9-10) or tumor (pink, n=11-13). Groups were separated using the median of the metabolic parameter in the corresponding T cell subsets. All significant statistics are displayed on corresponding graphs in Supplementary Figure 16, 17.

**Supplementary Figure 16: Comparative metabolism of CD8^+^ T cells according to their cytokine production profile upon stimulation**

PBMC from HDs and melanoma patients as well as CD45^+^ tumor-infiltrating cells from patients were stimulated with PMA/iono or anti-CD3/CD28, and the metabolism of CD8^+^ T cells was depicted using the SCENITH method simultaneously to intracellular cytokine measurements by flow cytometry. (A, B) Comparative levels of global metabolism (left panel) and glycolytic or mitochondrial dependencies / capacities (right panels) of CD8^+^ T cells according to their expression (+) or not (-) of cytokines upon PMA/iono (A) or anti-CD3/CD28 (B) stimulation for HD blood (blue, n=10-11), patient blood (green, n=8-10) or tumor (pink, n=12-13). *P*-values were calculated using RM two-way ANOVA with Sidak’s multiple comparison post-test (for intra-group comparisons) or non-parametric two-way ANOVA or a mixed effect model followed by Tukey’s multiple comparison post-tests (for inter-groups comparisons).

**Supplementary Figure 17: Comparative metabolism of CD4^+^ T cells according to their cytokine production profile upon stimulation**

PBMC from HDs and melanoma patients as well as CD45^+^ tumor-infiltrating cells from patients were stimulated with PMA/iono or anti-CD3/CD28, and the metabolism of CD4^+^ T cells was depicted using the SCENITH method simultaneously to intracellular cytokine measurements by flow cytometry. (A, B) Comparative levels of global metabolism (left panel) and glycolytic or mitochondrial dependencies / capacities (right panels) of CD4^+^ T cells according to their expression (+) or not (-) of cytokines upon PMA/iono (A) or anti-CD3/CD28 (B) stimulation for HD blood (blue, n=10-11), patient blood (green, n=8-10) or tumor (pink, n=12-13). *P*-values were calculated using RM two-way ANOVA with Sidak’s multiple comparison post-test (for intra-group comparisons) or non-parametric two-way ANOVA or a mixed effect model followed by Tukey’s multiple comparison post-tests (for inter-groups comparisons).

**Supplementary Figure 18: Link between the metabolic status of T cell subsets and the composition of their secretome under stimulation~~.~~**

PBMC from HDs and melanoma patients as well as CD45^+^ tumor-infiltrating cells from patients were stimulated or not with either PMA/iono or anti-CD3/CD28 antibodies, and their metabolism was depicted using the SCENITH method simultaneously to measurement of their secretome by Luminex. Comparative levels of secreted cytokines from HD blood (blue, n=10), patient blood (green, n=10) or tumor (pink, n=11-13) upon stimulation (PMA/iono or anti-CD3/CD28) or not. Bars indicate median. *P*-values were calculated using non-parametric two-way ANOVA or a mixed effect model followed by Tukey’s multiple comparison post-tests. Only significant statistics are displayed on graphs. **P* ≤ 0.05, ***P* ≤ 0.01, ****P* ≤ 0.001, *****P* ≤ 0.0001. (B, C) Correlation matrix between the metabolic parameters of T cell subsets (global metabolism (MFI of puromycin) and glycolytic or mitochondrial dependencies / capacities) and cytokine secretion levels from HD blood (blue, n=10), patient blood (green, n=10) or tumor (pink, n=12-13) upon PMA/iono (B) or anti-CD3/CD28 (C) stimulation. *P*-values were calculated using a Spearman test followed by Holm correction. Only significant statistics are displayed on graphs. **P* ≤ 0.01.

**Supplementary Figure 19: Strategy of Tyto sorting and LC-MS analysis**

PBMC from HDs and melanoma patients as well as CD45^+^ tumor-infiltrating cells from patients were submitted to Tyto sorting to purify CD4^+^ and CD8^+^ T cells. Metabolomic and lipidomic analysis were then performed by LC-MS. (A) Experimental design. Created in BioRender. (B) Representative dot plots displaying the purity of CD4^+^ T cells (left panels) and CD8^+^ T cells (right panels) obtained after Tyto sorting from PBMC of HD as well as PBMC and tumor of patients (pre-gated on single alive CD45^+^ cells).

**Supplementary Figure 20: Metabolomic and lipidomic deregulations in circulating CD4^+^ T cells from patients**

CD4^+^ T cells were isolated from PBMC of HDs and melanoma patients as well as tumor-infiltrating immune cells from patients using Tyto sorter and conserved in methanol, H_2_O and formic acid solution at -80°C. Cell subset metabolomic and lipidomic was performed using LC-MS method. (A) Bar plots representing the top 12 metabolites and lipid changes sorted by log of Fold Change between HD blood (n=4) and patient blood (n=6) after *limma* analysis (Supplementary Table 3). Blue: downregulation, red: upregulation of the metabolites in patient’s tumor compared to HD blood. (B, C) Enrichment’s pathway and mapping of the top 12 changes in metabolites between HD blood (n=4) and patient blood (n=6) using MetaboAnalyst software. (D) Representation of the top 12 changes in lipids among lipid classes and subclasses for differences between HD blood (n=4) and patient blood (n=6).

**Supplementary Figure 21: Metabolomic and lipidomic deregulation in circulating CD8^+^ T cells from patients**

CD8^+^ T cells were isolated from PBMC from HDs and melanoma patients as well as CD45^+^ tumor-infiltrating cells from patients using Tyto sorter and conserved in methanol, H_2_O and formic acid solution at -80°C. Cell subset metabolomic and lipidomic was performed using LC-MS method. (A) Bar plots representing the top 12 metabolites and lipid changes sorted by log of Fold Change between HD blood (n=4) and patient blood (n=6) after *limma* analysis (Supplementary Table 3). Blue: downregulation, red: upregulation of the metabolites in patient’s tumor compared to HD blood. (B, C) Enrichment’s pathway and mapping of the top 12 changes in metabolites between HD blood (n=4) and patient blood (n=6) using MetaboAnalyst software. (D) Representation of the top 12 changes in lipids among lipid classes and subclasses for differences between HD blood (n=4) and patient blood (n=6).

**Supplementary Figure 22: Expression of the candidate enzymes and transporters connected to the perturbated metabolites in tumor-infiltrating T cells was not correlated with tumor cells**

Correlation of the gene expression of candidate enzymes and nutrient transporters with the melanoma specific *MLANA/MITF* genes using RNA sequencing data from the TCGA dataset (SKCM cohort, n=461 patients) using the GEPIA2 server.

**Supplementary Tables**

**Supplementary Table 1: Clinical features of patients used in the study**

NA: Not Available

ND: Not Done

N: LDH norm

**Supplementary Table 2: Results of Log-Rank tests run between the metabolic features of T cell subsets and patient’s PFS and OS**

**Supplementary Table 3: Differential abundance analysis of general metabolites found in CD4 and CD8 T cells between patient’s blood or tumor and HD’s blood**

List of top 12 metabolites differentially produced in CD4 and CD8 T cells between melanoma patients and HDs indicating the fold changes, p-values and adjusted p-values for multiple comparisons using the False Discovery Rate (FDR) method (*limma* analysis).

**Supplementary Table 4: Differential abundance analysis of lipids found in CD4 and CD8 T cells between patient’s blood or tumor and HD’s blood**

List of top 12 lipids differentially produced in CD4 and CD8 T cells between melanoma patients and HDs indicating the fold changes, p-values and adjusted p-values for multiple comparisons using the False Discovery Rate (FDR) method (*limma* analysis).

**Supplementary Table 5:**

**List of upstream and downstream enzymes as well as transporters connected with the metabolites that were found to be perturbated within tumor-infiltrating CD4^+^ and CD8^+^ T cells**

**Supplementary Table 6:**

**List of enzymes/transporters found to be linked with melanoma patients’ clinical outcome**
